# Unusual Flavones from *Primula macrocalyx* as Inhibitors of OAT1 and OAT3 and as Antifungal Agents against *Candida rugosa*

**DOI:** 10.1038/s41598-019-45728-5

**Published:** 2019-06-25

**Authors:** Xue Li, Xue Wang, Caiyu Li, Manana Khutsishvili, George Fayvush, Daniel Atha, Youcai Zhang, Robert P. Borris

**Affiliations:** 1 School of Pharmaceutical Science and Technology, Health Sciences Platform, Tianjin University, 92 Weijin Rd., Nankai District, Tianjin, 300072 China; 2 National Herbarium of Georgia, Ilia State University, Tbilisi, Georgia; 3 Institute of Botany, Armenian National Academy of Sciences, Yerevan, Armenia; 4 New York Botanical Garden, Bronx, New York, USA

**Keywords:** Natural products, Molecular medicine

## Abstract

A bioactivity guided program exploring the interaction of phytochemicals in the entire plant *Primula macrocalyx* with the organic anion transporters (OAT1 and OAT3) and microorganisms led to the elucidation of ten known flavones (**1**–**4**, **6**–**10**, **12**) and two previously undescribed flavones (**5**, **11**). The structures of the compounds were determined by extensive analysis of spectroscopic data, as well as by comparison with data from previous reports. Two known flavones (**9**, **12**) are reported for the first time from the family Primulaceae. All compounds were evaluated for inhibition of OAT1 and OAT3. Six flavones (**2**, **3**, **6**–**8**, **12**) showed potent inhibitory activity on OAT1, while seven flavones (**2**, **3**, **6**–**9**, **12**) showed marked inhibitory activity on OAT3, with IC_50_ ≤ 10.0 *µ*M. Antimicrobial activities of crude fractions against sixteen microorganisms were tested to give a target yeast strain *Candida rugosa* for further evaluation of MICs on the isolates. Three flavones (**7**, **8**, **12**) showed marked antifungal activity with MIC < 2.0 *µ*M. To our knowledge, this study is the first to evaluate these flavones as inhibitors of the OAT1 and OAT3, and as antifungal agents.

## Introduction

*Primula macrocalyx* Bunge (Primulaceae) is a perennial herbaceous plant, which has been widely used in folk medicine as an expectorant, diuretic, sedative, spasmolytic, and sudorific to treat a variety of maladies such as vitamin deficiency, colds, fever, headache, insomnia, paralysis, scurvy, tuberculosis, heart disease, rheumatism, and kidney diseases^1,2^. The dosage forms involving *P. macrocalyx* are diverse, including tinctures, decoctions, powders and teas^2^. Previous phytochemical investigations on *P. macrocalyx* have led to the isolation of flavones^2,3^, triterpene glycosides and saponins^4–6^, bisbibenzyl compounds^1–3^, salicylates and their derivatives^2^. The content of free and total fatty acids, mainly palmitic, octadecatetraenoic, linoleic, and linolenic, from the aerial part of *P. macrocalyx* were determined by GC and GC-MS^2^. While *P. macrocalyx* is rich in triterpene glycosides, the content of these compounds is dependant on the locality^7^. Similarly, the content of ascorbic acid and flavonols in this plant decreased with increasing elevation above sea level^8^. Moreover, the plants of the genus *Primula* are considered promising as an accessible raw plant source of triterpene saponins in Russia^9^. Modern pharmacologic research has shown that riccardin C is a potent inhibitor of NO synthesis^10^ and the related bisbibenzyl compounds having cytotoxic, antibacterial, and fungicidal activity were inhibitors of 5-lipoxygenase^1^. These chemical compositions may contribute to the medicinal properties mentioned above.

The organic anion transporters (OATs in humans or Oats in rodents) play key roles in the distribution and excretion of drugs^11^. Specifically, organic anion transporter 1 (OAT1) and 3 (OAT3), which are highly expressed in the kidney, play an important part in the renal elimination of a range of substrate molecules^12,13^. Moreover, both OAT1 and OAT3 are considered to be therapeutic targets for hypertension^14^. Research in mice suggests that Oat3 may mediate blood pressure regulation, so Oat3 inhibitors might be considered as potential antihypertensive agents^15^. The tincture of *P. macrocalyx* roots is widely used as a diuretic, and the tea of its flowers is drunk for kidney disease in folk medicine^2^, making the interaction between OAT1/3 and *P. macrocalyx* an attractive target for further investigation.

Recent years have seen a resurgence of interest in antimicrobial agents from plants due to their ethnomedicinal uses and low toxicity and side effects. Particularly, developing countries rely on plants for the treatment of infectious and non-infectious diseases^16^. *P. macrocalyx* powder is in ethnomedicinal use for the treatment of tuberculosis^1^. Herein, we screened four fractions (*n*-hexane-soluble, dichloromethane-soluble, *n*-butanol-soluble and water-soluble) of the methanol extract of *P. macrocalyx* on sixteen kinds of microorganisms as part of an ongoing search for new antimicrobial chemotypes.

In our preliminary studies, the dichloromethane soluble fraction of a methanol extract of entire plant of *P. macrocalyx* elicited marked inhibition of OAT1 and OAT3 *in vitro*, and potent antifungal activity against yeast strain *Candida rugosa*.

In the present study, a bioactivity guided fractionation was performed on the methanol extract of *P. macrocalyx* collected in Armenia, followed by structure determination of the isolated compounds based on LC-MS and NMR, leading to the elucidation of twelve flavones (**1**–**12**), including two previously undescribed compounds (**5**, **11**). To our knowledge, this study is the first to evaluate these flavones as inhibitors of the OAT1 and OAT3. These data may allow an initial elucidation of the structure activity relationships within this group, and may also provide a rational basis for the therapeutic applications of *P. macrocalyx* in traditional medicine. Additionally, the isolated antifungal agents could play a complementary role in the chemotherapy of fungal infections.

## Results

**Figure 1:**
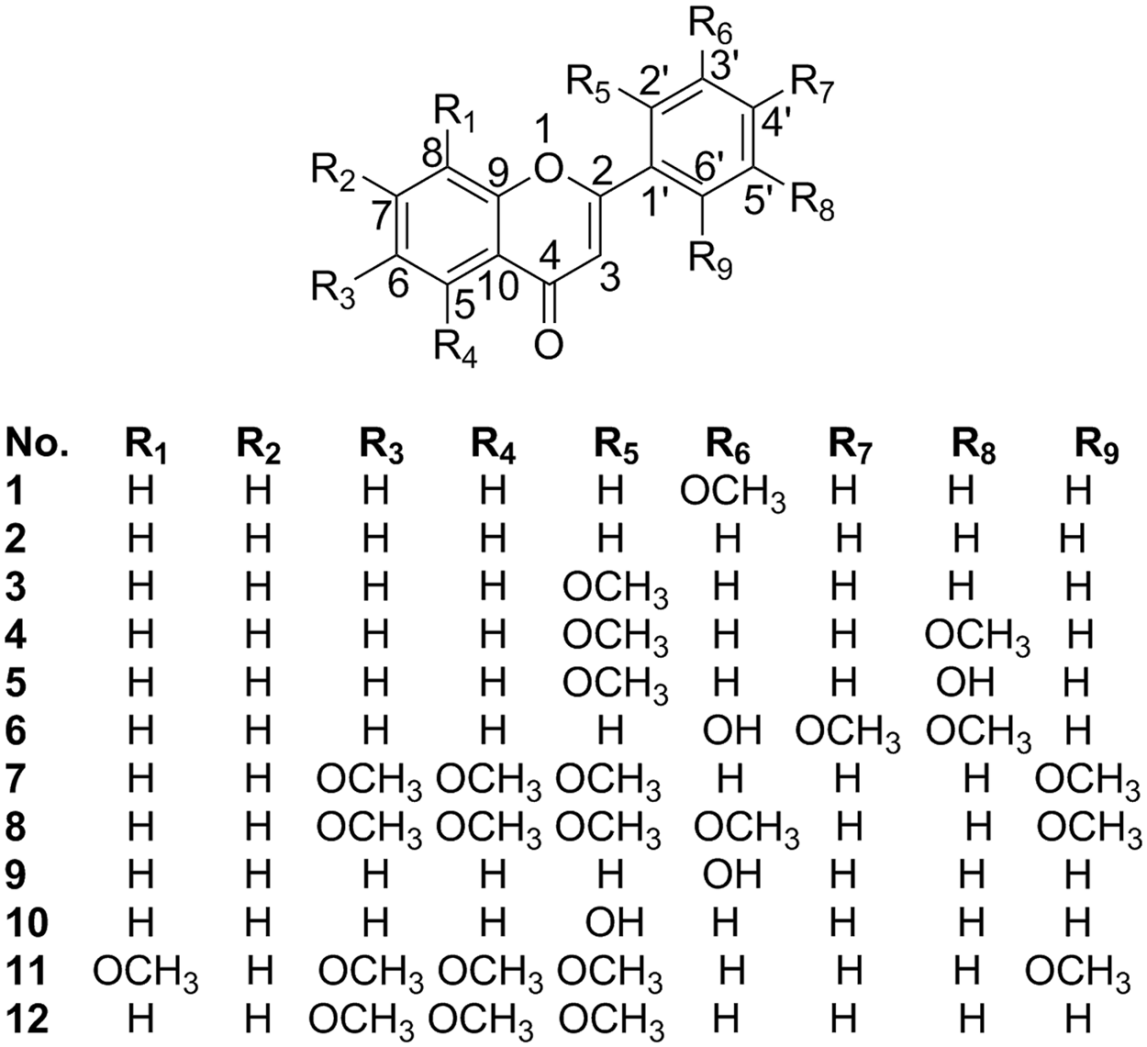
Structures of compounds **1**–**12**.

Samples comprising the whole plant of *P. macrocalyx* were extracted with methanol. The methanol-free extract was subjected to standard solvent partition, bioassay as well as a combination of different chromatographic techniques to afford twelve flavones (**1**–**12**), including two previously undescribed flavones (**5**, **11**) (Fig. 1).

**Figure 2:**
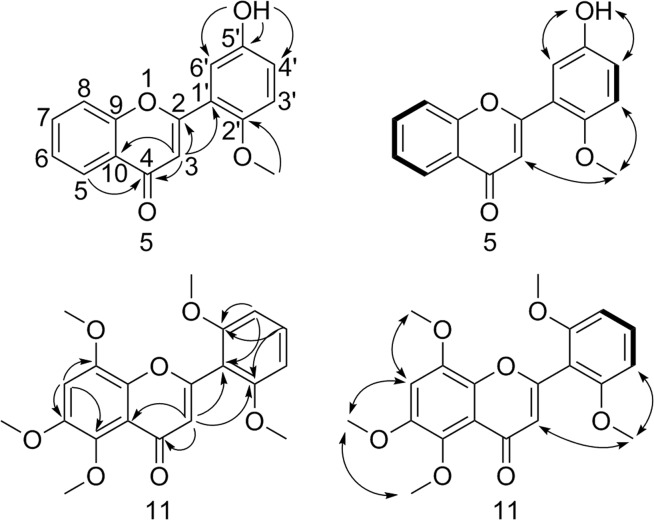
Key ^1^H → ^13^C HMBC (arrow), ^1^H → ^1^H NOESY (double arrow) and ^1^H → ^1^H COSY (solid bond) correlations of compounds **5** and **11**.

Compound **5** was obtained as a yellow amorphous powder. It showed two quasimolecular ions at m/z 269.0805 [M + H]^+^ (calcd. for C_16_H_13_O_4_ 269.0808) and 291.0625 [M + Na]^+^ (calcd. for C_16_H_12_O_4_Na 291.0633) corresponding to the molecular formula C_16_H_12_O_4_ in the HRESIMS. It was ascribed as having a flavone skeleton^17,18^ bearing methoxy and hydroxy substituents as shown by the ^1^H and ^13^C NMR spectroscopic analysis (Table 1). In the COSY spectrum (Fig. 2), the correlations: *δ*_H_ 6.96 (H-4′) and *δ*_H_ 7.08 (H-3′), *δ*_H_ 7.70 (H-8) and *δ*_H_ 7.82 (H-7), *δ*_H_ 7.82 (H-7) and *δ*_H_ 7.49 (H-6), and *δ*_H_ 7.49 (H-6) and *δ*_H_ 8.04 (H-5), were observed, which established two spin systems of **5**. Among them, the characteristic coupling pattern observed at *δ*_H_ 6.96 (1 H, dd, *J* = 8.9, 3.1 Hz, H-4′), 7.08 (1 H, d, *J* = 8.9 Hz, H-3′), and 7.31 (1 H, d, *J* = 3.1 Hz, H-6′) in the ^1^H NMR spectrum indicated the presence of a B-ring with a typical ABX system. The methoxy protons at *δ*_H_ 3.83 showed HMBC correlations (Fig. 2) with the carbon at *δ*_C_ 150.8, and NOESY correlations (Fig. 2) with *δ*_H_ 6.93 (H-3) and *δ*_H_ 7.08 (H-3′), establishing the location of this substituent group at C-2′. In addition, the downfield hydroxyl was located at C-5′ in the B-ring, supported by HMBC correlations between *δ*_H_ 9.41 and C-4′ (*δ*_C_ 119.3), C-6′ (*δ*_C_ 115.0) and C-5′ (*δ*_C_ 151.1), and NOESY correlations of *δ*_H_ 9.41 with *δ*_H_ 7.31 and *δ*_H_ 6.96. If the hydroxyl group had been located at C-4′ rather than C-5′, H-3′ would be flanked by two oxygen containing substituents and expected farther upfield around *δ*_H_ 6.7 PPM^19^. Additionally, this proton would be expected to show NOESY correlations with both the methoxy protons and the proton of the hydroxy group. This is clearly not the case.

**Table 1:** ^13^C (150 MHz) and ^1^H (600 MHz) NMR Data of Compounds **5** and **11** (*δ* in ppm, *J* in Hz).

no.	5 (DMSO-*d*_6_)		11 (Acetone-*d*_6_)		11 (DMSO-*d*_6_)		11 (CDCl_3_)	
	*δ*_C_, type	*δ*_H_	*δ*_C_, type	*δ*_H_	*δ*_C_, type	*δ*_H_	*δ*_C_, type	*δ*_H_
2	160.5, C		159.5, C		158.5, C		158.8, C	
3	111.5, CH	6.93, s	115.6, CH	6.06, s	114.4, CH	6.07, s	115.5, CH	6.36, s
4	177.1, C		177.4, C		176.3, C		178.1, C	
5	124.7, CH	8.04, dd (7.9, 1.0)	140.9, C		138.7, C		140.8, C	
6	125.4, CH	7.49, dt (7.9, 1.0)	146.4, C		145.0, C		145.4, C	
7	134.3, CH	7.82, dt (7.9, 1.0)	104.6, CH	7.18, s	103.7, CH	7.21, s	104.3, CH	6.89, s
8	118.4, CH	7.70, d (7.9)	150.4, C		149.1, C		149.0, C	
9	155.8, C		143.2, C		141.2, C		143.2, C	
10	123.1, C		120.5, C		118.7, C		119.8, C	
1′	120.3, C		112.3, C		110.5, C		111.4, C	
2′	150.8, C		159.5, C		158.0, C		158.7, C	
3′	114.0, CH	7.08, d (8.9)	105.1, CH	6.78, d (8.6)	104.3, CH	6.79, d (8.5)	104.1, CH	6.60, d (8.4)
4′	119.3, CH	6.96, dd (8.9, 3.1)	133.1, CH	7.46, t (8.6)	132.4, CH	7.47, t (8.5)	132.0, CH	7.35, t (8.4)
5′	151.1, C		105.1, CH	6.78, d (8.6)	104.3, CH	6.79, d (8.5)	104.1, CH	6.60, d (8.4)
6′	115.0, CH	7.31, d (3.1)	159.5, C		158.0, C		158.7, C	
5-OCH_3_			61.6, CH_3_	3.78, s	61.1, CH_3_	3.68, s	62.0, CH_3_	3.90, s
6-OCH_3_			57.1, CH_3_	3.91, s	56.5, CH_3_	3.88, s	57.1, CH_3_	3.90, s
8-OCH_3_			57.5, CH_3_	3.92, s	56.9, CH_3_	3.89, s	57.7, CH_3_	3.92, s
2′-OCH_3_	56.3, CH_3_	3.83, s	56.5, CH_3_	3.80, s	56.1, CH_3_	3.74, s	56.0, CH_3_	3.76, s
6′-OCH_3_			56.5, CH_3_	3.80, s	56.1, CH_3_	3.74, s	56.0, CH_3_	3.76, s
5′-OH		9.41, s

In the region of *δ*_H_ 7.49–8.04, four adjacent aromatic protons of the A-ring were confirmed by ^1^H-^1^H COSY correlations described above and a characteristic coupling pattern located at *δ*_H_ 7.49 (1 H, dt, *J* = 7.9, 1.0 Hz, H-6), 7.70 (1 H, d, *J* = 7.9 Hz, H-8), 7.82 (1 H, dt, *J* = 7.9, 1.0 Hz, H-7), and 8.04 (1 H, dd, *J* = 7.9, 1.0 Hz, H-5), which indicated the presence of an unsubstituted flavone A-ring. HMBC correlations from H-5 (*δ*_H_ 8.04) to C-4 (*δ*_C_ 177.1), C-9 (*δ*_C_ 155.8) and C-7 (*δ*_C_ 134.3) were observed, establishing that these protons are situated at C-6, C-8, C-7, and C-5 in the A-ring, respectively. These results support assignment of the structure of **5** as 2′-methoxy-5′-hydroxyflavone.

Compound **11** was obtained as a pale yellow amorphous powder. Its molecular formula, C_20_H_20_O_7_, was determined by HRESIMS m/z 373.1275 [M + H]^+^ (calcd C_20_H_21_O_7_ 373.1282). On the basis of 1D and 2D NMR spectra recorded in acetone-*d*_6_ (Table 1), **11** was also found to have a flavone skeleton bearing five methoxy groups. The HMBC correlations (Fig. 2) from the aromatic proton at *δ*_H_ 7.18 to carbons bearing methoxy groups at *δ*_C_ 140.9, *δ*_C_ 146.4, and *δ*_C_ 150.4, and NOESY correlations (Fig. 2) between this aromatic singlet (*δ*_H_ 7.18) and the methoxy group protons at *δ*_H_ 3.91 and *δ*_H_ 3.92, and between one methoxy signal at *δ*_H_ 3.78 and another one at *δ*_H_ 3.91, indicated the presence of a 5,6,8-trimethoxy substituted A-ring. The characteristic proton singlet observed at *δ*_H_ 6.06 (H-3) showed HMBC interactions with C-1′ (*δ*_C_ 112.4), C-10 (*δ*_C_ 120.5), C-5 (*δ*_C_ 140.9 (w)), C-2 (*δ*_C_ 159.5) and C-4 (*δ*_C_ 177.4), also supported the position of substituents in the A-ring. The B-ring substitution of **11** was the same as that of the known 5,6,2′,6′-tetramethoxyflavone (**7**)^20^. From these data, **11** was characterized as 5,6,8,2′,6′-pentamethoxyflavone (**11**). The NMR data of **11** recorded in DMSO-*d*_6_ and CDCl_3_ are also provided in Table 1.

The remaining ten known flavones were identified by interpretation of their 1D and 2D NMR, HRESIMS and UV spectral data and comparison with data available in the literature, including 3′-methoxyflavone (**1**)^21^, flavone (**2**)^22^, 2′-methoxyflavone (**3**)^21^, 2′,5′-dimethoxyflavone (**4**)^20^, 3′-hydroxy-4′,5′-dimethoxyflavone (**6**)^20^, 5,6,2′,6′-tetramethoxyflavone (**7**)^20^, 5,6,2′,3′,6′-pentamethoxyflavone (**8**)^23^, 3′-hydroxyflavone (**9**)^24^, 2′-hydroxyflavone (**10**)^24^, 5,6,2′-trimethoxyflavone (**12**)^25^. Their ^1^H and ^13^C NMR data are shown in Tables 1–3 in supplementary information.

All compounds (purity > 95%) isolated from the active fractions of *P. macrocalyx* were tested for their inhibitory activity on OAT1 and OAT3. When evaluated as inhibitors of OAT1 and OAT3 (Table 2), these flavones showed a concentration dependent inhibition of the transporters with IC_50_’s ranging from 3 to 50 *µ*M. The observed activities were consistent with the activity originally observed in the crude extract.

**Table 2:** IC_50_ values for OAT1 and OAT3 inhibition and MIC values against *C. rugosa* of isolated compounds.

No.	Name	IC_50_ (*µ*M)^*a*^	IC_50_ (*µ*M)^*b*^	MICs (*µ*M)^*c*^
		OAT1	OAT3	*C. rugosa*
**1**	3′-methoxyflavone	—	—	—
**2**	Flavone	9.4	4.6	500
**3**	2′-methoxyflavone	2.9	6.3	—
**4**	2′,5′-dimethoxyflavone	32.9	12.1	—
**5**	2′-methoxy-5′-hydroxyflavone	26.9	27.3	—
**6**	3′-hydroxy-4′,5′-dimethoxyflavone	3.2	5.1	—
**7**	5,6,2′,6′-tetramethoxyflavone	8.4	7.3	0.4
**8**	5,6,2′,3′,6′-pentamethoxyflavone	10.0	7.4	1.2
**9**	3′-hydroxyflavone	11.4	8.0	—
**10**	2′-hydroxyflavone	—	18.8	—
**11**	5,6,8,2′,6′-pentamethoxyflavone	—	10.9	—
**12**	5,6,2′-trimethoxyflavone	9.6	2.9	2.0
	Probenecid	29.4	8.7	—
	Amphotericin B	—	—	0.4

^a^Compounds **1**, **10**, **11** showed <70% inhibition of 6-CF uptake up by OAT1 to 50 *µ*M.

^b^Compound **1** showed <70% inhibition of 6-CF uptake up by OAT3 to 50 *µ*M.

^c^Compounds **1**, 3–**6**, and **9**–**11** did not inhibit *C. rugosa* up to 500 *µ*M.

Sixteen microorganisms, including eight bacteria, *Staphylococcus aureus*, *Staphylococcus epidermis*, *Streptococcus mutans*, *Pseudomonas fluorescens*, *Enterococcus hirae*, *Moraxella* (*Branhamella*) *catarrhalis*, *Pseudomonas aeruginosa*, *Bacillus subtilis subsp*. *Spizizenii*, and eight fungi, *Candida albicans*, *Aspergillus niger*, *Saccharomyces kudriavzevii*, *Penicillium chrysogenum*, *Candida parapsilosis*, *Candida rugosa*, *Candida tropicalis* and *Rhizopus stolonifer*, were used to evaluate antimicrobial activities of the four crude fractions from *P. macrocalyx*, at a concentration of 1 mg/mL in DMSO, using an agar well diffusion assay. Results showed the dichloromethane fraction had marked antifungal activity against *Candida rugosa* with a 20 mm diameter zone of inhibition (positive control, amphotericin B: 11 mm). Bioactivity guided isolation was performed to isolate the active antifungal compounds. Minimal inhibitory concentration (MIC) determinations are summarized in Table 2.

## Discussion

Twelve flavones (**1**–**12**) have been isolated and identified from the active dichloromethane soluble fraction of the entire plant of *P. macrocalyx*. Among them, **5** and **11** are newly described. Compounds **1**–**3**, **6**, **7**, **10** are reported for the first time from this species while **4** and **8** have been reported previously from this plant^2^, and **6** was previously reported from *Primula veris*^20^. Compounds **9** and **12** are herein reported for the first time from family Primulaceae. Additionally, **9** is described for the first time as a natural product, having been previously described as a product of chemical synthesis or biotransformation^26,27^. Previous reports and our present research on the phytochemistry of this species indicate that flavones are characteristic chemical constituents in *P. macrocalyx*. The unusual substitution patterns of the newly described constituents may prove useful in further chemotaxonomic studies of *P. macrocalyx* or the Primulaceae in general.

Evaluation of the isolated compounds as OAT inhibitors showed that six flavones (**2**, **3**, **6**–**8**, **12**) showed good concentration-dependent inhibition on OAT1 mediated 6-CF uptake and seven flavones (**2**, **3**, **6**–**9**, **12**) showed good concentration-dependent inhibition on 6-CF uptake mediated by OAT3 with IC_50_ ≤ 10 *µ*M. Among these compounds, 2′-methoxyflavone (**3**) was the most potent inhibitor of OAT1, with IC_50_ value of 2.9 *µ*M, while 3′-hydroxy-4′,5′-dimethoxyflavone (**6**) was of comparable potency with an IC_50_ of 3.2 *µ*M. Comparing **2** with **3** and **10**, the inhibitory activity on OAT1 increased with the presence of methoxy group and decreased with the presence of hydroxyl group at the 2′ position in the B-ring. Additional B ring substitution appeared to reduce potency. Compound **12**, 5,6,2′-trimethoxyflavone, was the most potent inhibitor of OAT3, with an IC_50_ of 2.9 *µ*M. The parent molecule, unsubstituted flavone (**2**) was also a potent inhibitor on OAT3, with an IC_50_ of 4.6 *µ*M. Comparing **2** with **3** and **10**, the addition of a methoxy or hydroxy group at the 2′ position in the B-ring reduced the inhibitory activity on OAT3. Again, additional substitution on the B ring appeared to reduce potency.

Inhibitory activity on OAT1/3 of these flavones is highly dependent on the number and nature of the substituents attached to the flavone ring system, as well as the substitution pattern. In our previous study, 2′-hydroxy-6,7,8-trimethoxyisoflavone and 7-methoxyflavone showed inhibitory activity against OAT1 with IC_50_ values of 9.1 and 3.6 *µ*M, respectively, and 7,2′-dihydroxy-6,8-dimethoxyisoflavone, 2′-hydroxy-6,7,8-trimethoxyisoflavone, 6,2′-dihydroxy-7,8-dimethoxyisoflavone, and 7-methoxyflavone showed moderate inhibitory activity against OAT3 with IC_50_ values of 5.6, 8.7, 17.9, and 5.8 *µ*M, respectively^28^. Morin and luteolin were reported to be potent OAT1 inhibitors (IC_50_ values of <0.3 and 0.47 *µ*M, respectively) in a para-aminohippuric acid (PAH) uptake assay in LLC-PK1 cells, while the tested flavonoid glycosides had weak inhibitory effects on OAT1^29^. Apigenin has been reported to inhibit OAT1 (IC_50_ value of 0.737 *µ*M) on the uptake of acyclovir in MDCK cells^30^. Wogonin has been reported to inhibit OAT1 (IC_50_ value of 5.4 *µ*M), and baicalein and wogonin have been reported to inhibit OAT3 (IC_50_ values of 2.4 and 1.3 *µ*M, respectively) in an assay similar to that used in our study^31^. In addition, flavone was identified by UPLC-ESI-MS/MS as the one main active component of *Gnaphalium pennsylvanicum* extract which showed activity in reducing serum uric acid through OAT1^32^. We have also recently reported the OAT3 inhibitory activity of the more complex dimeric biflavonoids, amentoflavone, cupressuflavone and podocarpusflavone A from *Juniperus oblonga*^33^, with IC_50_ values of 2.0 *μ*M, > 50 *μ*M and 3.8 *μ*M, respectively. Orally administered amentoflavone markedly altered the pharmacokinetic parameters of furosemide, a substrate of Oat3, in rats. Comparison of potency data among these studies is complicated by the use of the number of different bioassays commonly used in this area.

The unusual substitution patterns seen in compounds **1**–**12** may help to define the flavone chemotype as a pharmacophore for OAT inhibitors, providing the basis for future structure activity relationship studies. Identification of these active compounds may also help establish the chemical basis for use of this herb in the treatment of kidney disease.

Evaluation of these flavones as antifungal agents against *C. rugosa* showed that compounds **7**, **8**, and **12** possessed marked antifungal activity against this organism with MIC values of 0.4 *µ*M, 1.2 *µ*M, and 2.0 *µ*M, respectively. These three flavones share the common structural features of methoxylation at positions 5 and 6 of the A ring and position 2′ of the B ring. While **11** which is inactive also shares these features, it is also methoxylated at C-8, suggesting that substitution at C-8 greatly reduces or eliminates activity. Although flavone (**2**) shows minimal activity against *C. rugosa* (MIC = 500* µ*M), none of the other compounds lacking the methoxylation at 5, 6, and 2′ showed any antifungal activity. Most known flavones are oxygenated at positions 5 and 7 on the A ring but are not noted for having antifungal activity, suggesting that the unusual 5,6-disubstitution pattern found in our isolates may be required for this activity. Further study is needed to define optimal substitution on the B ring and to explore the effect of substituents other than methoxy groups on the activity of these compounds. The pressing need for new antifungal chemotypes, especially for non-*albicans Candida* infections including the emerging “superbug”, *C. auris*, highlights the importance of the discovery and potential development of a new pharmacophore such as this.

## Methods

### General experimental procedures

UV spectra were recorded on a HITACHI U-3900 spectrophotometer (Tokyo, Japan). UPLC-QQQ-MS data were recorded on Agilent 1260–6420 series spectrometer (Agilent Technologies, Santa Clara, CA, USA). HRESIMS spectra were performed on a MicroTOF-QII mass spectrometer (Bruker Daltonics Inc, Billerica, MA, USA) in positive or negative ion mode. All 1D and 2D NMR spectra were acquired using Avance III spectrometers (Bruker-Biospin, Billerica MA, USA) operating at 400 MHz or 600 MHz (^1^H) and 100 MHz or 150 MHz (^13^C) at ambient temperature. Residual solvent resonances were used as internal standard. Chemical shifts are expressed in PPM. Open column chromatography was carried out using Sephadex LH-20 (GE Healthcare Bio-Science AB, Uppsala, Sweden). Thin-layer chromatography (TLC) was performed on precoated layers of Silica gel GF254 (Qingdao Marine Chemical, Inc., Qingdao, China). Flash chromatography was performed on a CombiFlash Rf + instrument (Teledyne-ISCO, Lincoln, NE, USA) with prepacked silica flash column (Santai Technologies, Inc., Jiangsu, China) in various sizes. Analytical and semi-preparative HPLC were performed on an Agilent 1260VL quad gradient system (G1311C pump, G1329B autosampler, G1316A thermostatted column compartment and G1315D photodiode array detector, Agilent Technologies, Santa Clara CA, USA), using either a Thermo Hypersil Gold C_18_ (150 × 2.1 mm, 3 *µ*m), an Agilent Poroshell 120 EC-C_18_ analytical chromatographic column (150 × 2.1 mm, 2.7 *µ*m), or a Thermo Hypersil Gold C_18_ semipreparative chromatographic column (250 × 10 mm, 5 *µ*m). Preparative HPLC separations were performed on an Agilent 1260VL quad gradient system (G1311C pump, and G1315D photodiode array detector) equipped with a Rheodyne 7725i manual injection valve (IDEX Health & Science, Middleboro MA, USA) and Shimadzu CTO-20A column oven (Shimadzu Scientific Instruments, Kyoto, Japan), using a Thermo Hypersil Gold C_18_ chromatographic column (250 × 21.2 mm, 5 *µ*m). The determination of fluorescence was performed on an Infinite M200 plate reader (Tecan, Mannedorf, Switzerland) with excitation and emission wavelengths at 485 and 528 nm, respectively. Microbial culture density was measured on a MAPADA UV-1800 spectrophotometer (MAPADA, Shanghai, China). Incubation and biological operation were performed in electro-thermal constant temperature incubators and super clean bench (Shanghai Zhicheng Analysis Instrument Manufacturing Co., Ltd., Shanghai, China), respectively. 96 well cell culture plates (Costar 3599) was purchased from Corning (NY, USA). Optical density reader for microorganism was recorded on a Multiskan plate reader (Thermo Fisher Scientific Oy Microplate Instrumentation, Vantaa, Finland).

All solvents used were HPLC grade (Tianjin Concord Technologies; Tianjin Guangfu Technology Development Co., Ltd., Tianjin, China). 6-Carboxyfluorescein (6-CF) was purchased from Aladdin (Shanghai, China). Probenecid and hygromycin B were purchased from Solarbio (Beijing, China). Poly-D-Lysine was purchased from Sigma-Aldrich Chemical Co. (St. Louis, MO, USA). Dulbecco’s modified Eagle’s medium (DMEM), trypsin and fetal bovine serum (FBS) were purchased from Gibco (Gaithersburg, MD, USA). Bicinchoninic acid (BCA) protein assay kit was purchased from Cwbio (Beijing, China).

All antibiotics, such as penicillin, ampicilin, ciprofloxacin, cephalexin, vancomycin amphotericin B, and fluconazole, were purchased from Sigma-Aldrich Chemical Co. (St. Louis, MO, USA). Five media, such as nutrient ager (AOBOX, Beijing, China), brain heart infusion broth and potato dextrose broth (Solarbio, Beijing, China), YM medium (Hopebio, Qingdao, China), and agar (Guangfu-chem, Tianjin, China), were used. Microorganisms were obtained from the American Type Culture Collection (ATCC, Manassas, Virginia, USA).

### Plant material

The whole plants *Primula macrocalyx* Bunge (Primulaceae) used in this study were collected in Armenia, Vayots Dzor Province, near Yeghegnadzor, between Aghnjadzor and the Selim Pass (Alt: 2100 m; GPS: 39.9300 N, 45.2425 E) in May 2006. Voucher specimens (G. Fayvush 10–2006) have been deposited in the herbaria of the Institute of Botany, Armenian National Academy of Sciences (ERE), and the New York Botanical Garden (NY).

### Extraction and isolation

Plant samples comprising the whole plant of *P. macrocalyx* were freed of extraneous matter, air dried in the shade and then ground to a coarse powder. A 1 kg portion of this sample was extracted three times with methanol (6 L, 1 day each) at room temperature to give the methanol extract (108 g) on removal of the solvent *in vacuo*.

The sample was dissolved in methanol and water (300 mL, 9:1 v/v), and extracted with *n*-hexane (300 mL × 3) to get an *n*-hexane fraction. The residual methanolic phase was freed of methanol *in vacuo*, suspended in water (300 mL), extracted with dichloromethane (300 mL × 3) followed by water-saturated *n*-butanol (300 mL × 3), to obtain a dichloromethane fraction, an *n*-butanol fraction and an aqueous fraction. The four fractions were each freed of solvents *in vacuo* and subjected to preliminary evaluation of inhibitory activity on OAT1 and OAT3 and antimicrobial activities against sixteen microorganisms. The dichloromethane fraction elicited strong inhibition of OAT1 and OAT3 and strong antifungal activity against *C. rugosa*, *in vitro*.

The active dichloromethane soluble fraction (3.5 g) was then fractionated by chromatography on silica gel (40–63 *µ*m, 60 Å, 200 g, 204 × 60 mm) eluted with a step-gradient of dichloromethane, ethyl acetate (100:0, 98:2, 96:4, 94:6, 92:8, 90:10, 70:30, 50:50, 0:100, 780 mL each) to yield fractions 1–30 (each 260 mL). Thirty fractions from this separation were evaluated for bioactivity on OAT1 and OAT3 and antifungal activity against *C. rugosa*. HPLC comparison of the active fractions using an Agilent Poroshell 120 EC-C_18_ column (150 × 2.1 mm, 2.7 *μ*m) eluted with ACN: H_2_O (1:1) containing 0.1% formic acid at 0.2 mL/min at 40 °C (fractions 1–15) or ACN: H_2_O (2:3) containing 0.1% formic acid at 0.2 mL/min at 40 °C (fractions 16–30) allowed combination to form 7 pools, Fractions A-G.

Active fractions 4–5 from the silica gel column were pooled to form Fraction A, which consisted of one major peak (**1**) with a retention time of 7.45 min. Fraction A (216 mg) was purified by recrystallization using dichloromethane-methanol mixtures (7:3, 10 mL) three times to afford compound **1** (10 mg).

Active fractions 6–7 from the silica gel column were pooled to form Fraction B, which consisted of two main peaks with retention times of 6.78 min (**2**) and 7.25 min (**3**). Fraction B (372 mg) was chromatographed on a flash silica column (40–63 *µ*m, 60 Å, 4 g, 106 × 12 mm) eluted with a linear gradient of *n*-hexane and dichloromethane (75:25–50:50, 240 mL) and purified further on Sephadex LH-20 (12 g, 305 × 17 mm) eluted with dichloromethane: methanol (50:50, 200 mL) to yield compound **3** (12 mg) and **2** (9 mg).

Active fractions 12 from the silica gel column formed Fraction C, which consisted of one main peak with a retention time of 7.69 min (**4**). Fraction C (403 mg) was purified by gel filtration on Sephadex LH-20 (12 g, 305 × 17 mm) eluted with dichloromethane: methanol (50:50, 200 mL), then methanol (100 mL), affording compound **4** (34 mg).

Active fractions 17–20 from the silica gel column were pooled to form Fraction D, which consisted of two main peaks with retention times of 10.13 min (**7**) and 12.68 min (**12**). Active fraction 21 formed Fraction E with two main components eluting at 5.06 min (**5**) and 6.22 min (**6**). Fractions D and E were combined (780 mg) and separated by repeated preparative HPLC (Thermo Hypersil Gold C_18_, 250 × 21.2 mm, 5 *µ*m) eluted with a gradient of 22–28% aqueous MeCN containing 0.1% formic acid (1200 mL, at 10 mL/min, 40 °C) and further purified by semi-preparative HPLC (Thermo Hypersil Gold C_18_, 250 × 10 mm, 5* µ*m, 4 mL/min; 40 °C) eluted isocratically with 22% (400 mL) or 24% (480 mL) aqueous MeCN containing 0.1% formic acid to afford compound **5** (17 mg), **6** (20 mg), **7** (10 mg) and **12** (15 mg).

Active fractions 22–24 from the silica gel column were pooled to form Fraction F, which consisted of four main peaks with retention times of 5.63 min (**9**), 7.16 min (**10**), 8.84 (**11**), and 10.18 min (**8**). Fraction F (330 mg) was subjected to preparative HPLC (Thermo Hypersil Gold C_18_, 250 × 21.2 mm, 5 *µ*m, 8 mL/min, 40 °C) eluted with 22–25% aqueous MeCN with 0.1% formic acid (360 mL) to give **11** (5 mg) and **8** (12 mg), and purified further by semi-preparative HPLC (Thermo Hypersil Gold C_18_, 250 × 10 mm, 5* µ*m, 4 mL/min; 40 °C) eluted with 22% aqueous MeCN with 0.1% formic acid (240 mL) to afford **9** (8 mg) and **10** (17 mg).

Active fraction 25 from the silica gel column afforded additional quantities of **8**.

Antifungal activity was limited to fractions 17–25 from the silica gel column (Fractions D-G).

2′*-methoxy-5*′*-hydroxyflavone (****5****)*. Yellow amorphous powder; UV (MeOH) *λ*_*max*_ (log *ε*) 212 *sh* (3.90), 245 (3.75), 295 (3.63), 352 (3.39) nm; HRESIMS m/z 269.0805 [M + H]^+^ (calcd. for C_16_H_13_O_4_ 269.0808), m/z 291.0625 [M + Na]^+^ (calcd. for C_16_H_12_O_4_Na 291.0633); ^1^H NMR (DMSO-*d*_6_, 600 MHz) and ^13^C NMR (DMSO-*d*_6_, 150 MHz) data, see Table 1.

*5,7,8,2*′*,6*′*-pentamethoxyflavone (****11****)*. Pale yellow amorphous powder; UV (MeOH) *λ*_*max*_ (log *ε*) 227 (4.05), 261 (3.91), 335 (3.39) nm; HRESIMS m/z 373.1275 [M + H]^+^ (calcd. for C_20_H_21_O_7_ 373.1282); ^1^H NMR (Acetone-*d*_6_, DMSO-*d*_6_, and CDCl_3_, 600 MHz) and ^13^C NMR (Acetone-*d*_6_, DMSO-*d*_6_ and CDCl_3_, 150 MHz) data, see Table 1.

### Cell culture

Human embryonic kidney 293 (HEK293) cell lines stably overexpressing OAT1 and OAT3 were established and identified as previously described^14^. Cell lines stably expressing HEK-OAT1 and HEK-OAT3 were obtained by hygromycin B (75 *µ*g/mL) selection, and further characterized by both mRNA expression of transporters and their uptake of 6-CF, a fluorescent substrate for both OAT1 and OAT3^34^. The cells were cultivated in DMEM supplemented with 10% FBS, 1% penicillin/streptomycin, and 75 *µ*g/mL hygromycin B at 37 °C with 5% CO_2_.

### Uptake assay

The interactions of 12 flavones with the uptake of 6-CF in HEK-OAT1 and HEK-OAT3 cells were evaluated. This cell uptake assay was performed as previously described^14,35^. A density of 5 × 10^4^ cells were seeded per well in 96-well culture plates precoated with poly-D-lysine. Approximately 85% confluency of cells was obtained after growing 24 h. The cells were washed twice and preincubated for 5 min with preheated (37 °C) uptake buffer (135 mM NaCl, 5 mM KCl, 2.5 mM CaCl_2_, 1.2 mM MgCl_2_, 0.8 mM MgSO_4_, 28 mM glucose, and 13 mM Hepes, pH 7.2) for the following uptake experiments. The uptake buffer containing 4 *µ*M 6-CF in the presence or absence of test compounds and probenecid (a classic inhibitor of OAT1 and OAT3, used as a positive control) was incubated for 5 min to allow uptake. Uptake was terminated by adding 100 *µ*L ice-cold uptake buffer, and quickly washing the cells in each well three times with ice-cold phosphate-buffered saline (PBS). The cells were lysed with 100 *µ*L of 20 mM Tris-HCl containing 0.2% TritonX-100. A 50 *µ*L aliquot of lysate was used to quantify fluorescence using a Tecan Infinite M200 plate reader with excitation and emission wavelengths at 485 and 528 nm, respectively. The protein content of the cell lysate was quantified using a BCA Protein Assay Kit. The intensity of fluorescence was standardized against total protein content, and measured in triplicate. The stock solutions of tested compounds were dissolved in DMSO with a final concentration of 50 mM and dilutions were made using uptake buffer. In our initial screening, the inhibition of the 12 compounds was evaluated at a concentration of 50 *µ*M on OAT1 and OAT3. As shown, the majority of compounds showed marked inhibitory effects on OAT1 and OAT3. An inhibitor is defined as a compound that results in > 70% inhibition of 6-CF uptake. Concentration-dependent inhibition experiments were carried out on all selected inhibitors to determine IC_50_ (50% inhibitory concentration) values on OAT1 and OAT3.

### Statistical analysis

IC_50_ values were summarized in Table 2 were estimated by non-linear regression analysis and expressed as mean ± standard error of mean. Statistical analysis was performed using GraphPad Prism version 7.0. For the uptake experiments, data were analyzed with a two-tailed unpaired Student’s t-test.

### Well diffusion antimicrobial assay

Antimicrobial activities of four crude fractions (*n*-hexane fraction, dichloromethane fraction, *n*-butanol fraction and aqueous fraction) against sixteen bacteria and fungi were performed based on the method reported previously^36,37^ with slight modification using agar plates.

Briefly, nutrient ager (NA), brain heart infusion agar (BHIA) and potato dextrose agar (PDA), YM agar (YMA) were mixed in appropriate proportions with ultrapure water and autoclaved for 20 min at 121 °C. Approximately 15 mL aliquots of each medium were dispensed into petri plates and allowed to solidify, then incubated for 24 h to ensure sterility prior to use. Microbial suspensions (0.5 mL) at a density of 5 × 10^6^ cfu/mL were inoculated onto the sterile media and allowed to stand for 10–20 min before wells were cut into the agar using a sterile pipet tip. A 10 *µ*L aliquot of a DMSO solution of plant extracts (1 mg/mL) or fractions (2, 0.2, 0.02 mg/mL) were pipetted into each well. Similarly, 10 *µ*L portions of DMSO and a solution of an appropriate antibiotic standard were applied to each plate as negative and positive controls, respectively. All treated plates were incubated for 24 h in incubators at appropriate temperatures. Zones of inhibition were measured in millimeters. The assays were performed in triplicate. Proper media, temperature and antibiotic standards for each microorganism are shown in Table 3.

**Table 3:** Microorganisms, media, incubation temperature, and antibiotic controls.

ATCC No.	Species	Media	Temp/°C	Antibiotic
**Bacteria**				
ATCC 12600	*Staphylococcus aureus*	Nutrient Agar	37	Penicillin
ATCC 14990	*Staphylococcus epidermis*	Nutrient Agar	37	Ampicillin
ATCC 25175	*Streptococcus mutans*	Brain Heart Infussion Agar	37	Ampicillin
ATCC 13525	*Pseudomonas fluorescens*	Nutrient Agar	26	Ciprofloxacin
ATCC 8043	*Enterococcus hirae*	Brain Heart Infussion Agar	37	Ampicillin/Cephalexin
ATCC 25238	*Moraxella (Branhamella) catarrhalis*	Brain Heart Infussion Agar	37	Cephalexin
ATCC 15692	*Pseudomonas aeruginosa*	Nutrient Agar	37	Ciprofloxacin
ATCC 6633	*Bacillus subtilis subsp. spizizenii*	Brain Heart Infusion Agar	30	Vancomycin
**Fungi**				
ATCC 76615	*Candida albicans*	YM Medium Agar	24	Amphotericin B
ATCC 16888	*Aspergillus niger*	Potato Dextrose Agar	30	Amphotericin B
ATCC 2601	*Saccharomyces kudriavzevii*	YM Medium Agar	30	Fluconazole
ATCC 10106	*Penicillium chrysogenum*	Potato Dextrose Agar	25	Amphotericin B
ATCC 22019	*Candida parapsilosis*	YM Medium Agar	24	Amphotericin B
ATCC 10571	*Candida rugosa*	YM Medium Agar/Broth	25	Amphotericin B
ATCC 750	*Candida tropicalis*	YM Medium Agar	25	Amphotericin B
ATCC 34103	*Rhizopus stolonifer*	Potato Dextrose Agar	25	Amphotericin B

### Broth microdilution antifungal assay

Antifungal activity against *C. rugosa* (ATCC 10571) was measured as previously described using a broth microdilution method^36–38^ with minor modifications. The assay was performed in sterile 96-well microtiter plates. Briefly, YM broth was prepared and autoclaved for 20 min at 121 °C, then allowed to cool to room temperature (25 °C). *C. rugosa* was dispersed in YM broth to an optical density of 1 UA at 600 nm (approximately 1 × 10^7^ cfu/mL) using uninoculated broth as the blank. A 198 *µ*L aliquot of the resulting fungal suspension was dispensed into each well of a 96-well plate. Stock solutions of the twelve compounds were prepared in DMSO at a concentration of 50 mM. The stock solutions were diluted to a range of final concentrations (500–0.14 *µ*M) in YM broth such that the final DMSO concentration was no higher than 1%, v/v. A 2 *µ*L aliquot of the resulting test samples was transferred to each well of the plate. Plates were incubated at 25 °C for 24 h prior to measurement of the optical density of each well at 600 nm. Minimal inhibitory concentrations (MICs), defined as the lowest concentration required to visibly inhibit the fungal growth compared to the untreated control, were measured in triplicate. Amphotericin B was used as positive control.

## Supplementary information

Unusual Flavones from Primula macrocalyx as Inhibitors of OAT1 and OAT3 and as Antifungal Agents against Candida rugosa

## Data Availability

The datasets generated in this study are available from the corresponding author on reasonable request.
