# Supplementary material for: Unusual Flavones from Primula macrocalyx as Inhibitors of OAT1 and OAT3 and as Antifungal Agents against Candida rugosa
Source: Sci Rep. 2019 Jun 25;9:9230. doi: 10.1038/s41598-019-45728-5 (PMC6592895; doi:10.1038/s41598-019-45728-5)
Supplement: Supplementary file 1 — Unusual Flavones from Primula macrocalyx as Inhibitors of OAT1 and OAT3 and as Antifungal Agents against Candida rugosa [file 41598_2019_45728_MOESM1_ESM.pdf]

Unusual Flavones from *Primula macrocalyx* as Inhibitors of OAT1 and OAT3 and as Antifungal Agents against *Candida rugosa*

Xue Li[1], Xue Wang[1], Caiyu Li[1], Manana Khutsishvili[2], George Fayvush[3], Daniel Atha[4], Youcai Zhang[1], Robert P. Borris[1*]

[1]School of Pharmaceutical Science and Technology, Health Sciences Platform, Tianjin University, 92 Weijin Rd., Nankai District, Tianjin, 300072, CHINA.

[2]National Herbarium of Georgia, Ilia State University, Tbilisi, GEORGIA.

[3]Institute of Botany, Armenian National Academy of Sciences, Yerevan, ARMENIA.

[4]New York Botanical Garden, Bronx, New York, USA.

*Corresponding author: Tel +86-18302242039, rborris@tju.edu.cn

Contents

Figure S1: HRESIMS spectrum of compound 5 2
Figure S2: ^{1}H NMR spectrum of compound 5 (DMSO-d_6, 600MHz) 3
Figure S3: ^{13}C NMR spectrum of compound 5 (DMSO-d_6, 150MHz) 4
Figure S4: HSQC spectrum of compound 5 5
Figure S5: HMBC spectrum of compound 5 6
Figure S6: ^{1}H-^{1}H COSY spectrum of compound 5 7
Figure S7: NOESY spectrum of compound 5 8
Figure S8: UV-Vis spectrum of compound 5 9
Figure S9: HRESIMS spectrum of compound 11 10
Figure S10: ^{1}H NMR spectrum of compound 11 (Acetone-d_6, 600MHz) 11
Figure S11: ^{13}C NMR spectrum of compound 11 (Acetone-d_6, 150MHz) 12
Figure S12: HSQC spectrum of compound 11 13
Figure S13: HMBC spectrum of compound 11 14
Figure S14: ^{1}H-^{1}H COSY spectrum of compound 11 15
Figure S15: NOESY spectrum of compound 11 16
Figure S16: UV-Vis spectrum of compound 11 17
Figures S17: 1D and 2D NMR spectra of compound 1 ($CDCl_3$, 600 MHz) 18
Figures S18: 1D and 2D NMR spectra of compound 2 ($CDCl_3$, 600MHz) 21
Figures S19: 1D and 2D NMR spectra of compound 3 ($CDCl_3$, 600MHz) 25
Figures S20: 1D and 2D NMR spectra of compound 4 ($CDCl_3$, 600MHz) 28
Figures S21: 1D and 2D NMR spectra of compound 6 (methanol-d_4, 600MHz) 32
Figures S22: 1D and 2D NMR spectra of compound 7 (methanol-d_4, 600MHz) 35
Figures S23: 1D and 2D NMR spectra of compound 8 (methanol-d_4, 600MHz) 39
Figures S24: 1D and 2D NMR spectra of compound 9 (methanol-d_4, 600MHz) 42
Figures S25: 1D and 2D NMR spectra of compound 10 (methanol-d_4, 600MHz) 45
Figures S26: 1D and 2D NMR spectra of compound 12 (methanol-d_4, 600MHz) 49
Table S1 ^{13}C (150 MHz) and ^{1}H (600 MHz) NMR data of Compounds 1-4 (δ in ppm, J in Hz, $CDCl_3$)^a 53
Table S2 ^{13}C (150 MHz) and ^{1}H (600 MHz) NMR data of Compounds 6-9 (δ in ppm, J in Hz, methanol-d_4) 55
Table S3 ^{13}C (150 MHz) and ^{1}H (600 MHz) NMR data of Compounds 10 and 12 (δ in ppm, J in Hz, methanol-d_4) 57

Figure S1: HRESIMS spectrum of compound 5

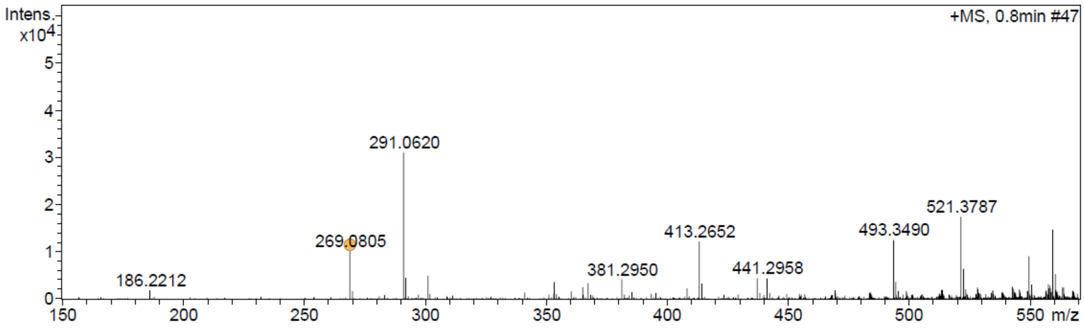

Meas. m/z	#	Ion Formula	m/z	err [ppm]	mSigma	# Sigma	Score	rdb	e¯ Conf	N-Rule
269.0805	1	C16H13O4	269.0808	1.2	7.7	1	100.00	10.5	even	ok

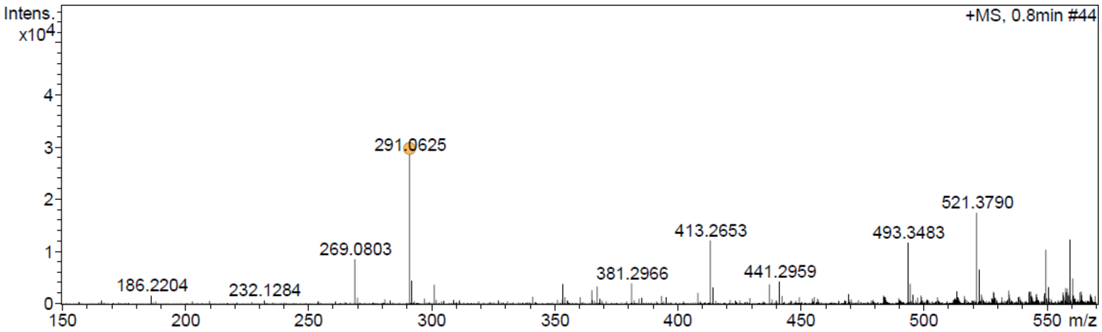

Meas. m/z	#	Ion Formula	m/z	err [ppm]	mSigma	# Sigma	Score	rdb	e¯ Conf	N-Rule

Figure S2: ^{1}H NMR spectrum of compound 5 (DMSO-d_6, 600MHz)

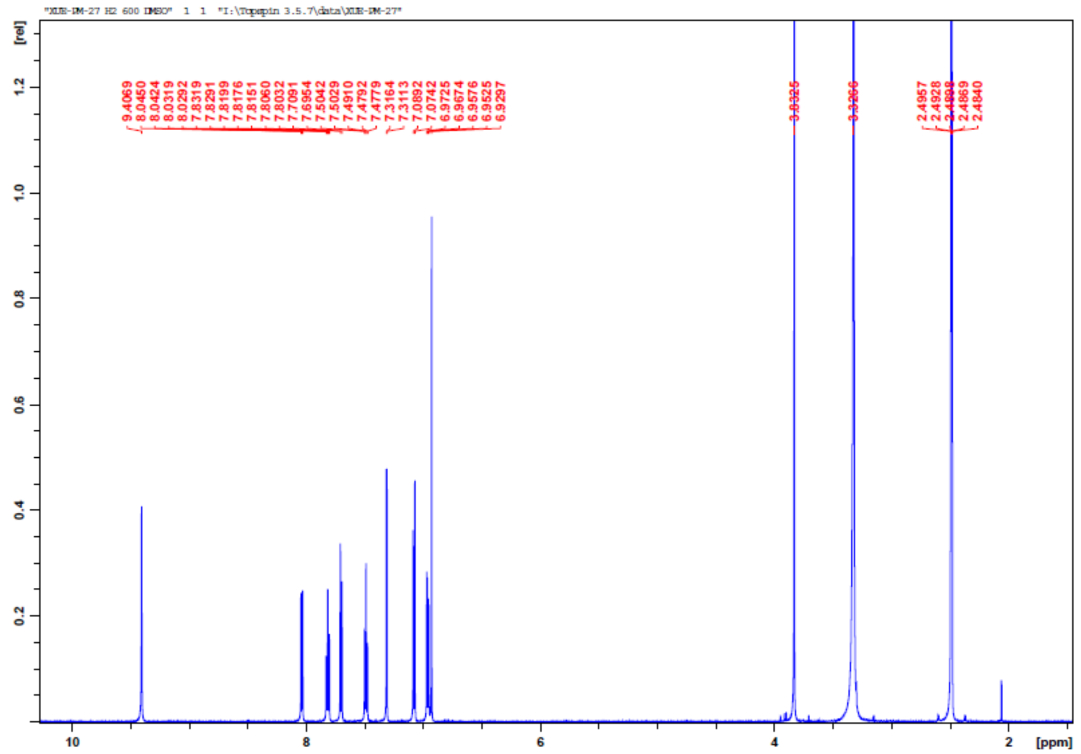

Figure S3: ^{13}C NMR spectrum of compound 5 (DMSO-d_6, 150MHz)

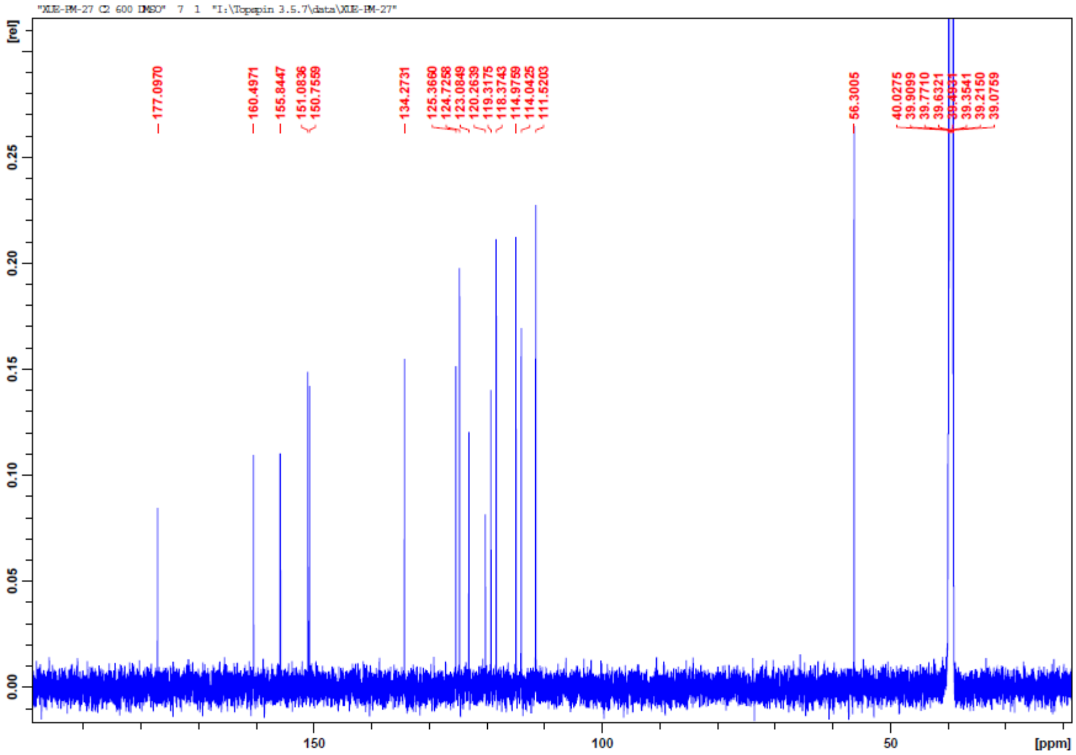

Figure S4: HSQC spectrum of compound 5

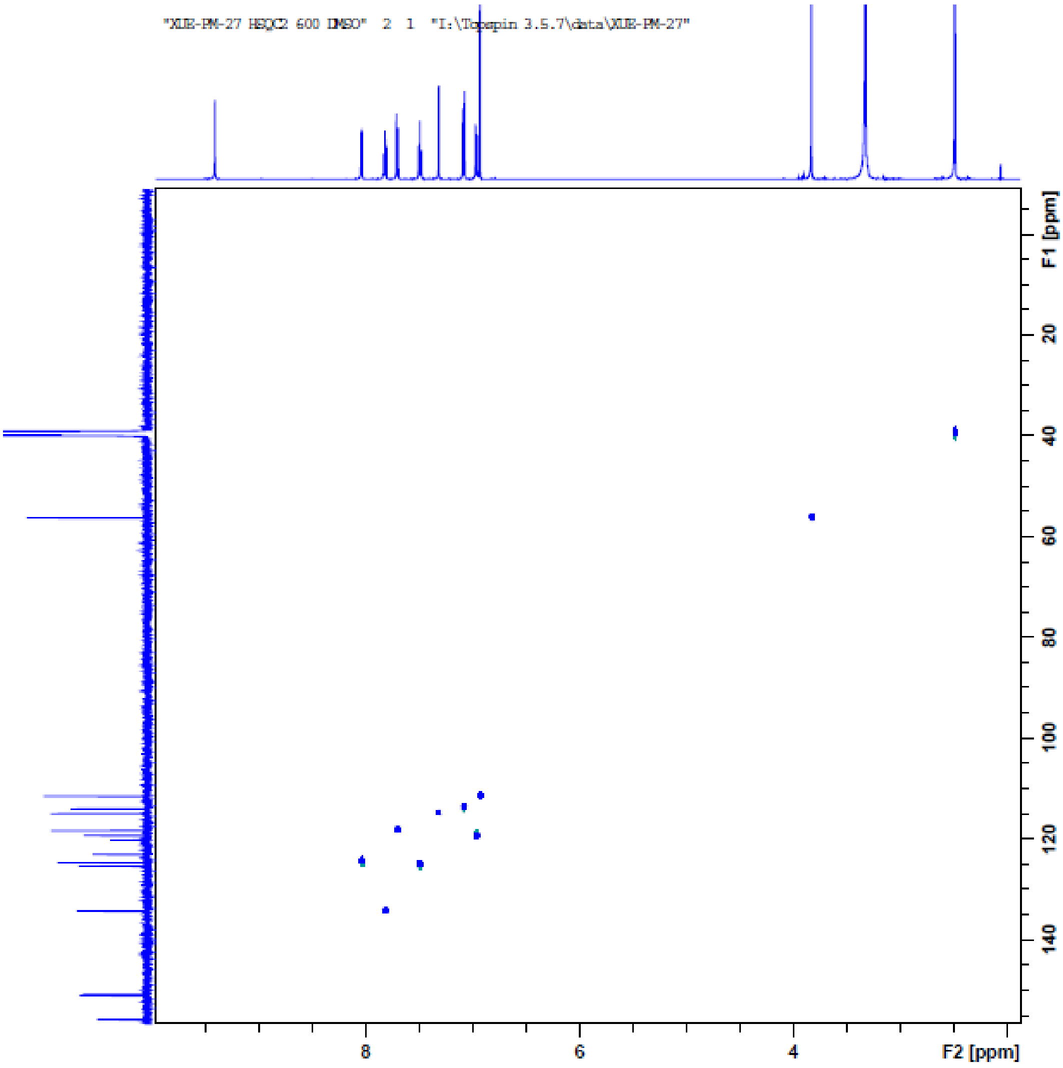

Figure S5: HMBC spectrum of compound 5

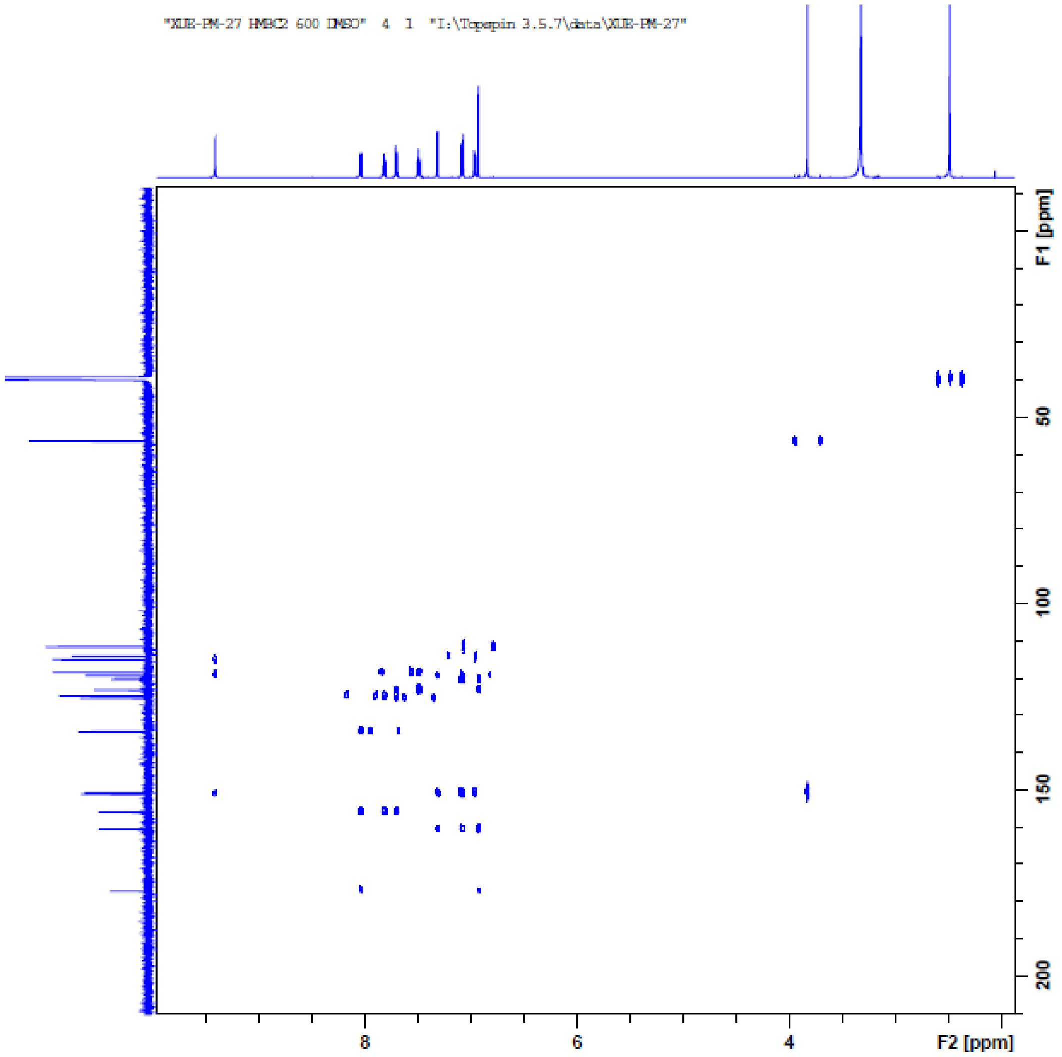

Figure S6: ^{1}H-^{1}H COSY spectrum of compound 5

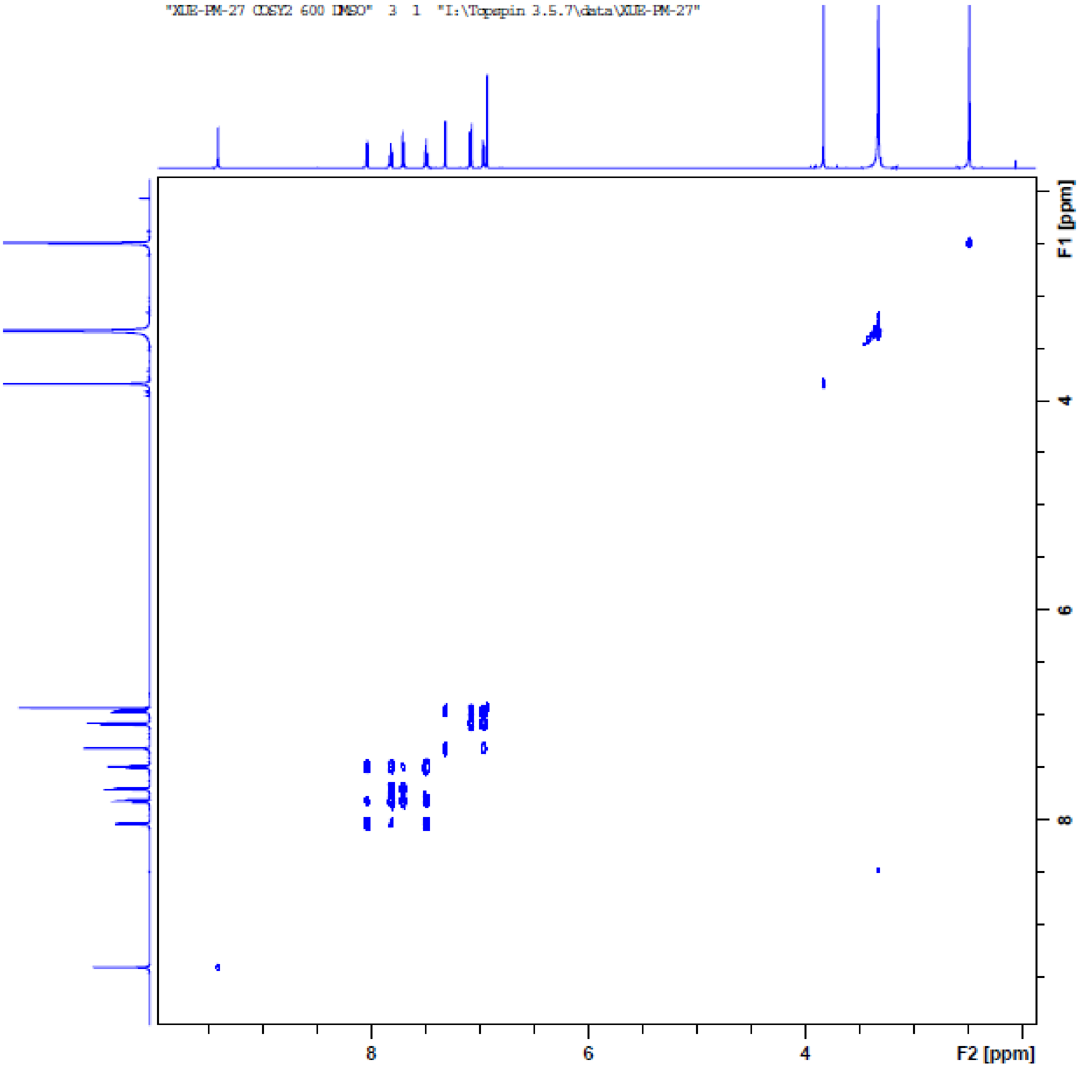

Figure S7: NOESY spectrum of compound 5

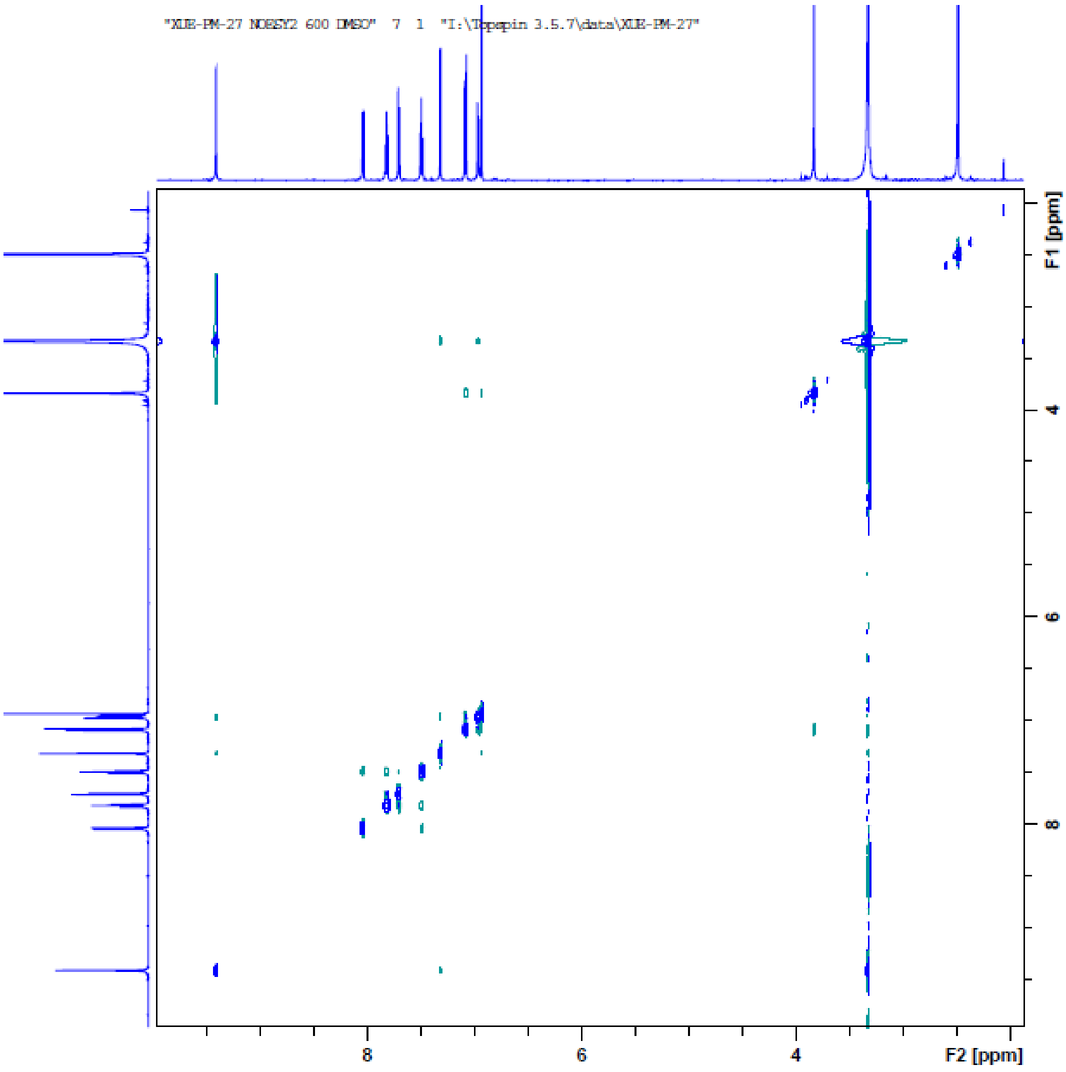

Figure S8: UV-Vis spectrum of compound 5

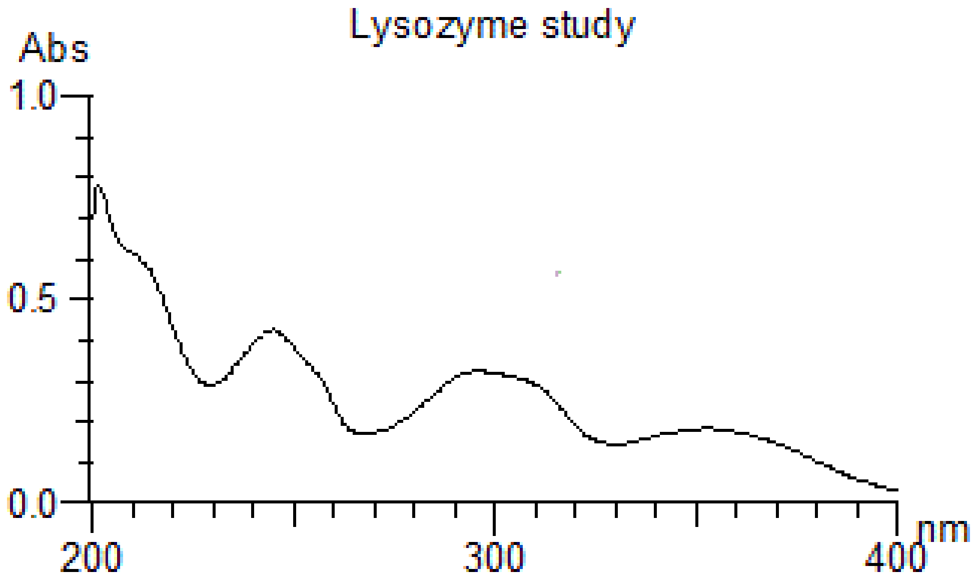

Figure S9: HRESIMS spectrum of compound 11

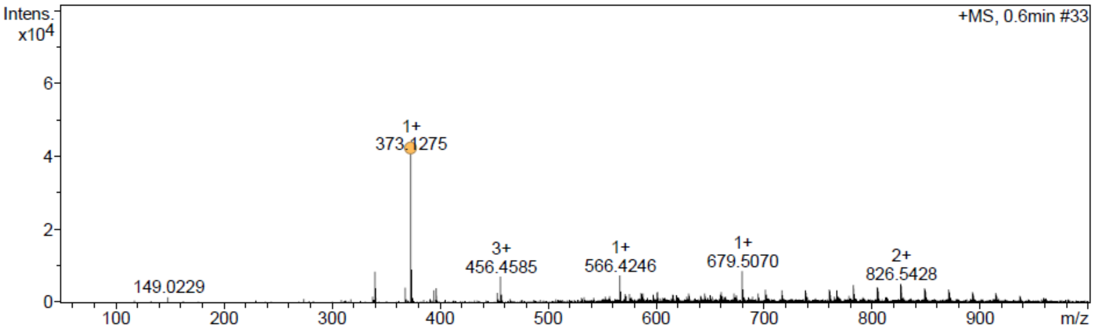

Meas. m/z	#	Ion Formula	Score	m/z	err [mDa]	err [ppm]	mSigma	rdb	e⁻ Conf	N-Rule	Adduct
373.127512	1	C20H21O7	100.00	373.128179	0.7	1.8	4.4	10.5	even	ok	M+H
	2	C15H21N2O9	8.57	373.124157	3.4	9.0	24.1	6.5	even	ok	M+H

Figure S10: ^{1}H NMR spectrum of compound 11 (Acetone-d_6, 600MHz)

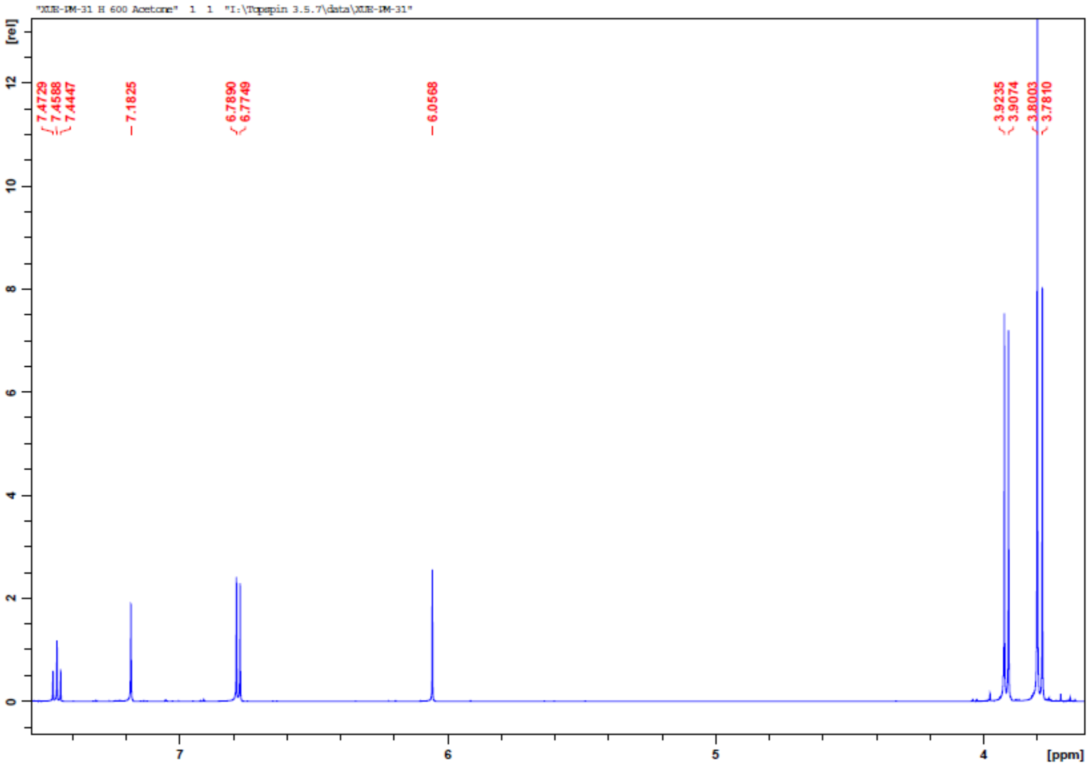

Figure S11: ^{13}C NMR spectrum of compound 11 (Acetone-d_6, 150MHz)

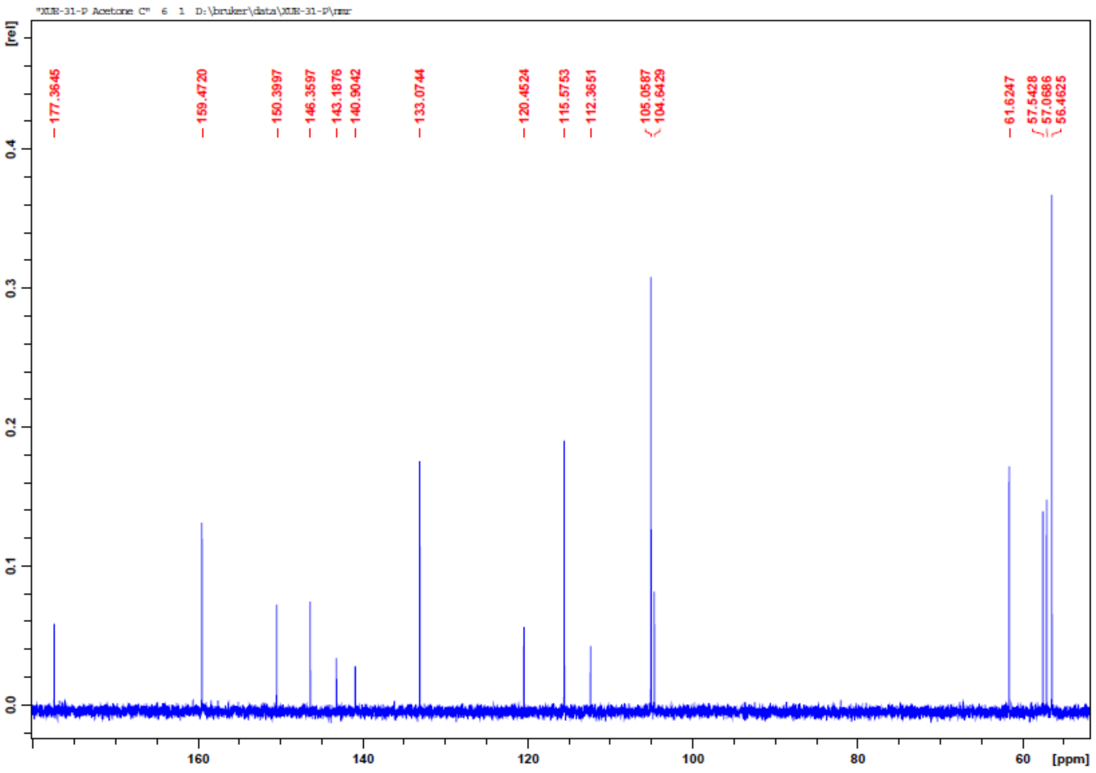

Figure S12: HSQC spectrum of compound 11

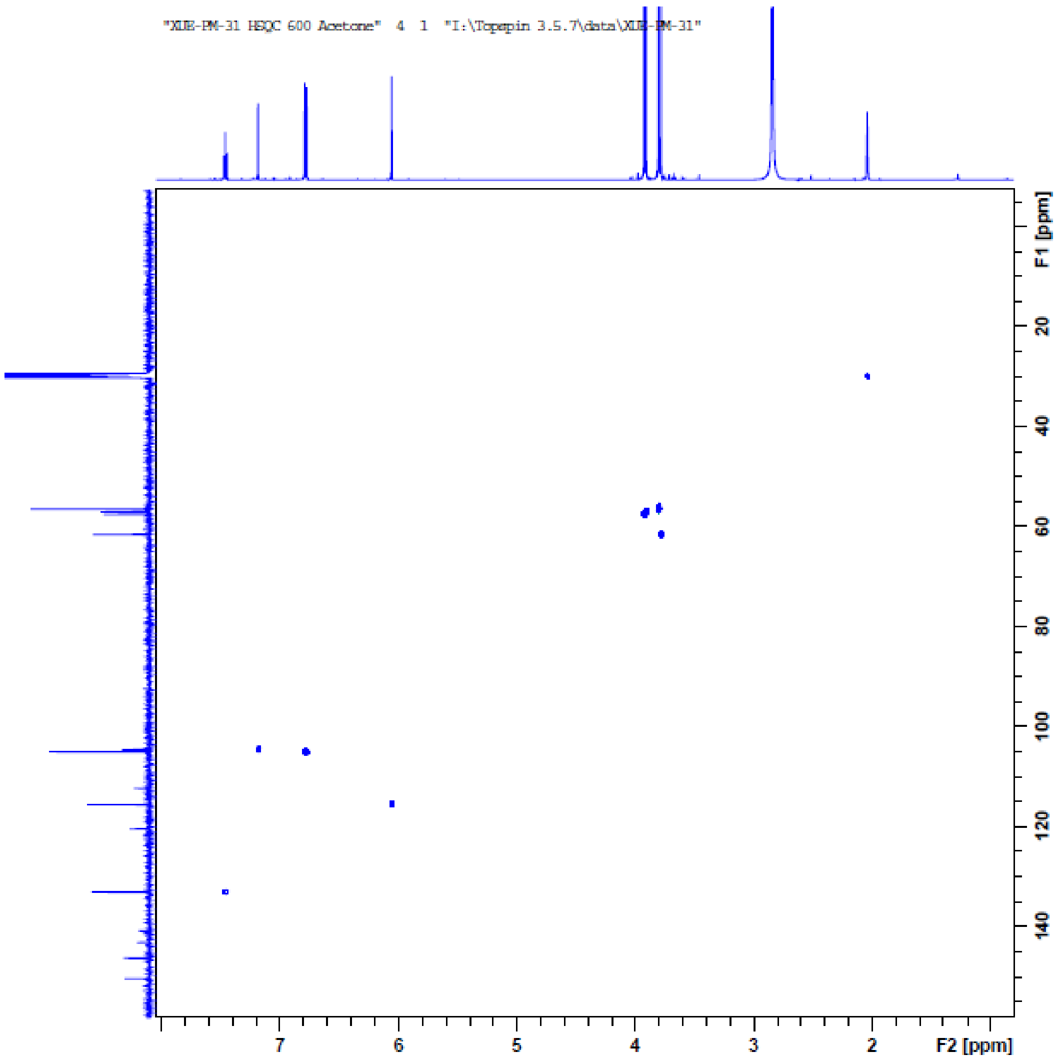

Figure S13: HMBC spectrum of compound 11

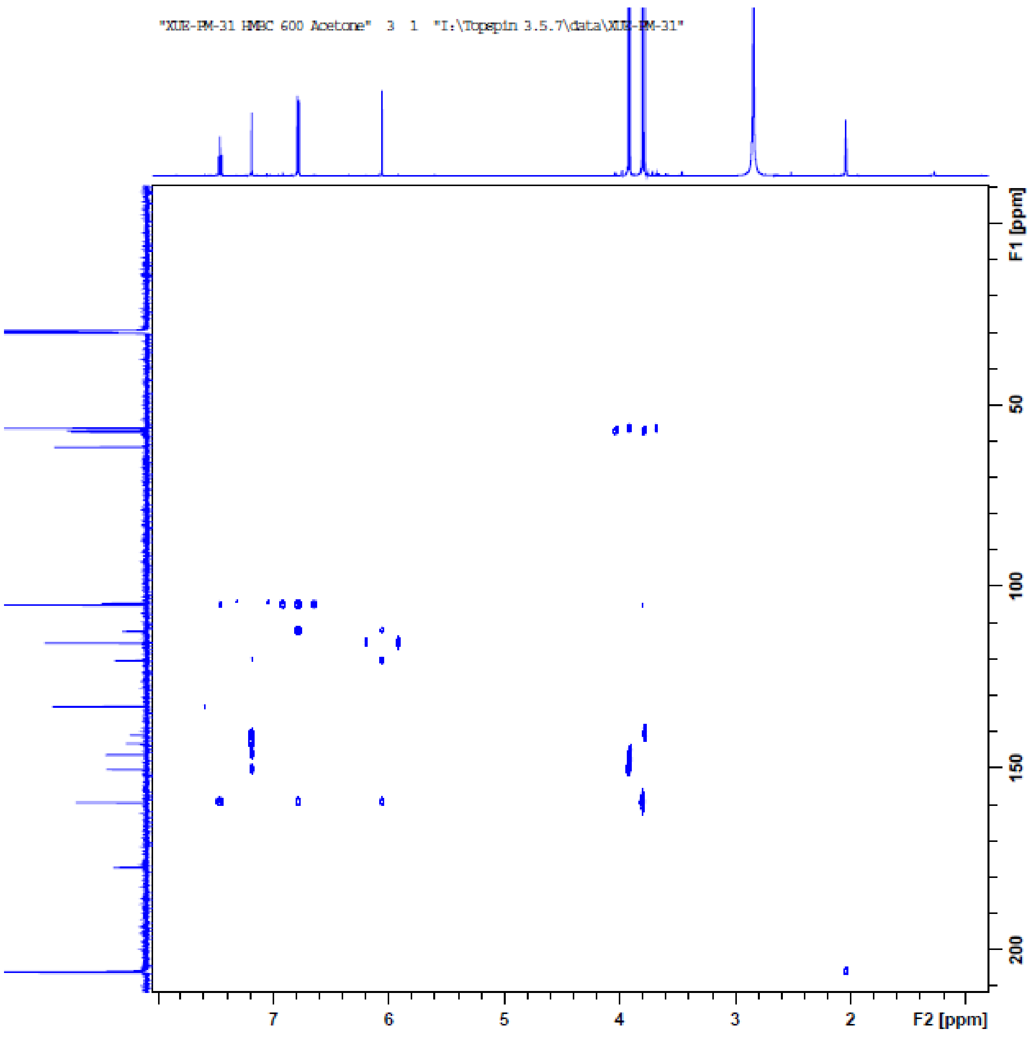

Figure S14: ^{1}H-^{1}H COSY spectrum of compound 11

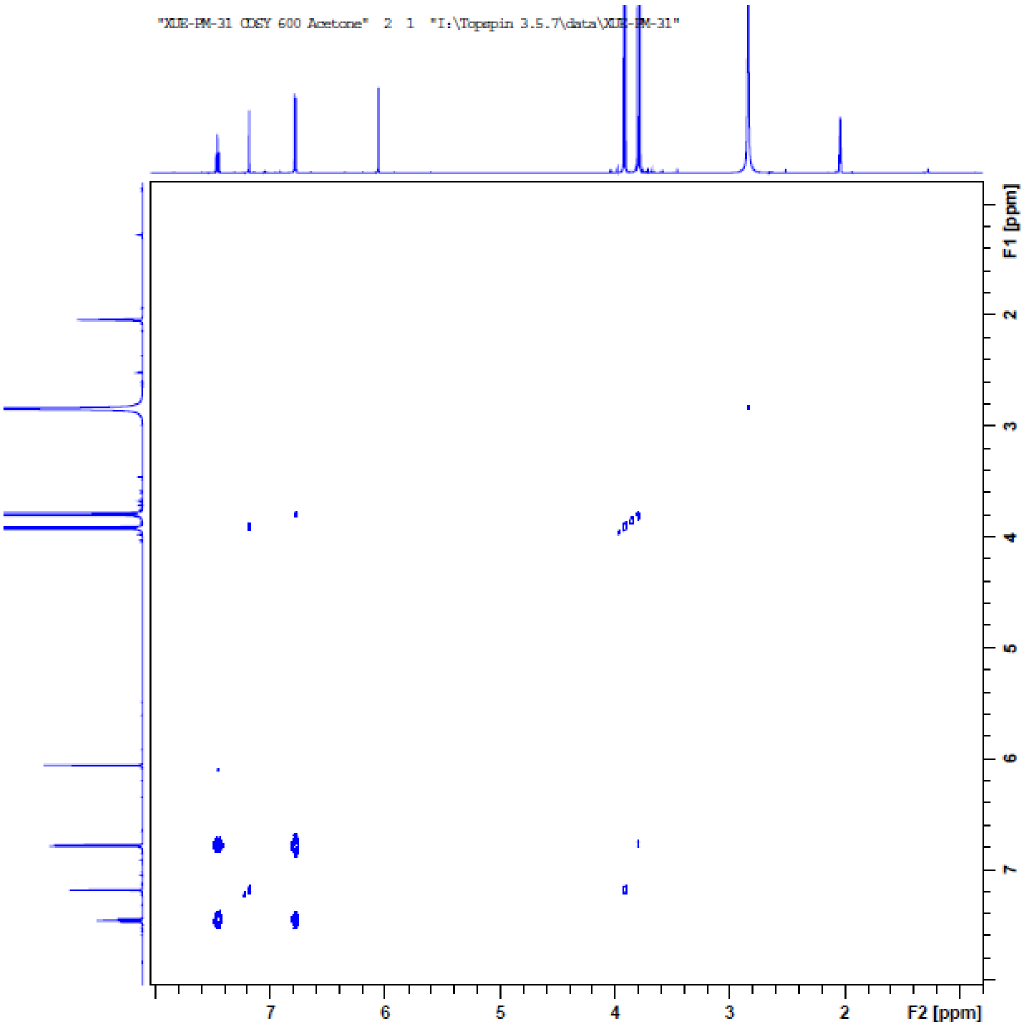

Figure S15: NOESY spectrum of compound 11

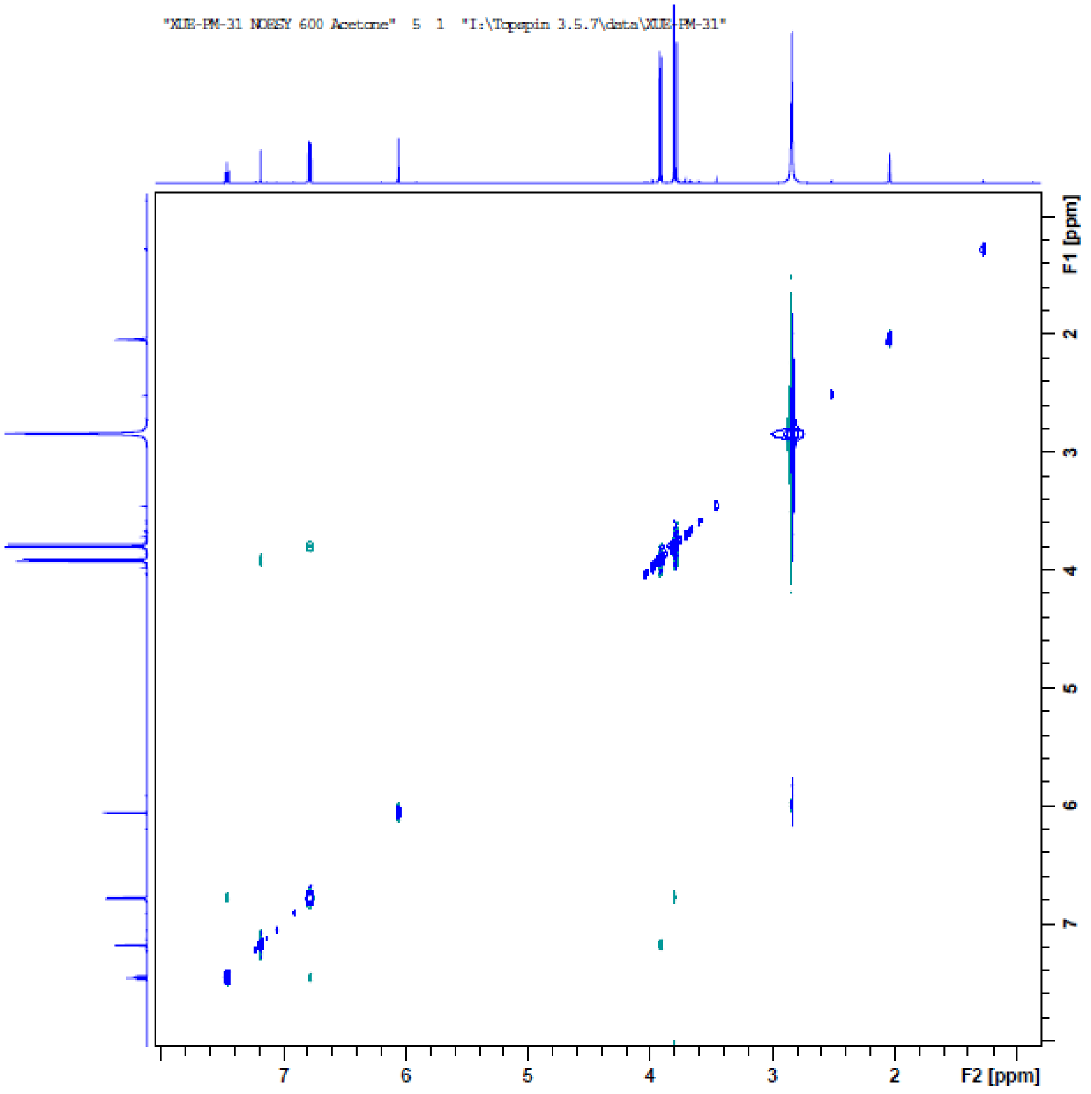

Figure S16: UV-Vis spectrum of compound 11

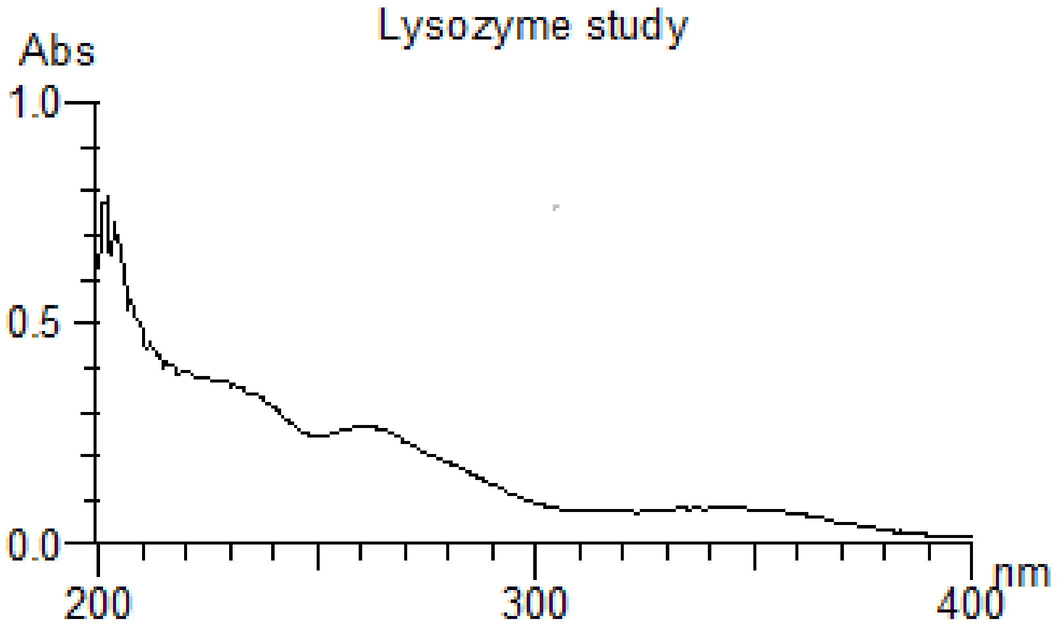

Figures S17: 1D and 2D NMR spectra of compound 1 ($CDCl_3$, 600 MHz)

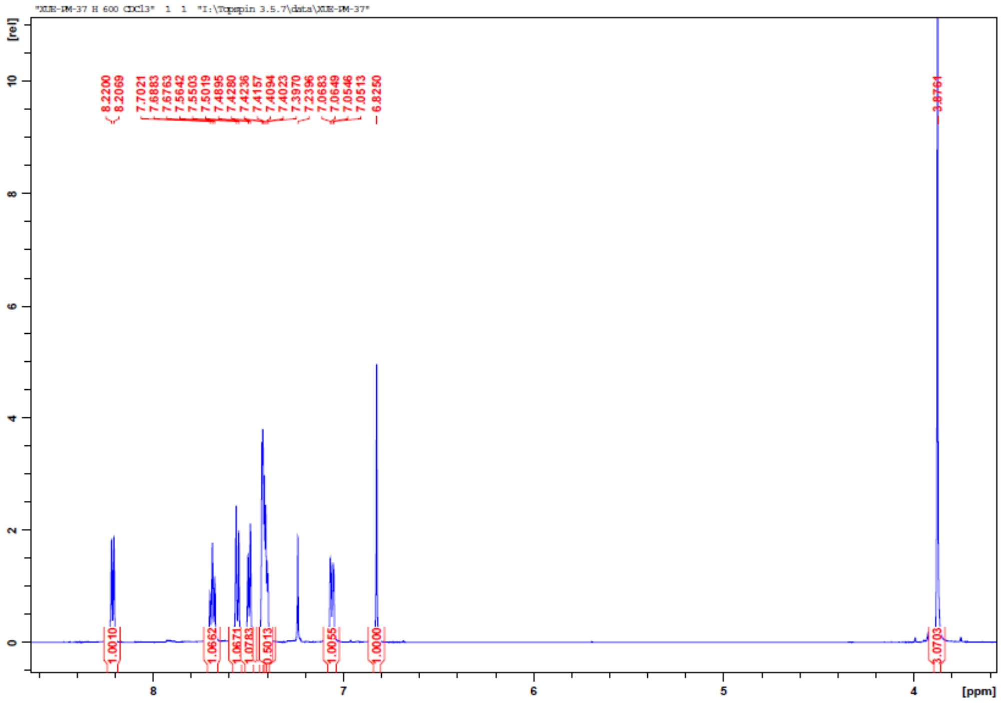

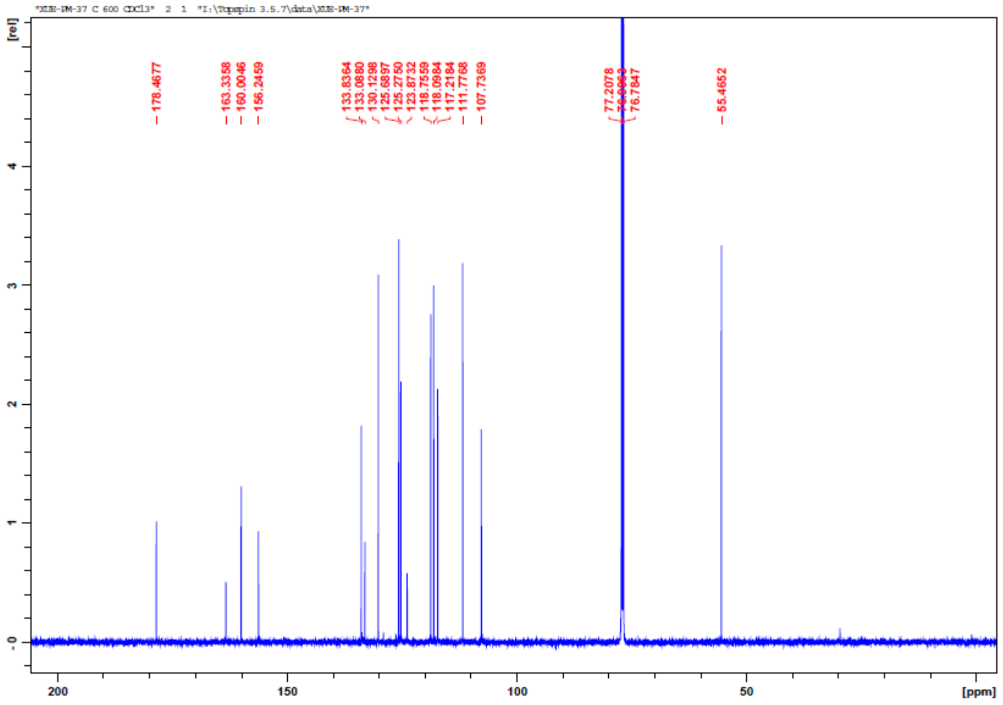

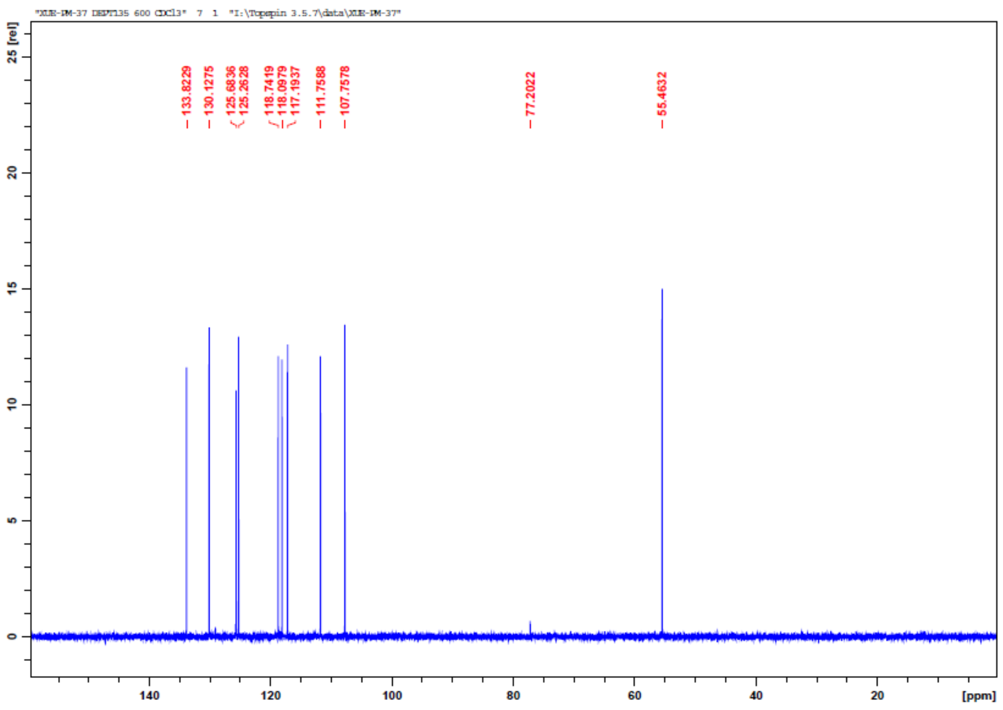
"XUE-PM-37 DEPT135 600 CDCl3" 7 1 "I:\Topspin 3.5.7\data\XUE-PM-37"
133.8229
130.1275
125.6836
125.2628
118.7419
118.0979
117.1937
111.7588
107.7578
77.2022
55.4632
[rel]
[ppm]

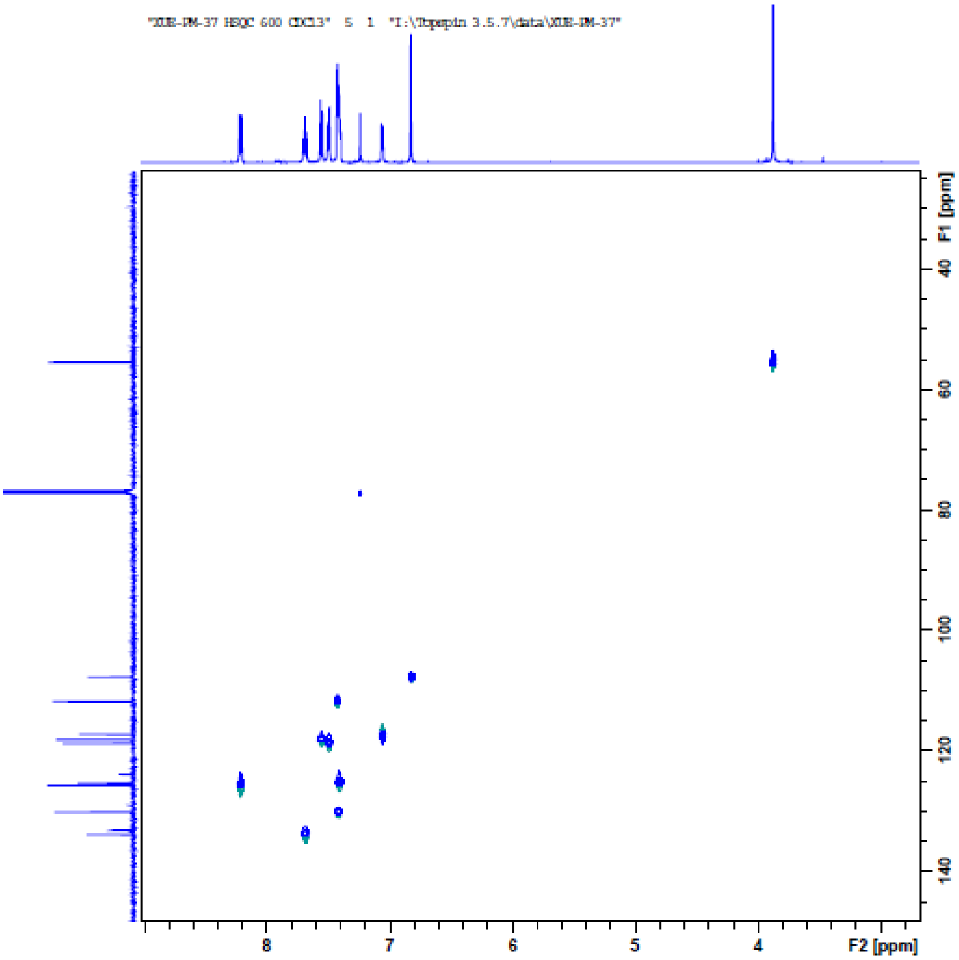
"XUE-PM-37 HSQC 600 CDCl3" 5 1 "I:\Topspin 3.5.7\data\XUE-PM-37"
F1 [ppm]
F2 [ppm]

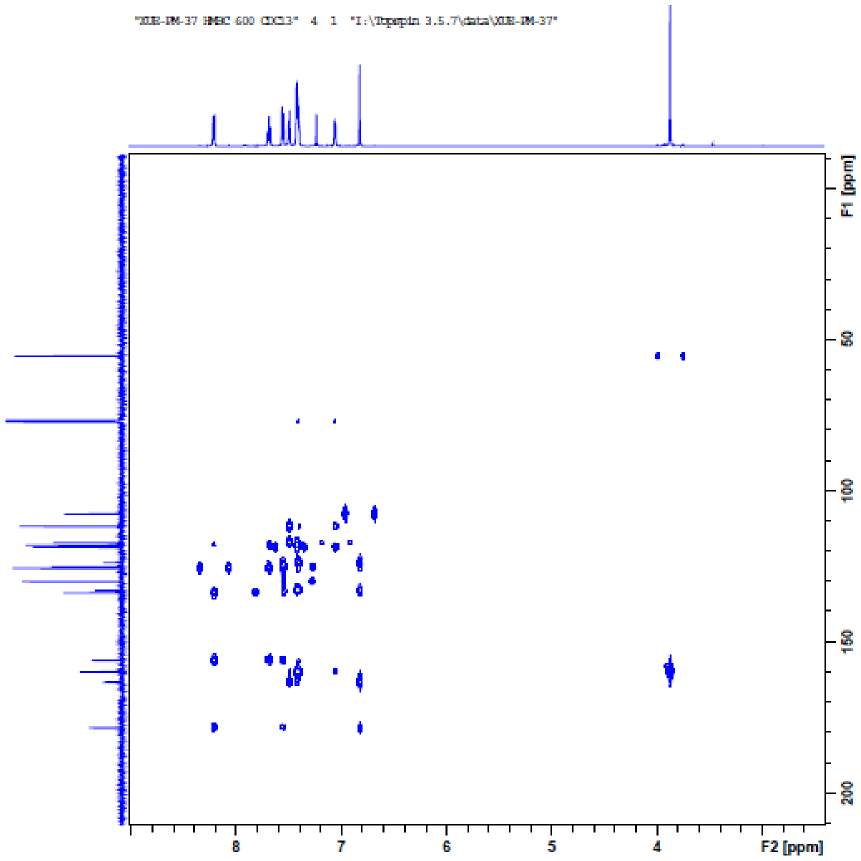
"XUB-PM-37 HMBC 600 CDCl3" 4 1 "I:\Topspin 3.5.7\data\XUB-PM-37"
F1 [ppm]
50
100
150
200
8
7
6
5
4
F2 [ppm]

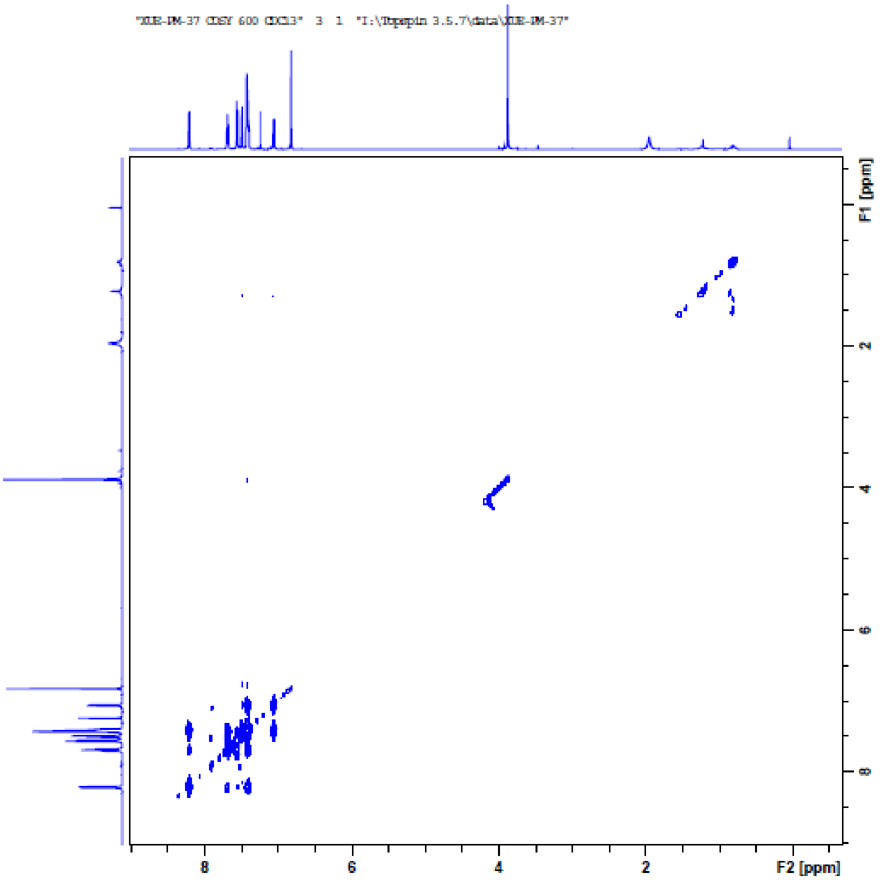
"XUB-PM-37 COSY 600 CDCl3" 3 1 "I:\Topspin 3.5.7\data\XUB-PM-37"
F1 [ppm]
2
4
6
8
8
6
4
2
F2 [ppm]

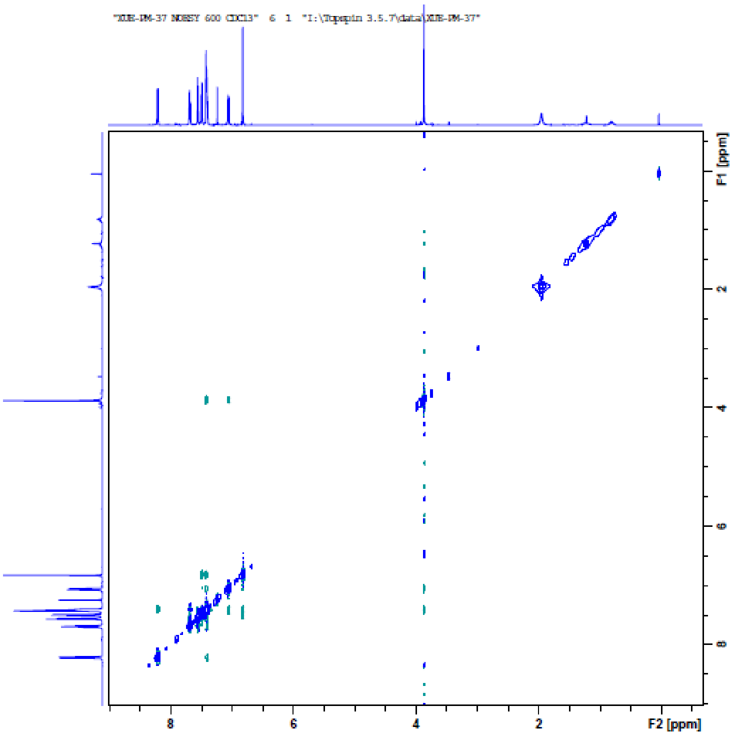

Figures S18: 1D and 2D NMR spectra of compound 2 ($CDCl_3$, 600MHz)

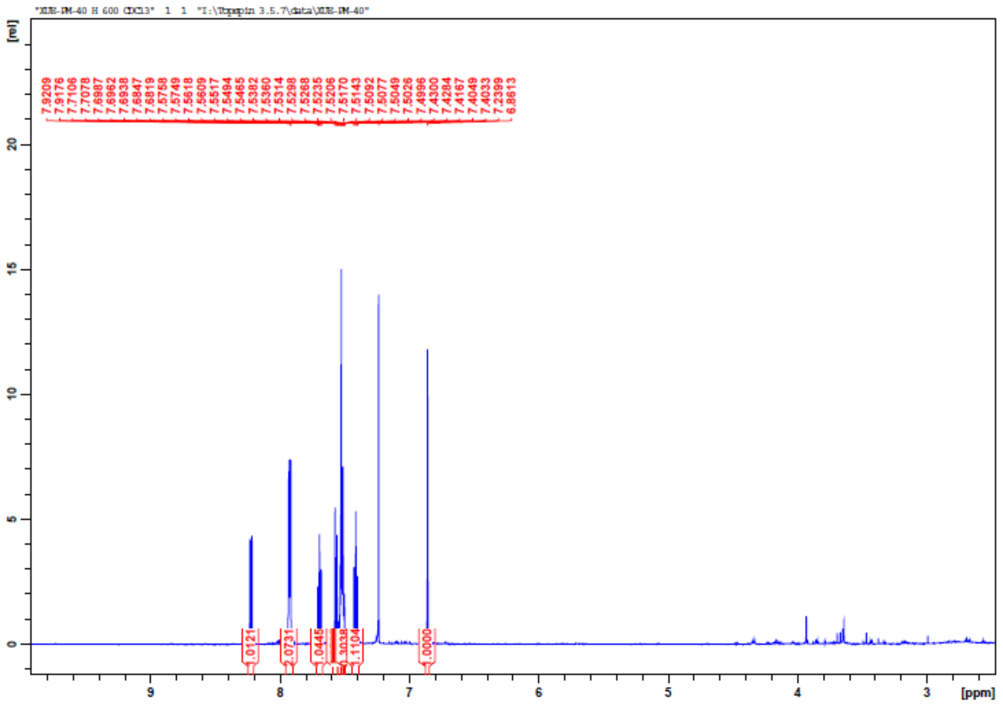

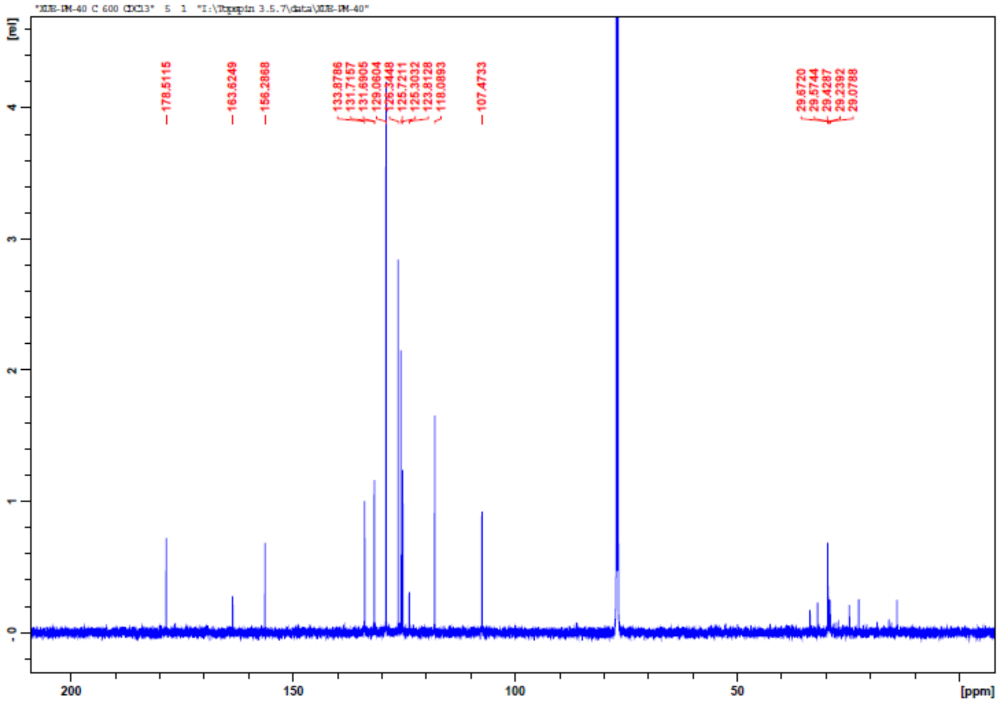

"XJB-PM-40 C 600 CDCl3" 5 1 "I:\Topspin 3.5.7\data\XJB-PM-40"
178.5115
163.6249
156.2868
133.8786
131.7157
131.6905
129.0604
126.3448
125.7211
125.3032
123.8128
118.0893
107.4733
29.6720
29.5744
29.4287
29.2392
29.0788
[rel]
[ppm]

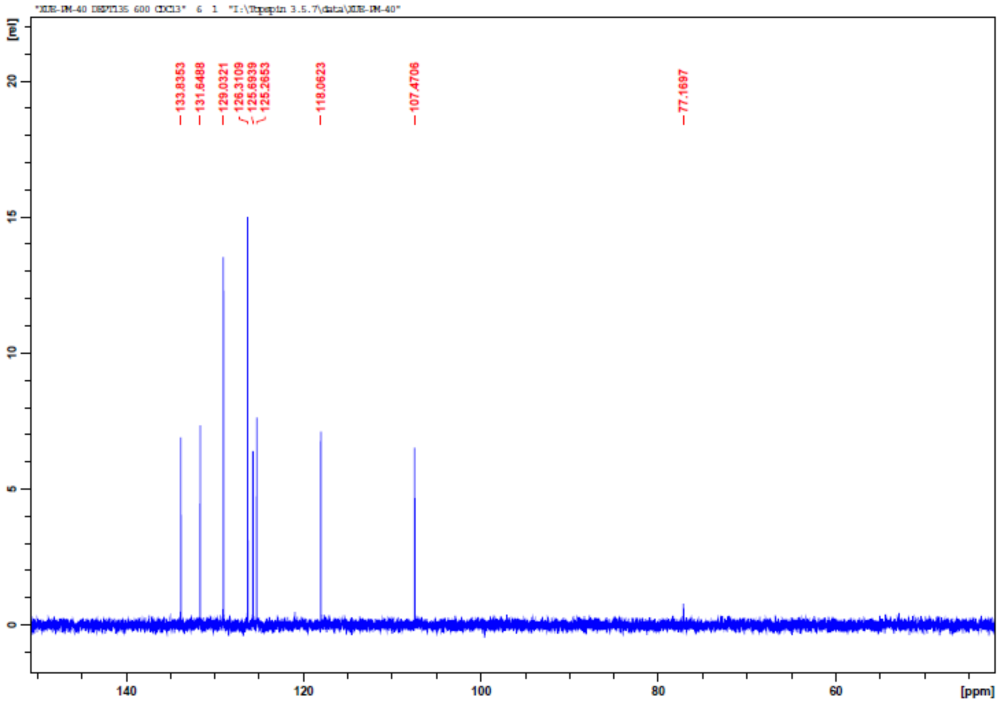

"XJB-PM-40 DEPT135 600 CDCl3" 6 1 "I:\Topspin 3.5.7\data\XJB-PM-40"
133.8353
131.6488
129.0321
126.3109
125.6939
125.2653
118.0623
107.4706
77.1697
[rel]
[ppm]

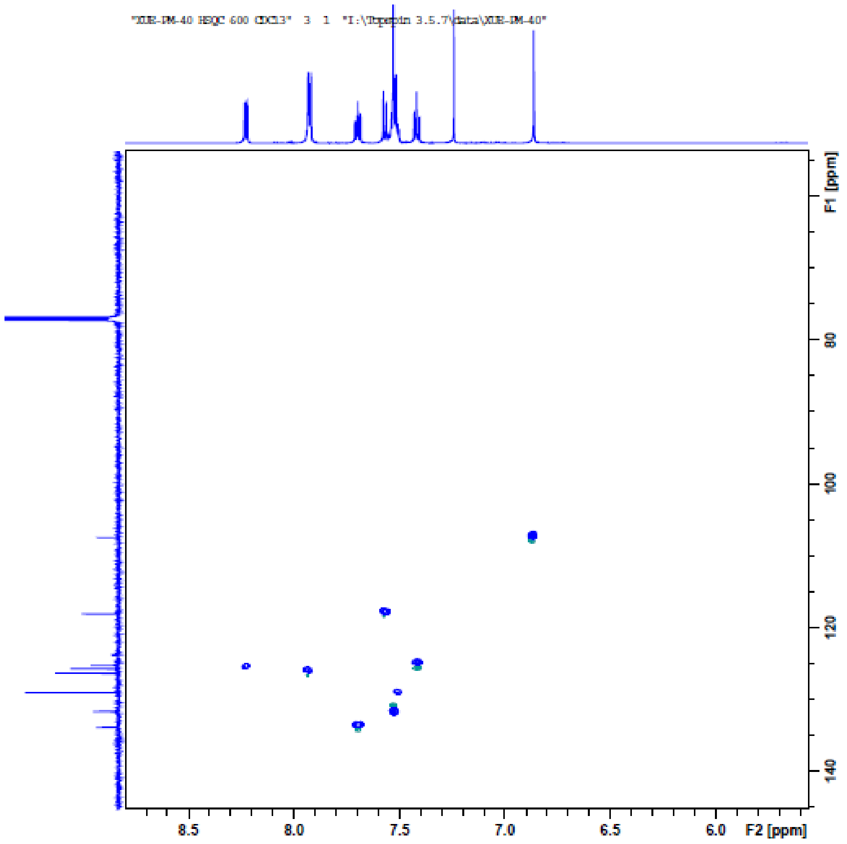
"XUE-PM-40 HSQC 600 CDCl3" 3 1 "I:\Topspin 3.5.7\data\XUE-PM-40"
F1 [ppm]
80
100
120
140
8.5
8.0
7.5
7.0
6.5
6.0
F2 [ppm]

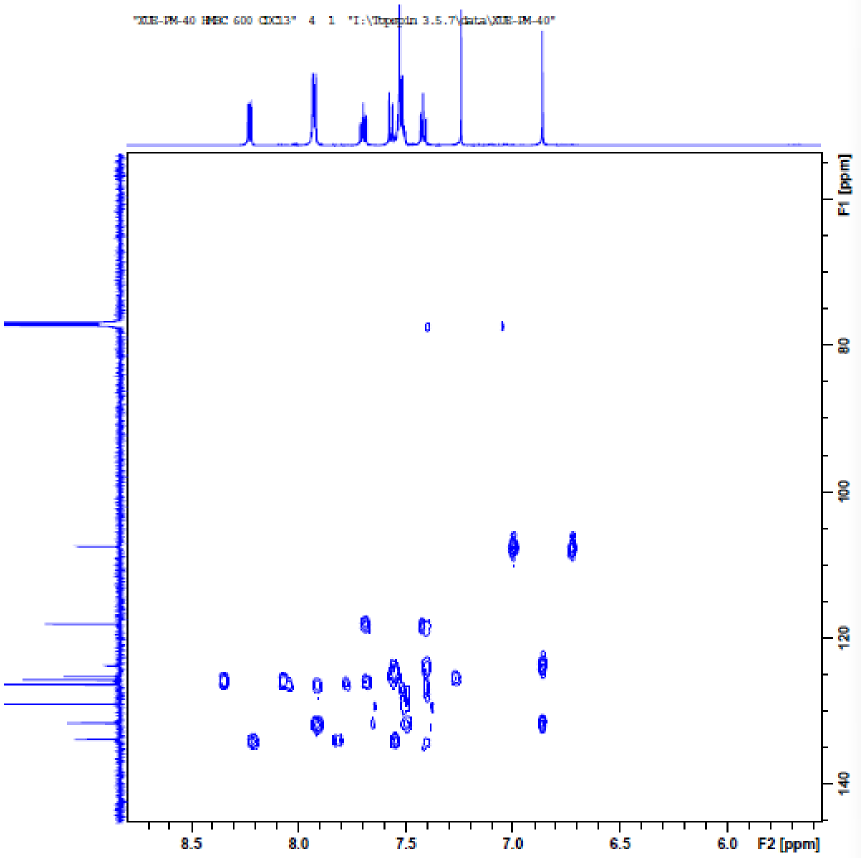
"XUE-PM-40 HMBC 600 CDCl3" 4 1 "I:\Topspin 3.5.7\data\XUE-PM-40"
F1 [ppm]
80
100
120
140
8.5
8.0
7.5
7.0
6.5
6.0
F2 [ppm]

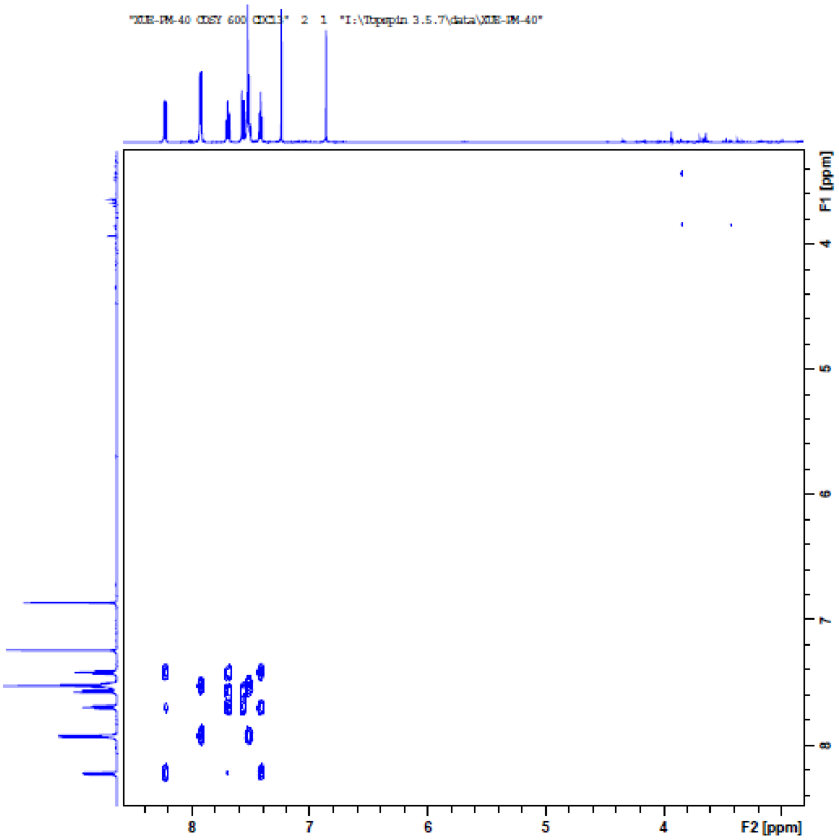
"XUE-PM-40 COSY 600 CDCl3" 2 1 "I:\Topspin 3.5.7\data\XUE-PM-40"
F1 [ppm]
F2 [ppm]

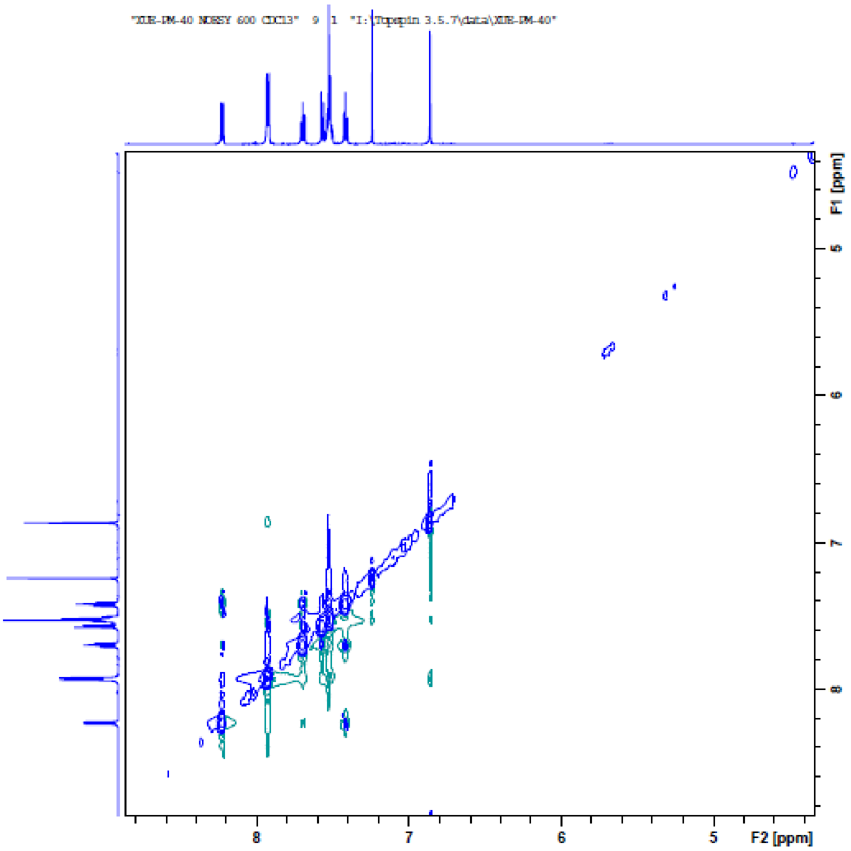
"XUE-PM-40 NOESY 600 CDCl3" 9 1 "I:\Topspin 3.5.7\data\XUE-PM-40"
F1 [ppm]
F2 [ppm]

Figures S19: 1D and 2D NMR spectra of compound 3 ($CDCl_3$, 600MHz)

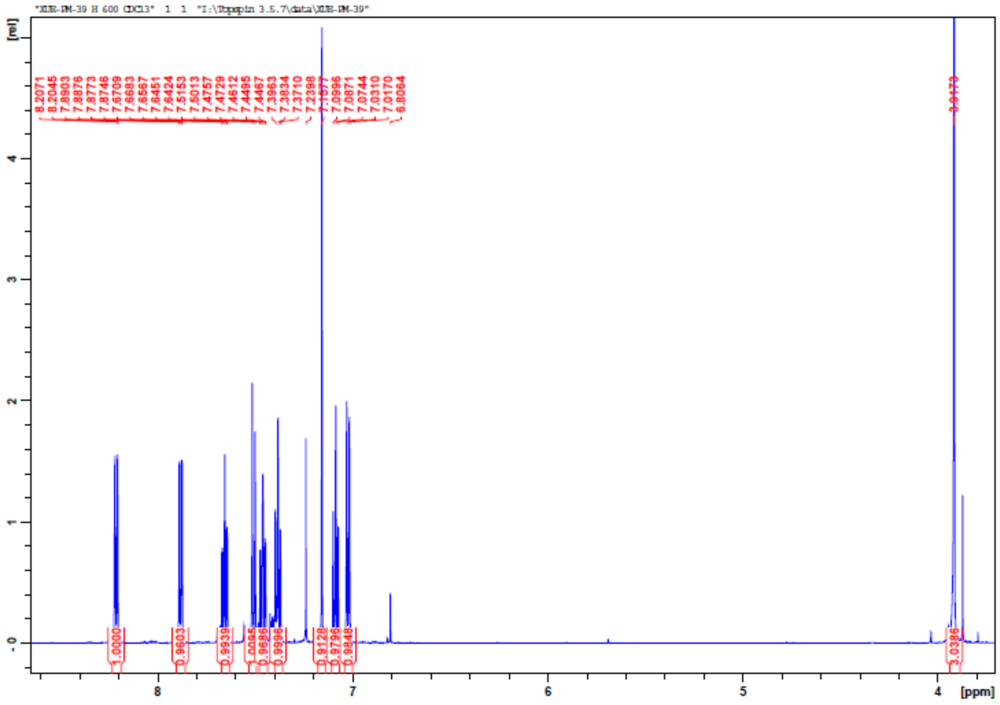

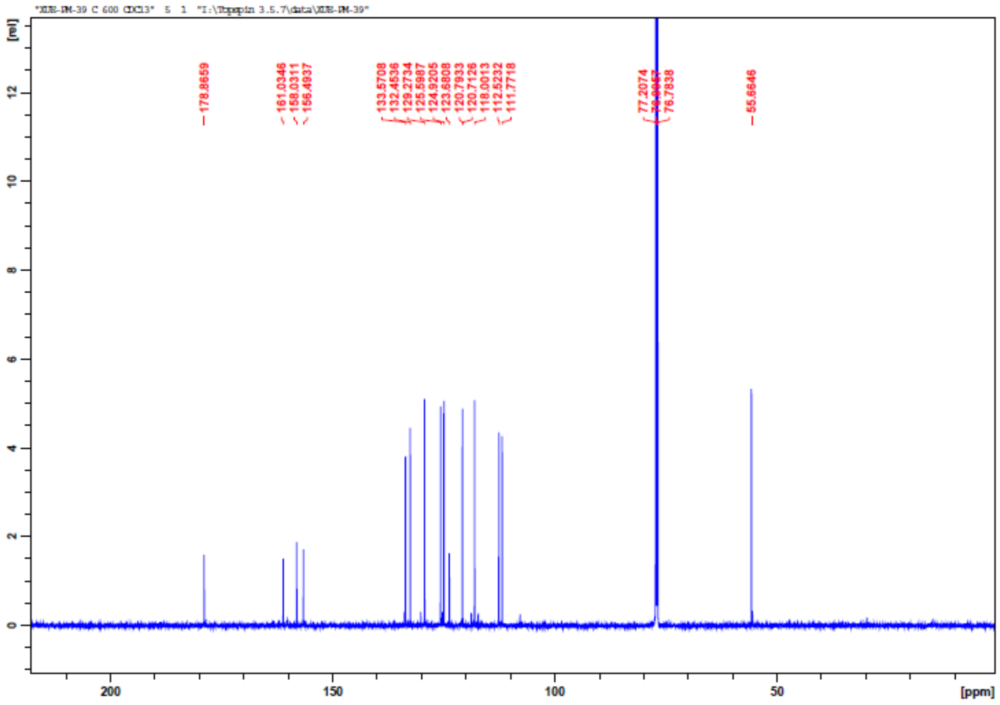

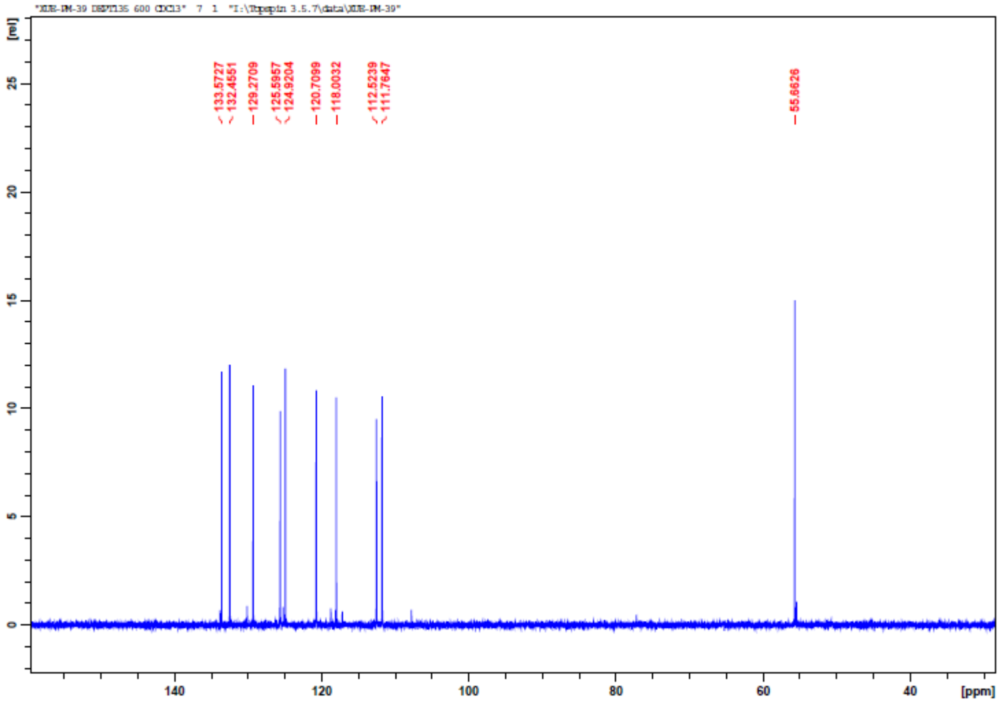
"XUE-PM-39 DEPT135 600 CDCl3" 7 1 "I:\Topspin 3.5.7\data\XUE-PM-39"
133.5727
132.4551
129.2709
125.5957
124.9204
120.7099
118.0032
112.5239
111.7647
55.6626
[rel]
[ppm]

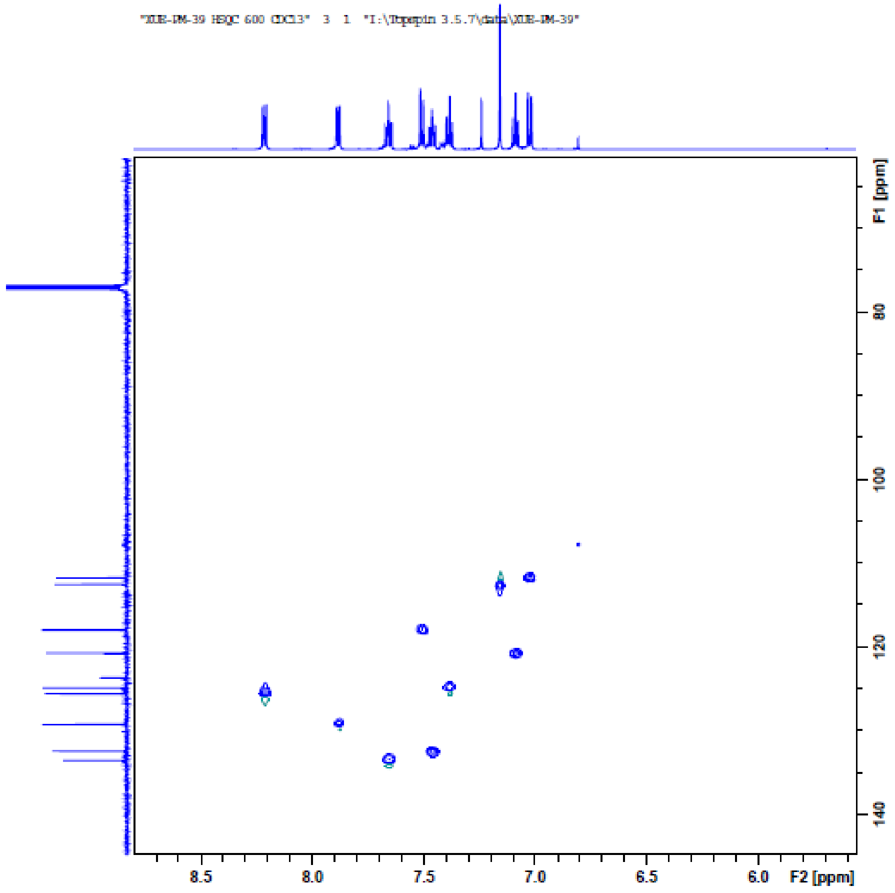
"XUE-PM-39 HSQC 600 CDCl3" 3 1 "I:\Topspin 3.5.7\data\XUE-PM-39"
F1 [ppm]
F2 [ppm]

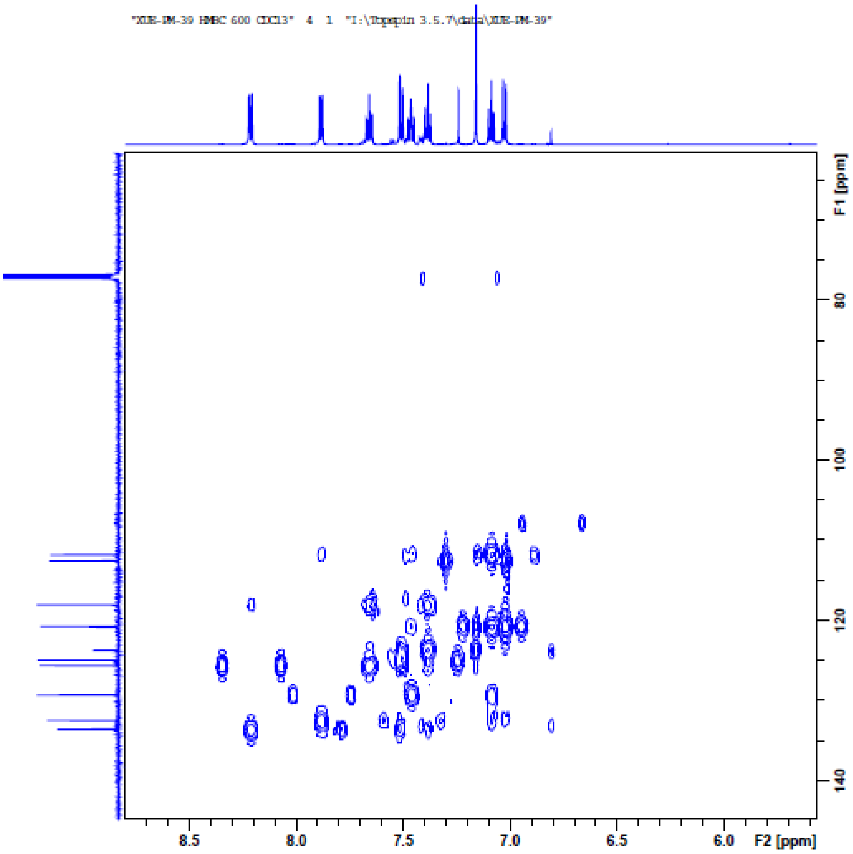
"XUB-PM-39 HMBC 600 CDCl3" 4 1 "I:\Topspin 3.5.7\data\XUB-PM-39"
F1 [ppm]
80
100
120
140
8.5
8.0
7.5
7.0
6.5
6.0
F2 [ppm]

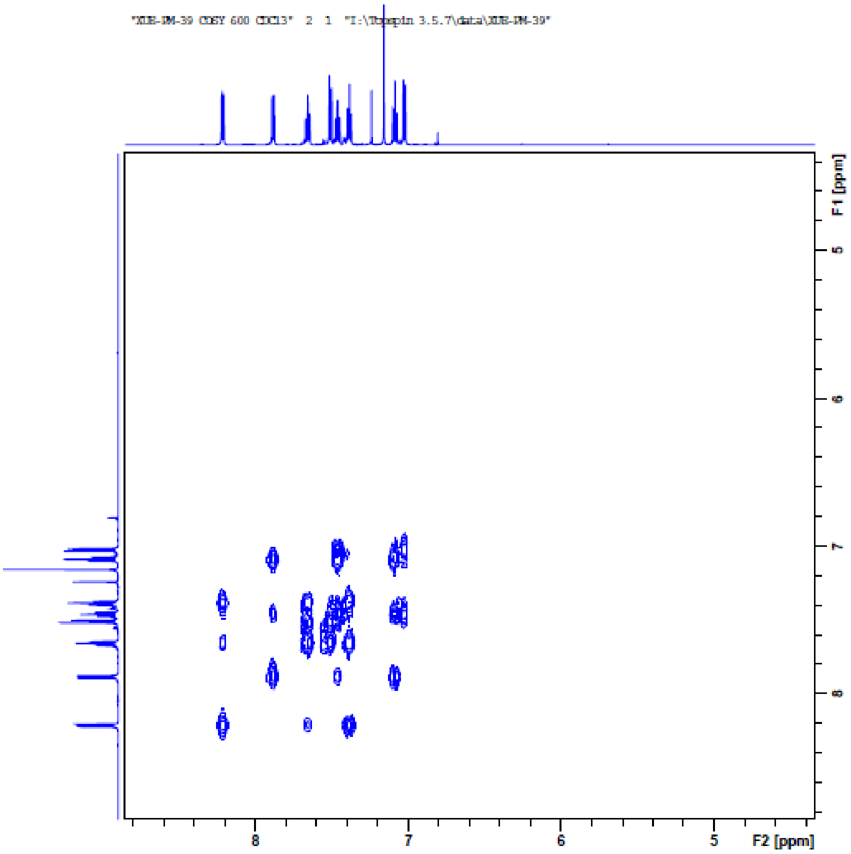
"XUB-PM-39 COSY 600 CDCl3" 2 1 "I:\Topspin 3.5.7\data\XUB-PM-39"
F1 [ppm]
5
6
7
8
8
7
6
5
F2 [ppm]

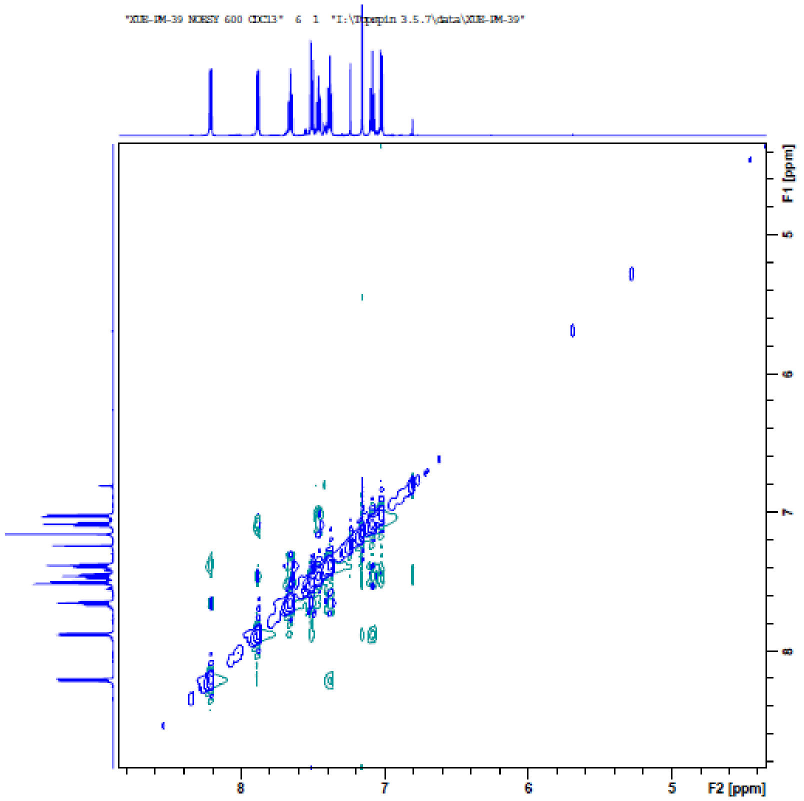

Figures S20: 1D and 2D NMR spectra of compound 4 ($CDCl_3$, 600MHz)

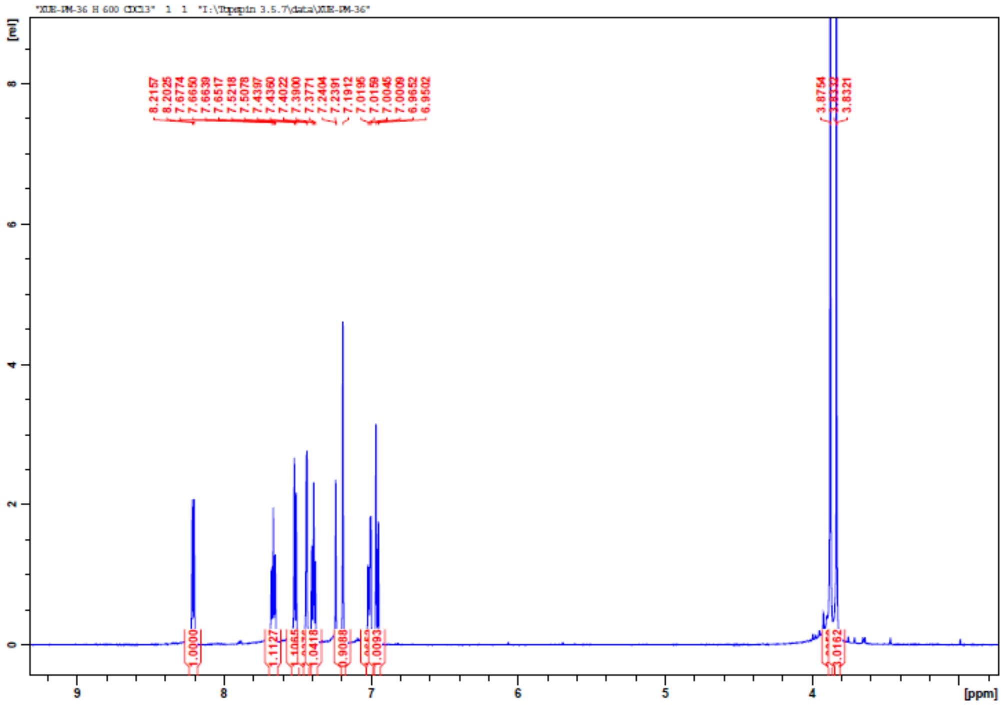

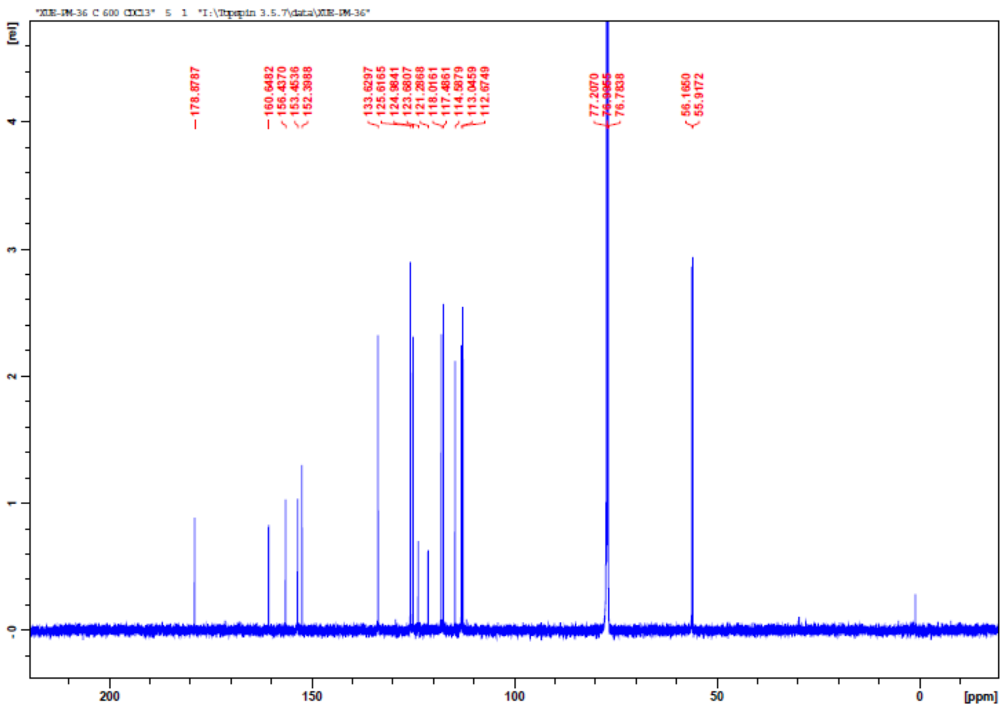
"XUB-PM-36 C 600 CDCl3" 5 1 "I:\Topspin 3.5.7\data\XUB-PM-36"
178.8787
160.6482
156.4370
153.4536
152.3988
133.6297
125.6165
124.9841
123.6807
121.2868
118.0161
117.4861
114.5879
113.0459
112.6749
77.2070
76.7838
56.1650
55.9172
[rel]
[ppm]

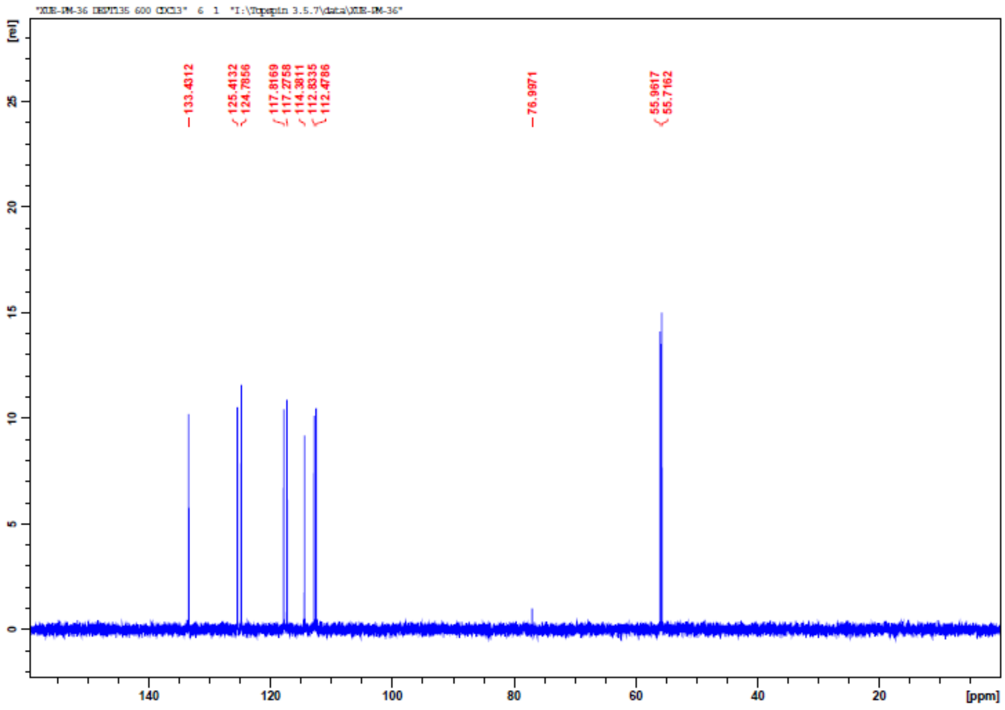
"XUB-PM-36 DEPT135 600 CDCl3" 6 1 "I:\Topspin 3.5.7\data\XUB-PM-36"
133.4312
125.4132
124.7856
117.8169
117.2758
114.3811
112.8335
112.4786
76.9971
55.9617
55.7162
[rel]
[ppm]

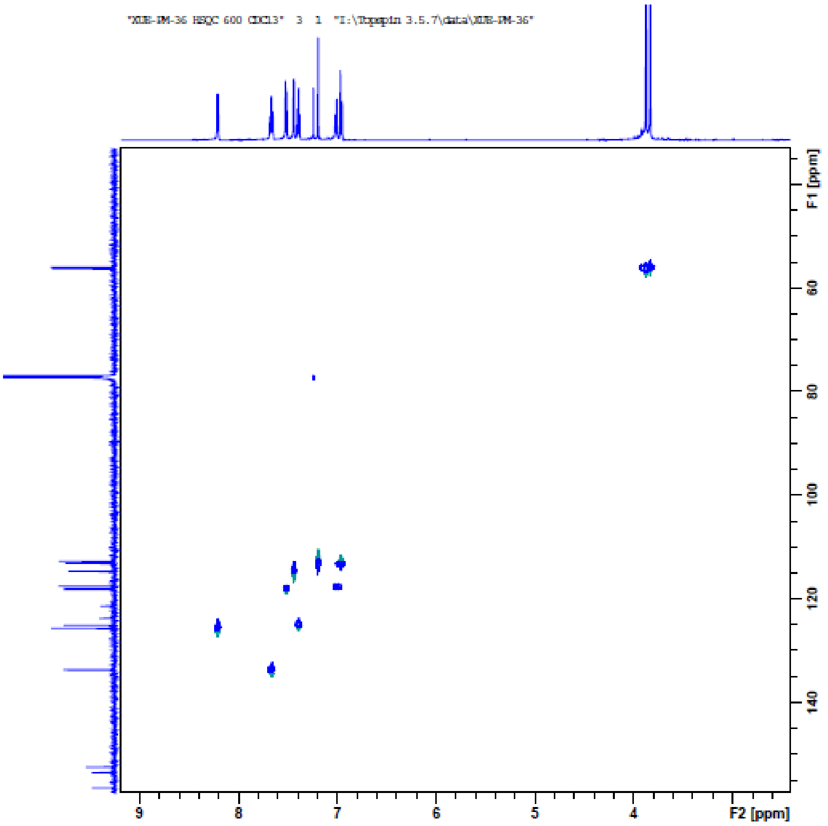
"XUB-PM-36 HSQC 600 CDCl3" 3 1 "I:\Topspin 3.5.7\data\XUB-PM-36"
F1 [ppm]
F2 [ppm]

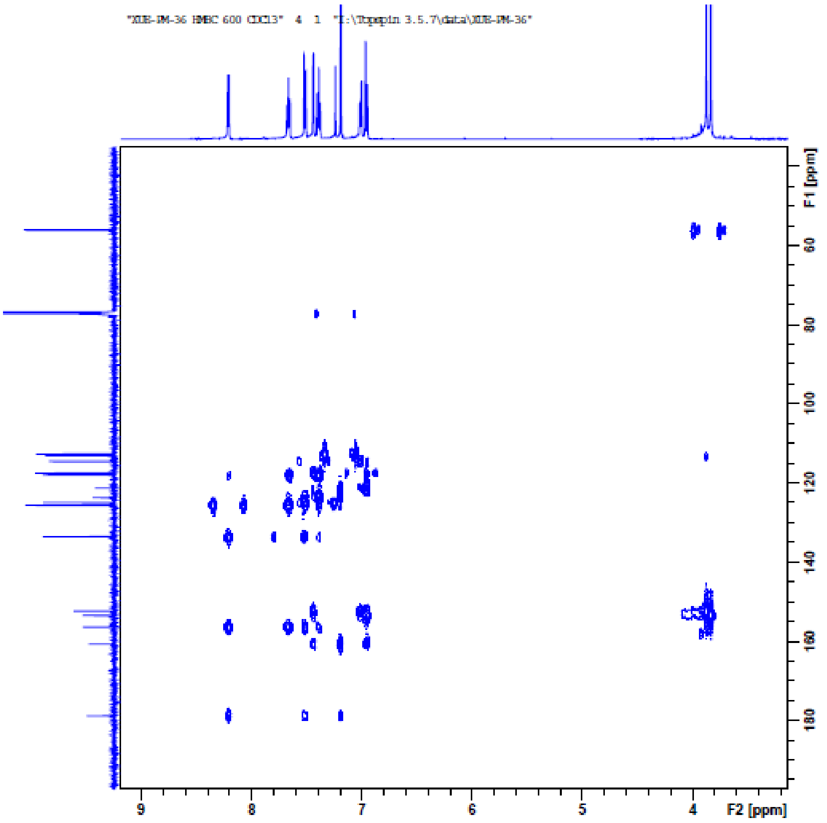
"XUB-PM-36 HMBC 600 CDCl3" 4 1 "I:\Topspin 3.5.7\data\XUB-PM-36"
F1 [ppm]
F2 [ppm]

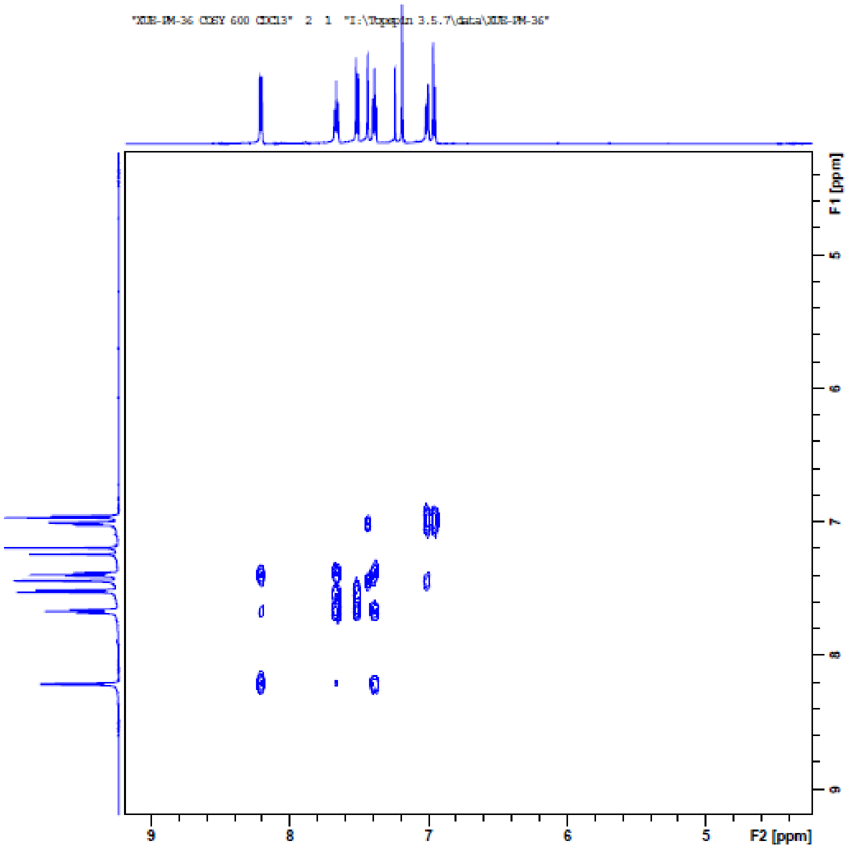
F1 [ppm]
F2 [ppm]
9 8 7 6 5

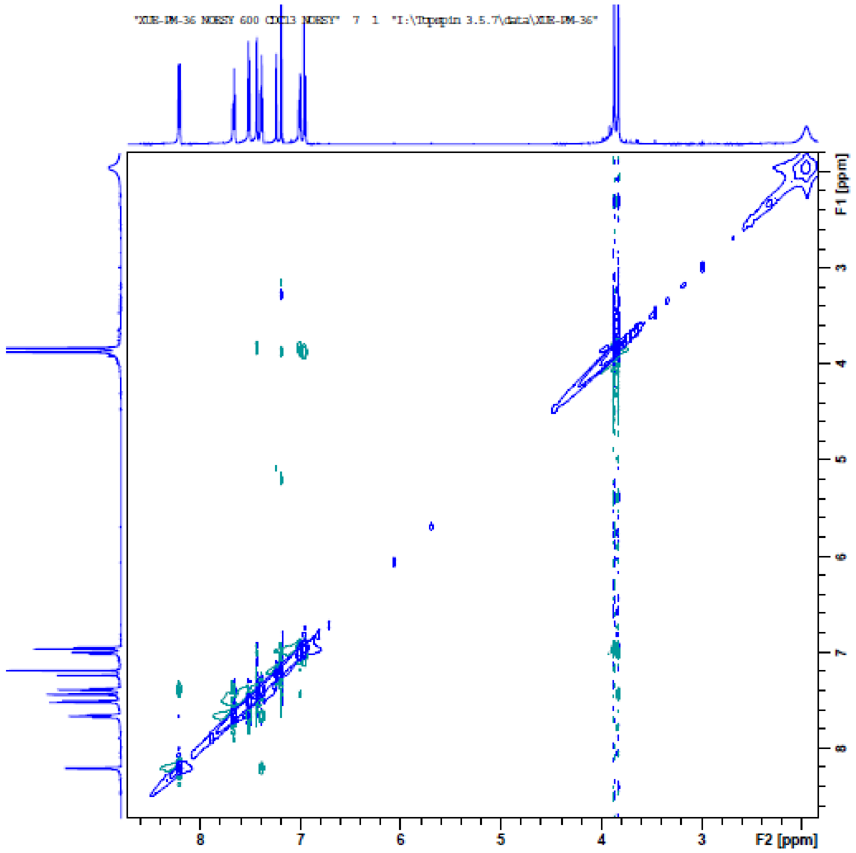
F1 [ppm]
F2 [ppm]
8 7 6 5 4 3

Figures S21: 1D and 2D NMR spectra of compound 6 (methanol-d_4, 600MHz)

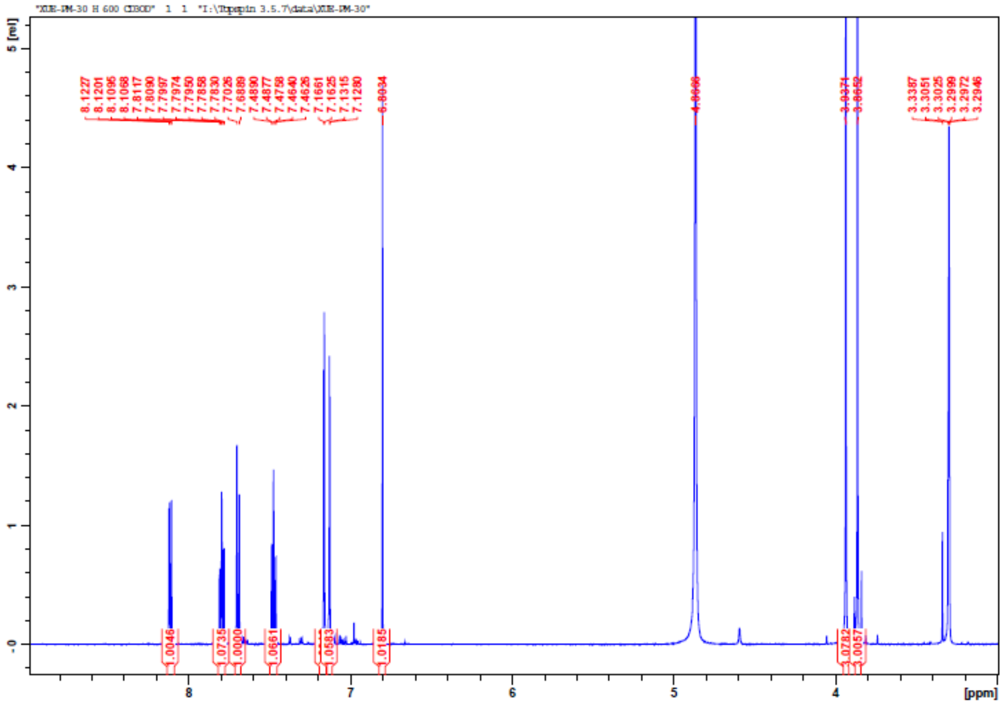

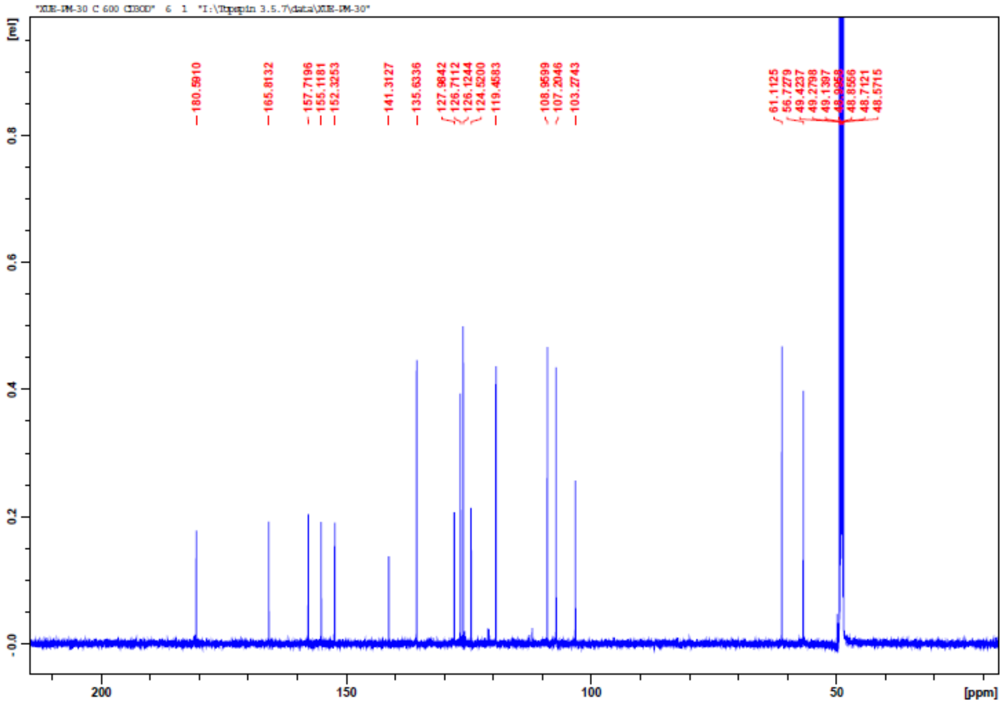

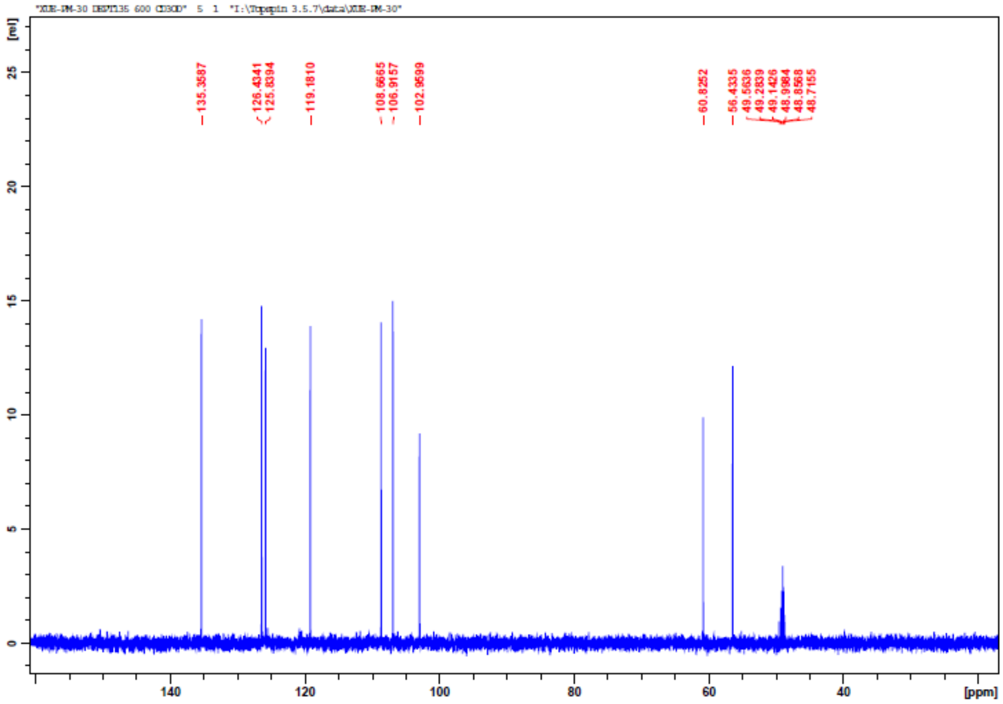
135.3587
126.4341
125.8394
119.1810
108.6665
106.9157
102.9599
60.8252
56.4335
49.5636
49.2839
49.1426
48.9984
48.8568
48.7155
[rel]
[ppm]

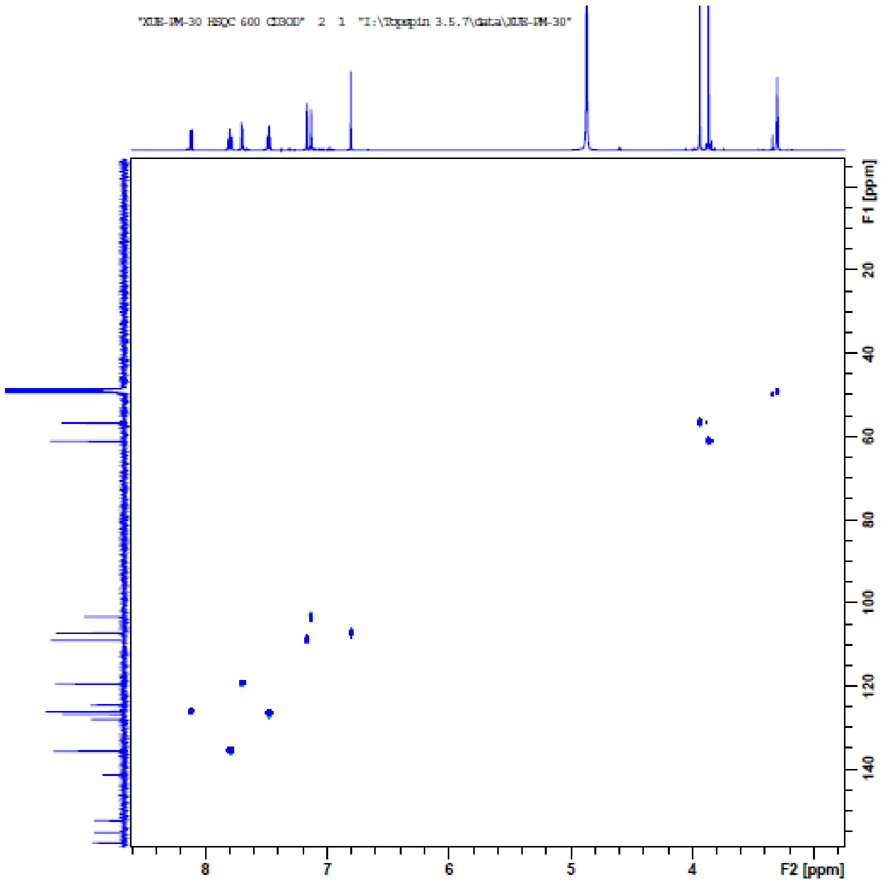
F1 [ppm]
F2 [ppm]

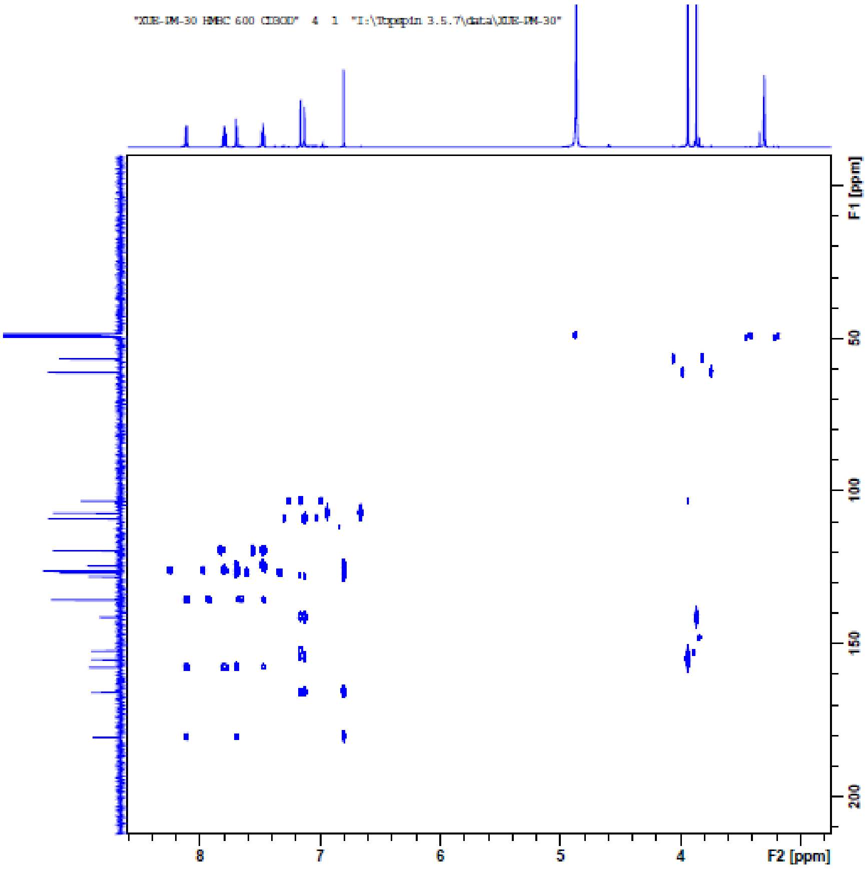
"XUE-PM-30 HMBC 600 CD3OD" 4 1 "I:\Topspin 3.5.7\data\XUE-PM-30"
F1 [ppm]
50
100
150
200
8
7
6
5
4
F2 [ppm]

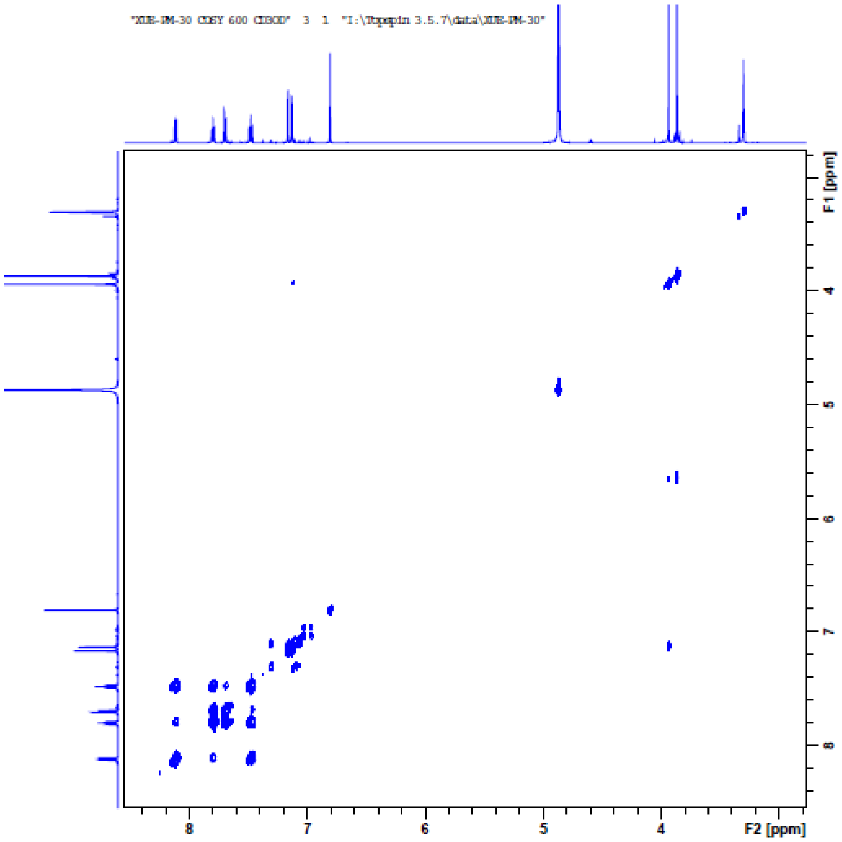
"XUE-PM-30 COSY 600 CD3OD" 3 1 "I:\Topspin 3.5.7\data\XUE-PM-30"
F1 [ppm]
4
5
6
7
8
8
7
6
5
4
F2 [ppm]

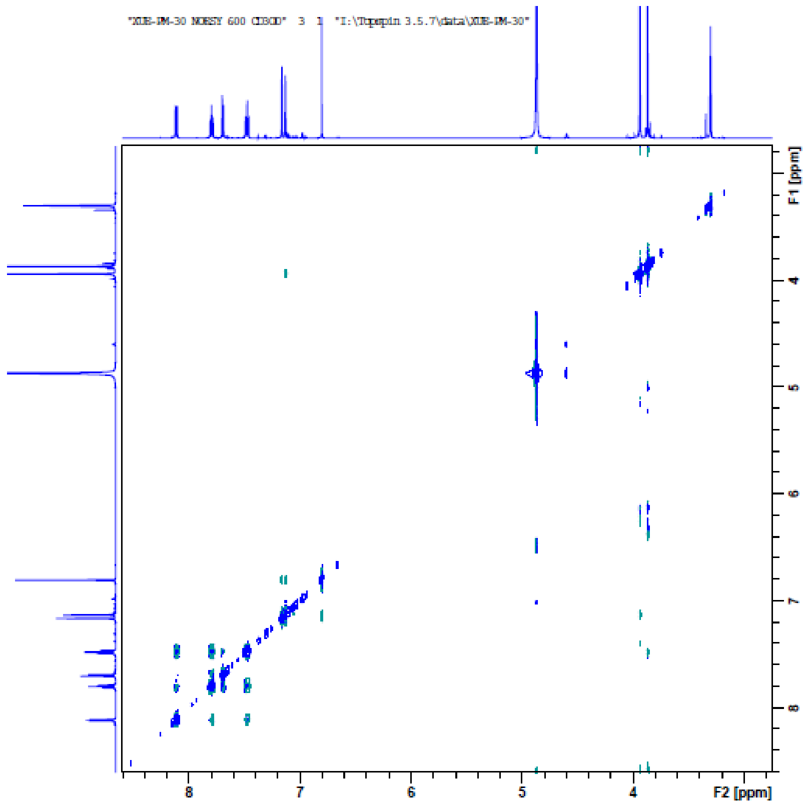

Figures S22: 1D and 2D NMR spectra of compound 7 (methanol-d_4, 600MHz)

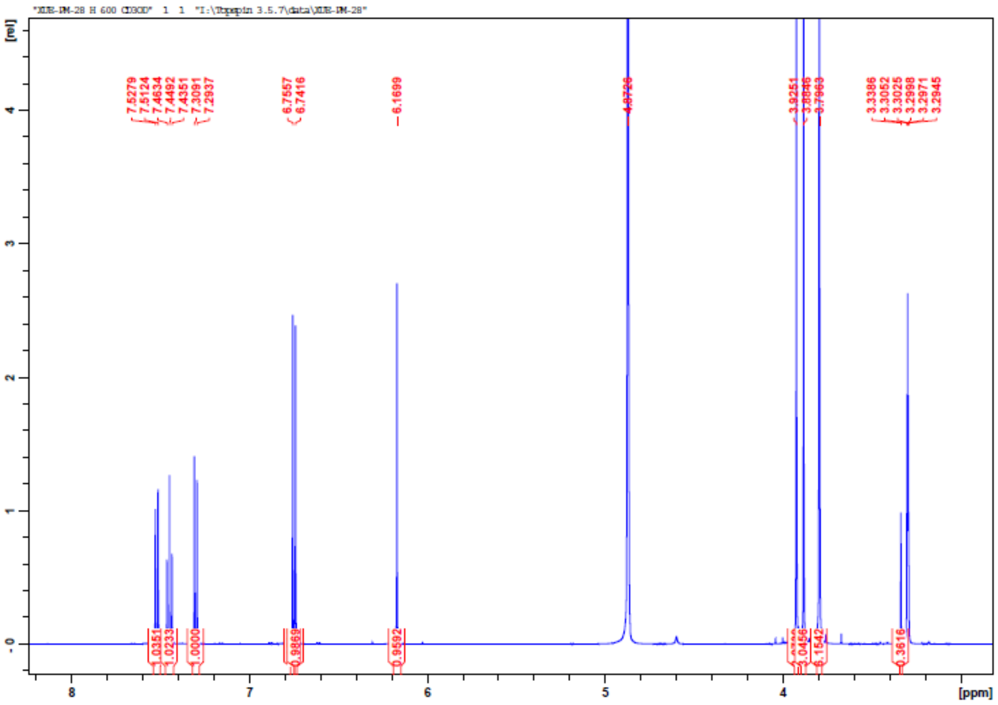

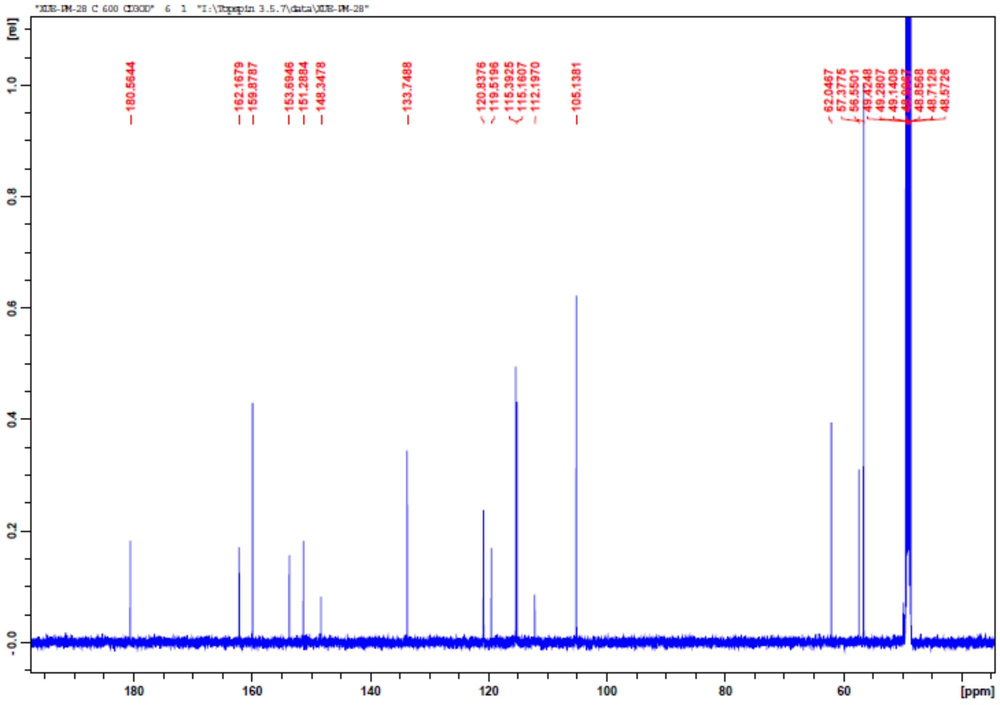

[rel]
180.5644
162.1679
159.8787
153.6946
151.2884
148.3478
133.7488
120.8376
119.5196
115.3925
115.1607
112.1970
105.1381
62.0467
57.3775
56.5501
49.4248
49.2807
49.1408
48.8568
48.7128
48.5726
[ppm]

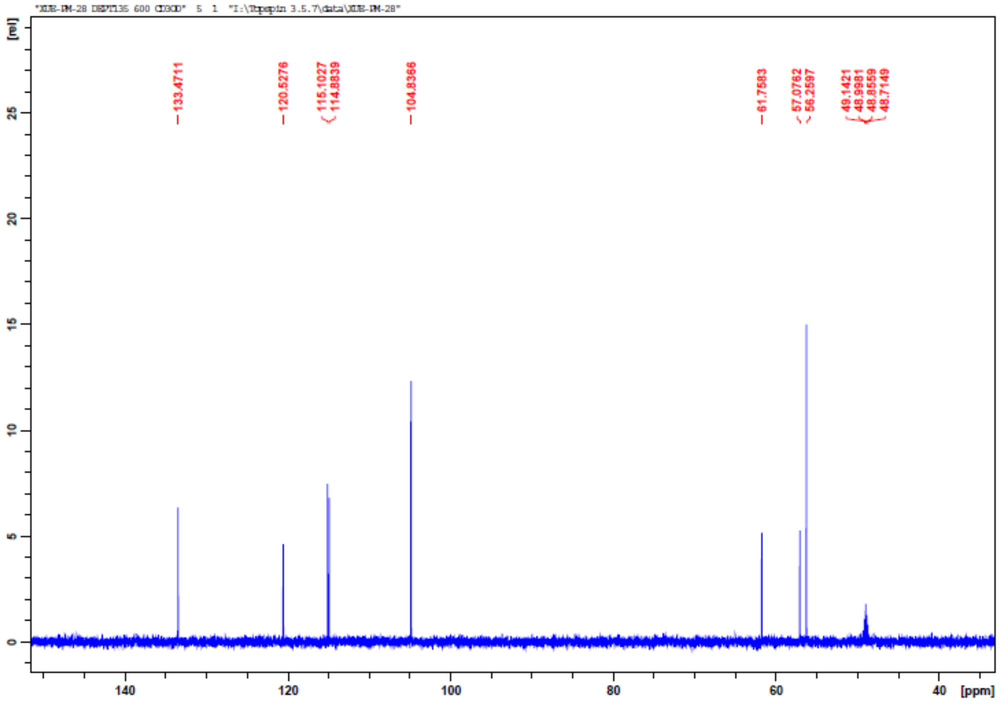

[rel]
133.4711
120.5276
115.1027
114.8839
104.8366
61.7583
57.0762
56.2597
49.1421
48.9981
48.8559
48.7149
[ppm]

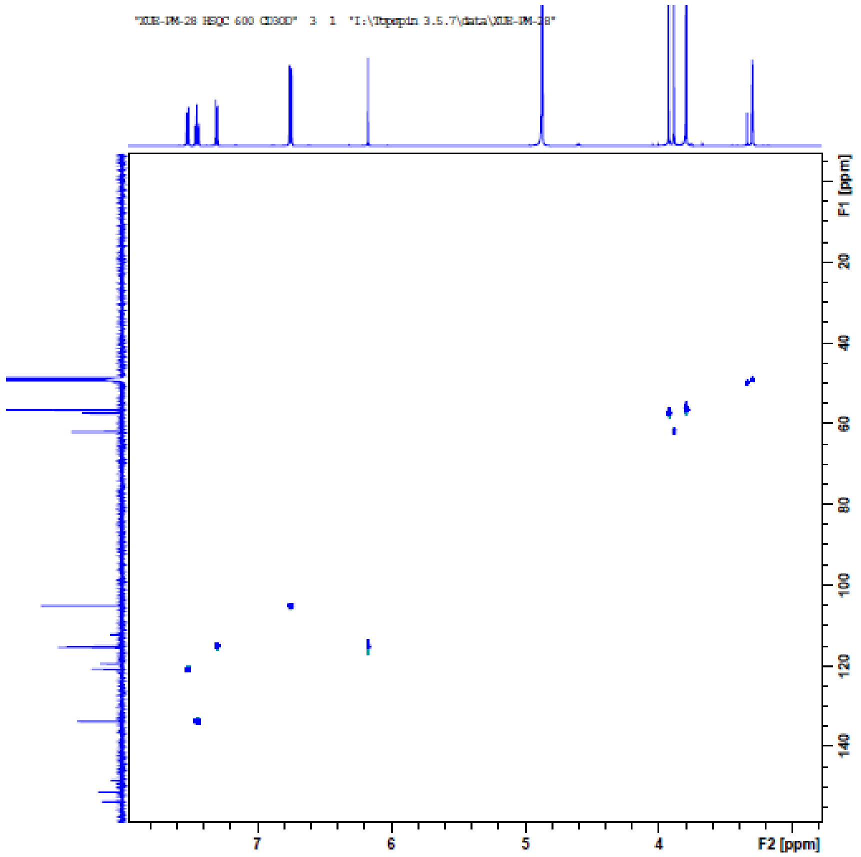
"XUE-PM-28 HSQC 600 CD3OD" 3 1 "I:\Topspin 3.5.7\data\XUE-PM-28"
F1 [ppm]
F2 [ppm]

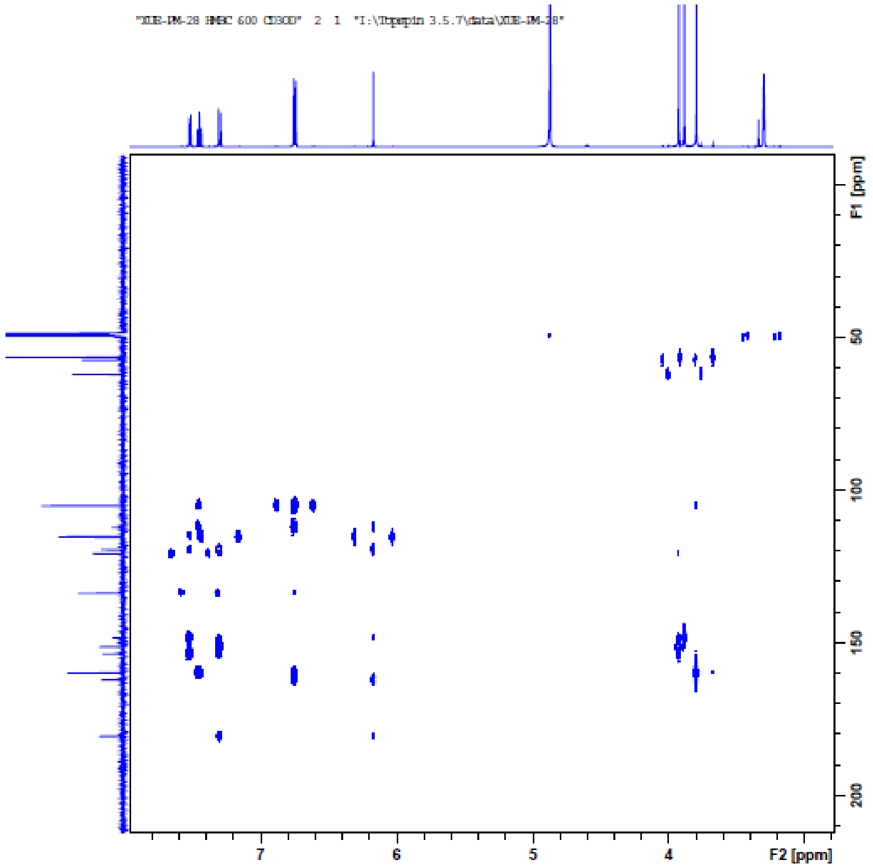
"XUE-PM-28 HMBC 600 CD3OD" 2 1 "I:\Topspin 3.5.7\data\XUE-PM-28"
F1 [ppm]
F2 [ppm]

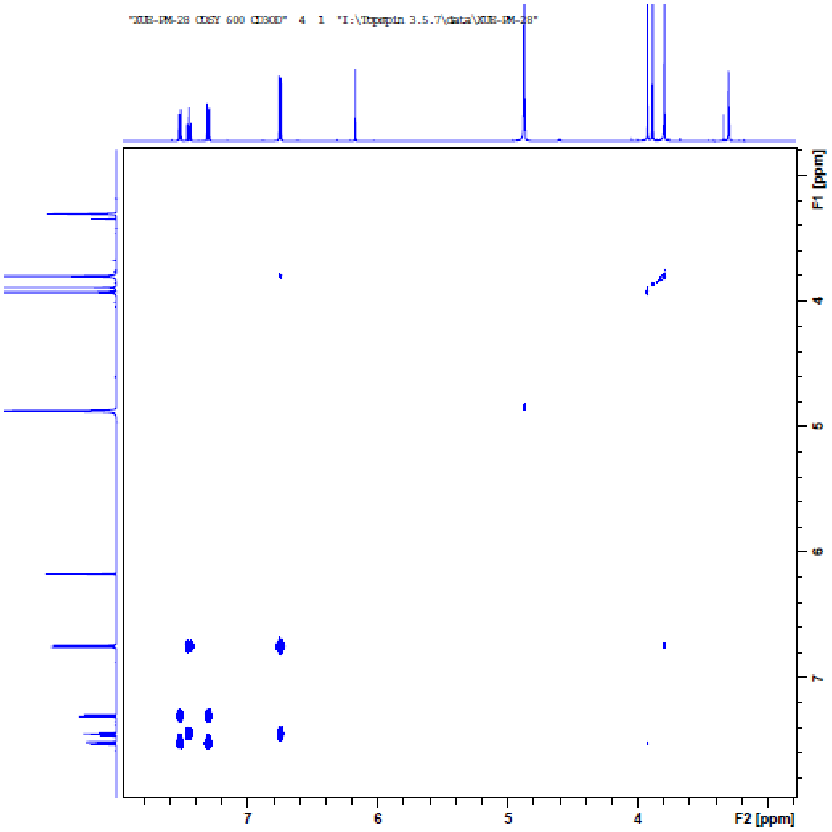

"XUE-PM-28 COSY 600 CD3OD" 4 1 "I:\Topspin 3.5.7\data\XUE-PM-28"
F1 [ppm]
4
5
6
7
7
6
5
4
F2 [ppm]

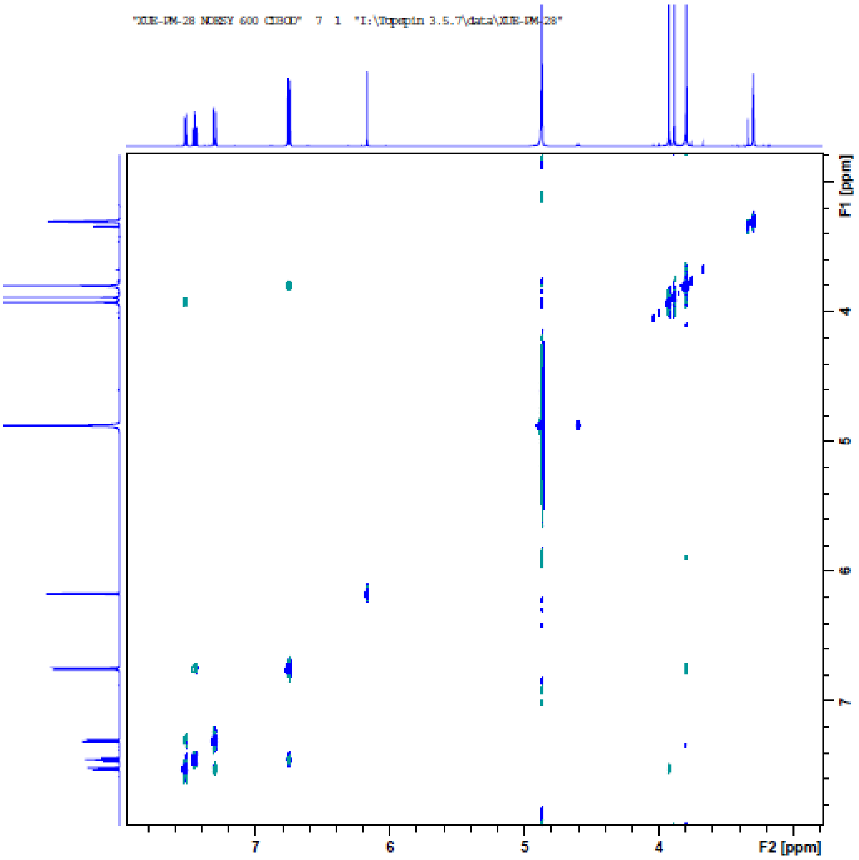

"XUE-PM-28 NOESY 600 CD3OD" 7 1 "I:\Topspin 3.5.7\data\XUE-PM-28"
F1 [ppm]
4
5
6
7
7
6
5
4
F2 [ppm]

Figures S23: 1D and 2D NMR spectra of compound 8 (methanol-d_4, 600MHz)

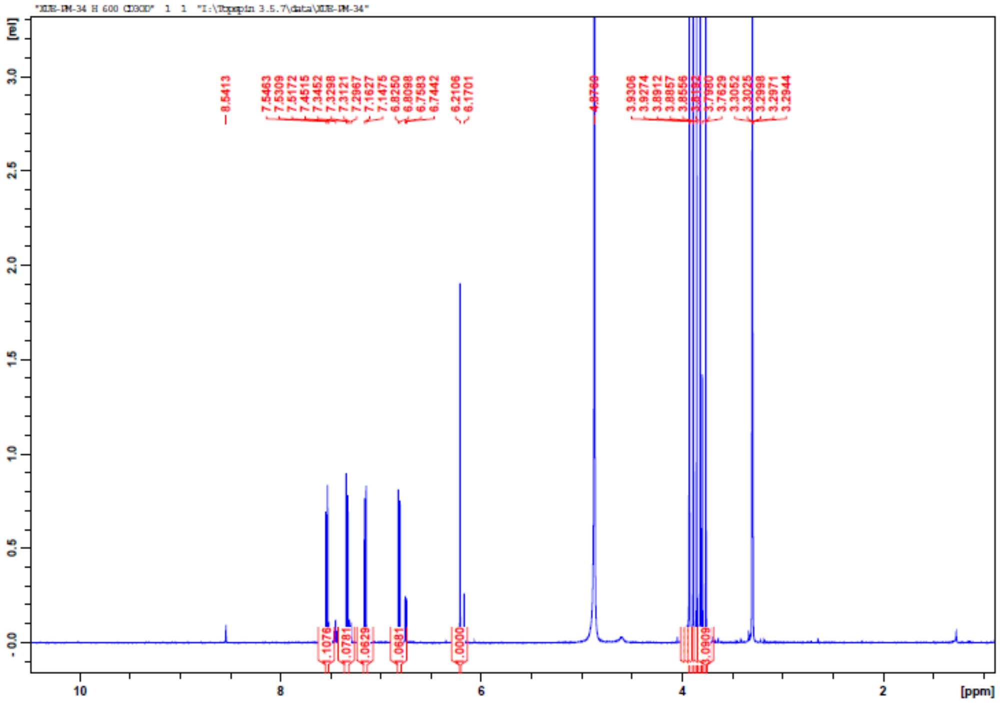

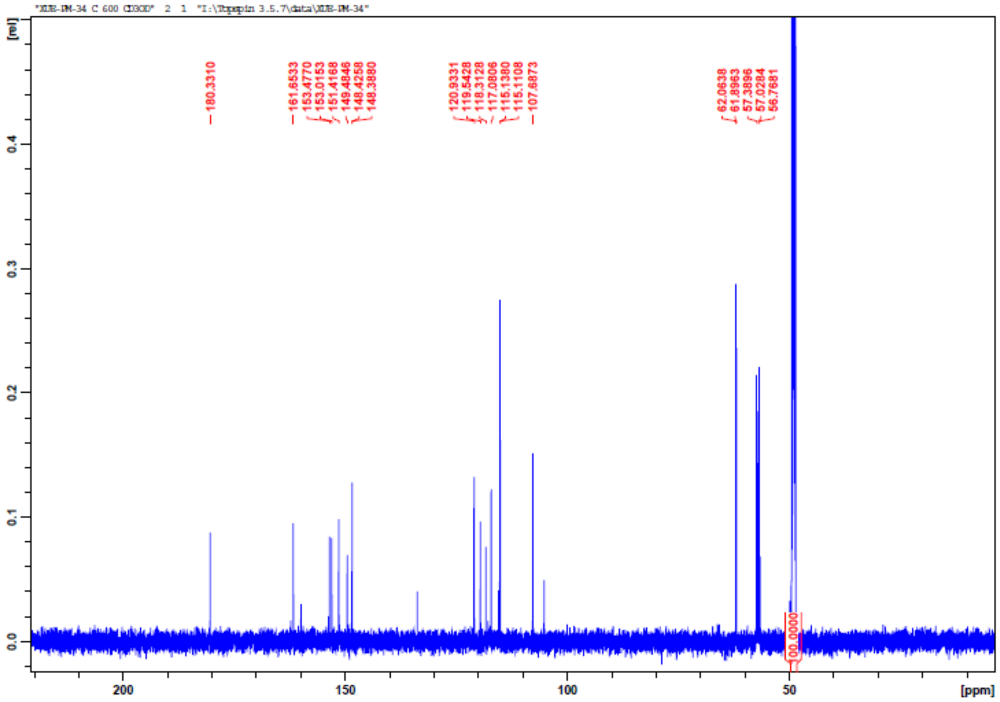

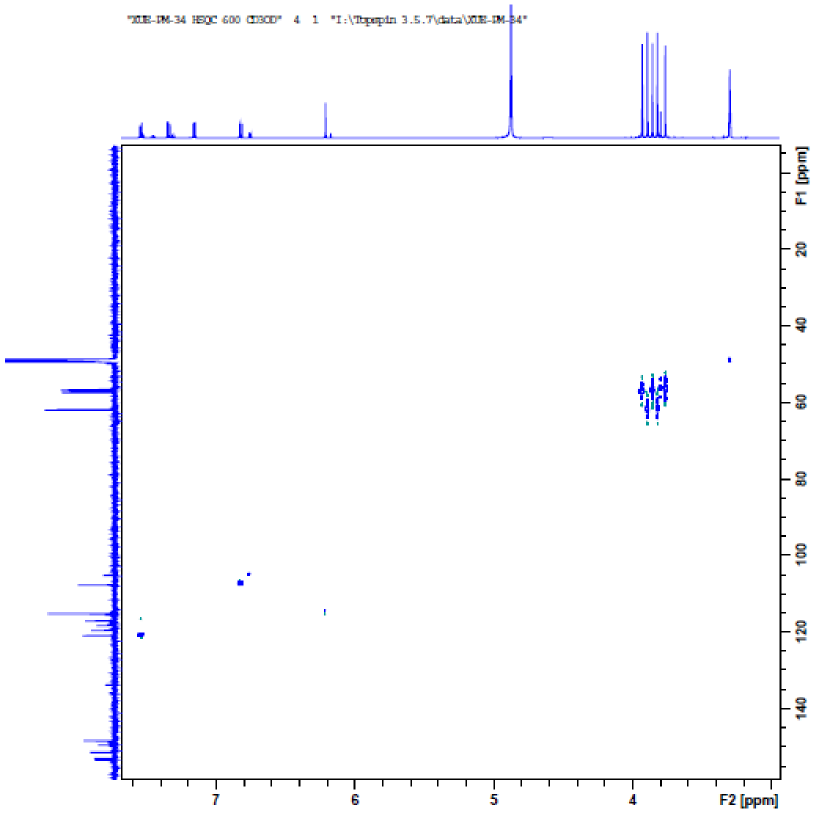
"XUB-PM-34 HSQC 600 CD3OD" 4 1 "I:\Topspin 3.5.7\data\XUB-PM-34"
F1 [ppm]
F2 [ppm]

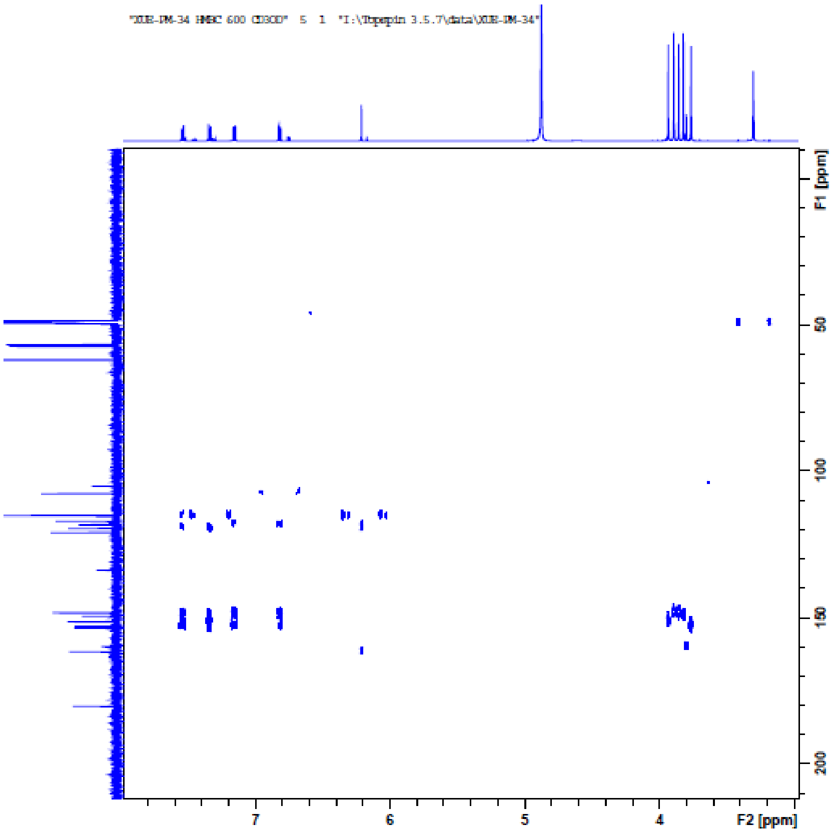
"XUB-PM-34 HMBC 600 CD3OD" 5 1 "I:\Topspin 3.5.7\data\XUB-PM-34"
F1 [ppm]
F2 [ppm]

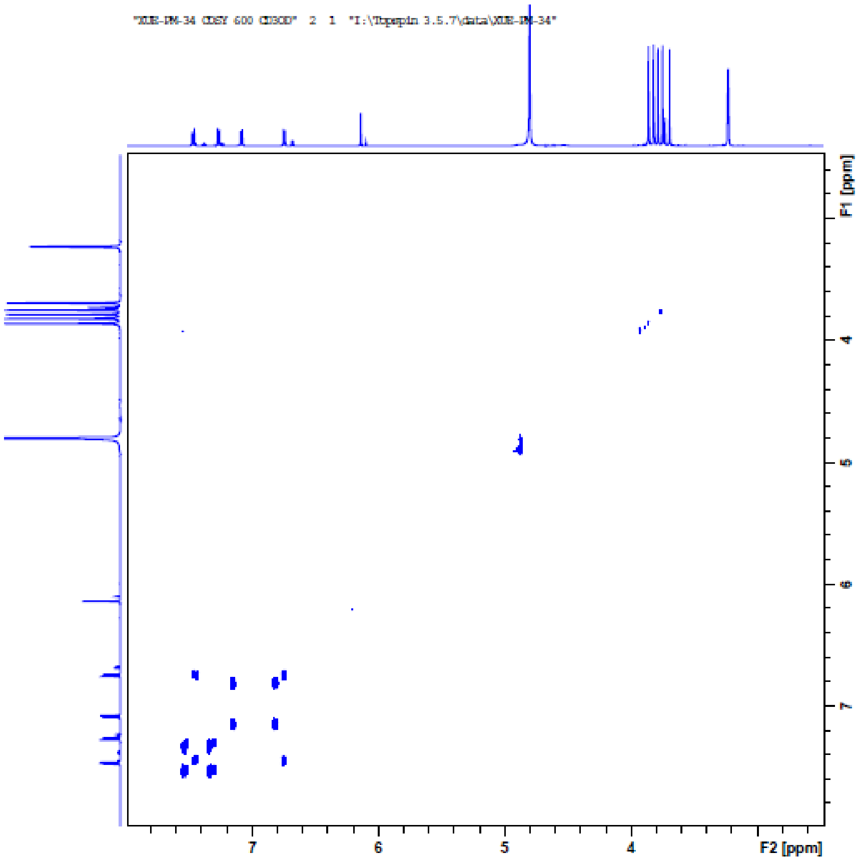
"XUE-PM-34 COSY 600 CD3OD" 2 1 "I:\Topspin 3.5.7\data\XUE-PM-34"
F1 [ppm]
F2 [ppm]
7
6
5
4

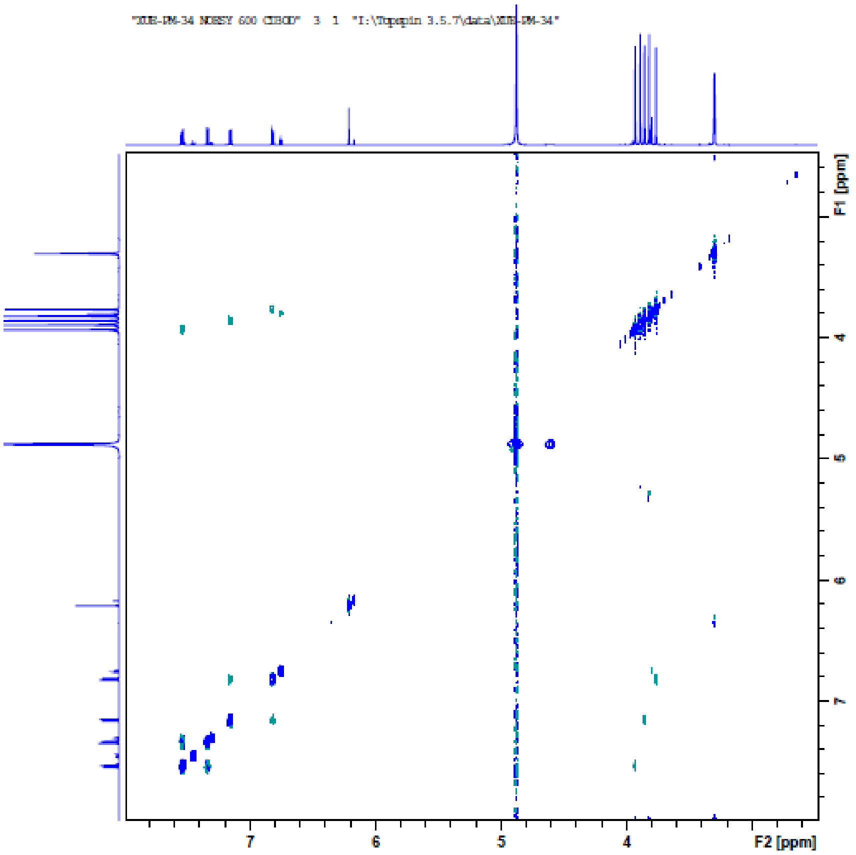
"XUE-PM-34 NOESY 600 CD3OD" 3 1 "I:\Topspin 3.5.7\data\XUE-PM-34"
F1 [ppm]
F2 [ppm]
7
6
5
4

Figures S24: 1D and 2D NMR spectra of compound 9 (methanol-*d*$_4$, 600MHz)

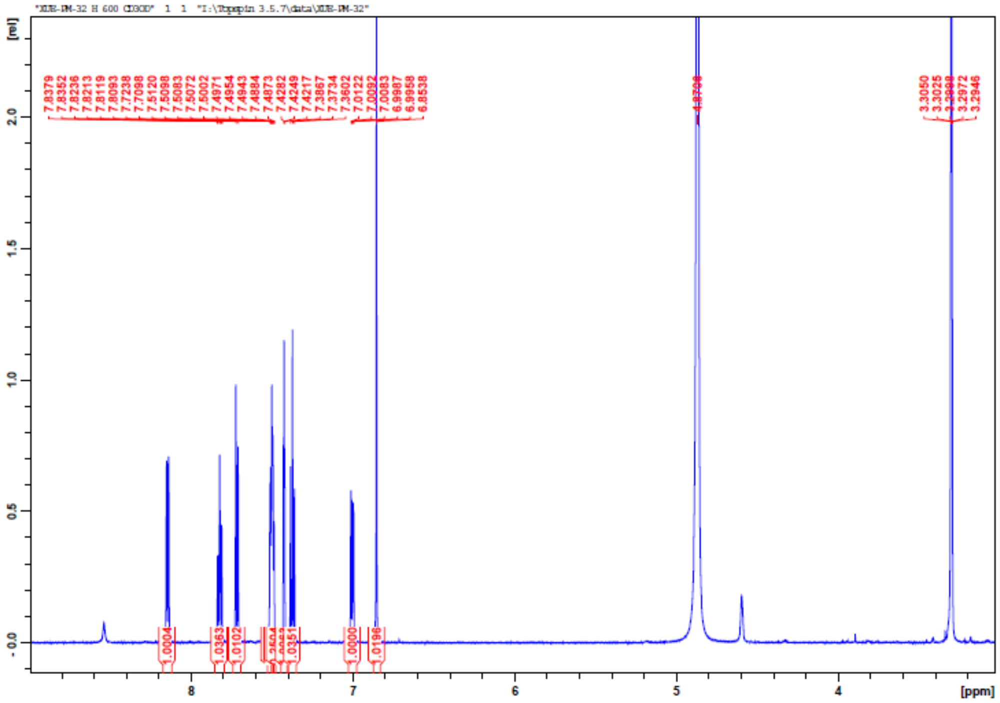

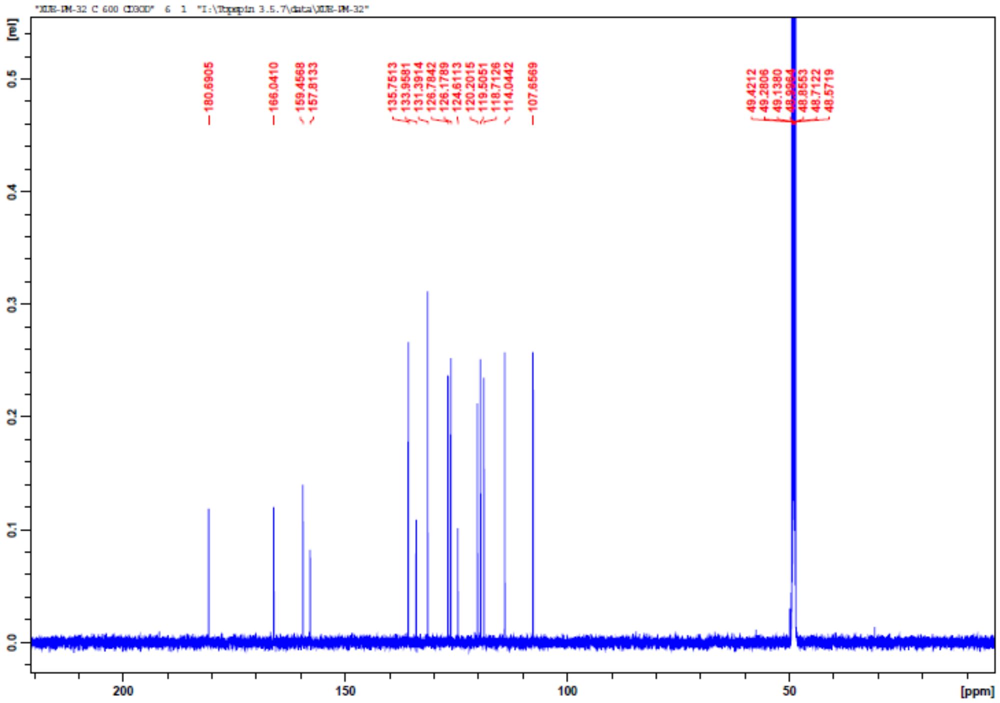

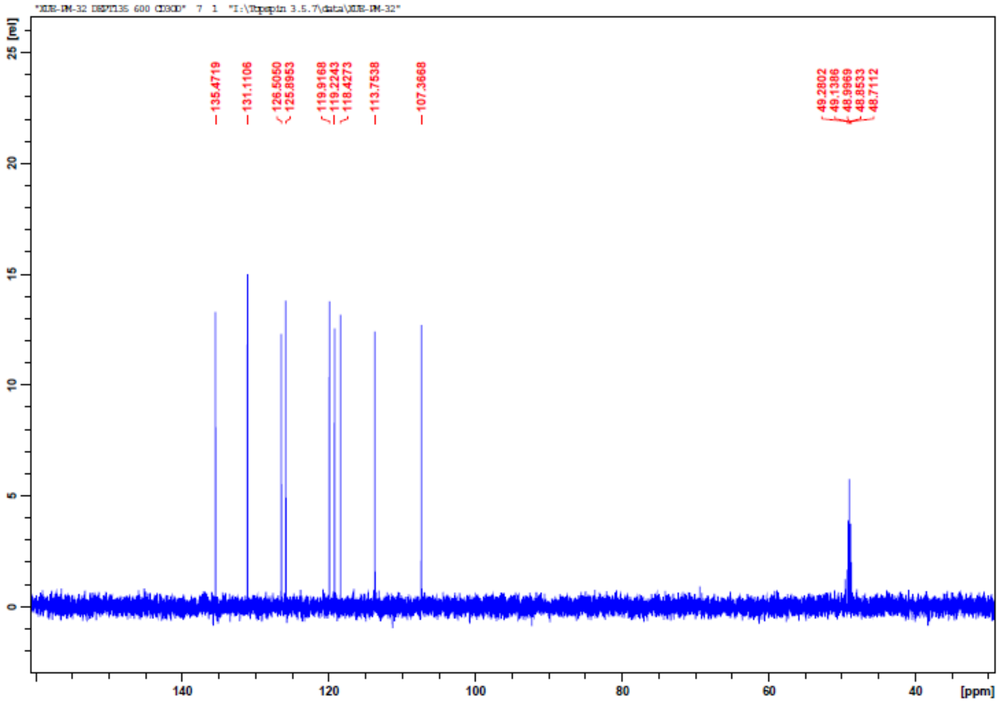
"XUB-PM-32 DEPT135 600 CD3OD" 7 1 "I:\Topspin 3.5.7\data\XUB-PM-32"
135.4719
131.1106
126.5050
125.8953
119.9168
119.2243
118.4273
113.7538
107.3668
49.2802
49.1386
48.9969
48.8533
48.7112
[rel]
[ppm]

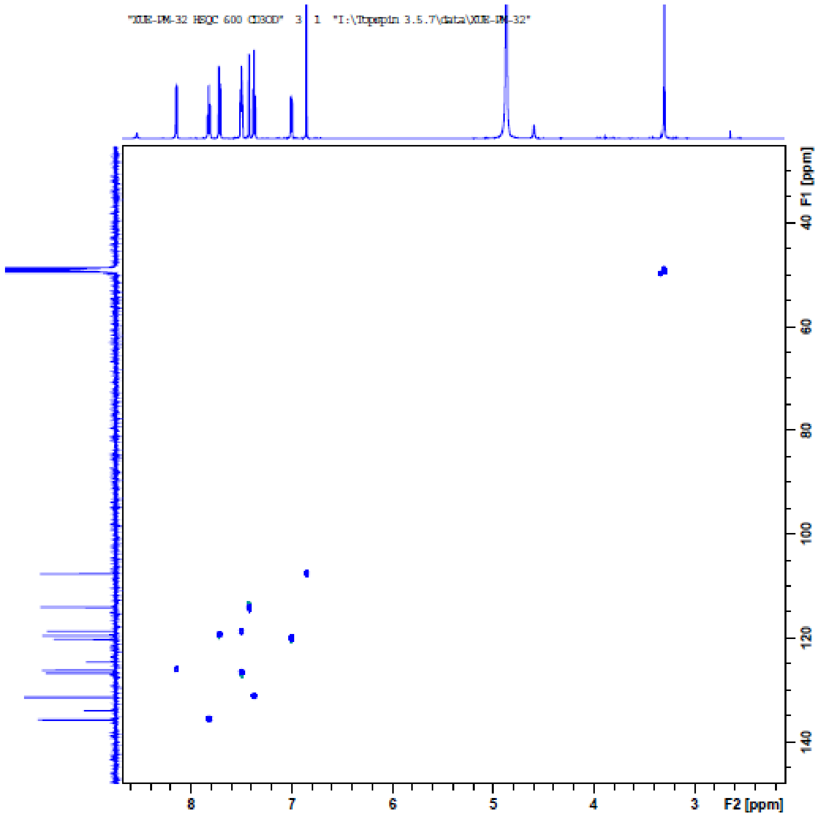
"XUB-PM-32 HSQC 600 CD3OD" 3 1 "I:\Topspin 3.5.7\data\XUB-PM-32"
F1 [ppm]
F2 [ppm]

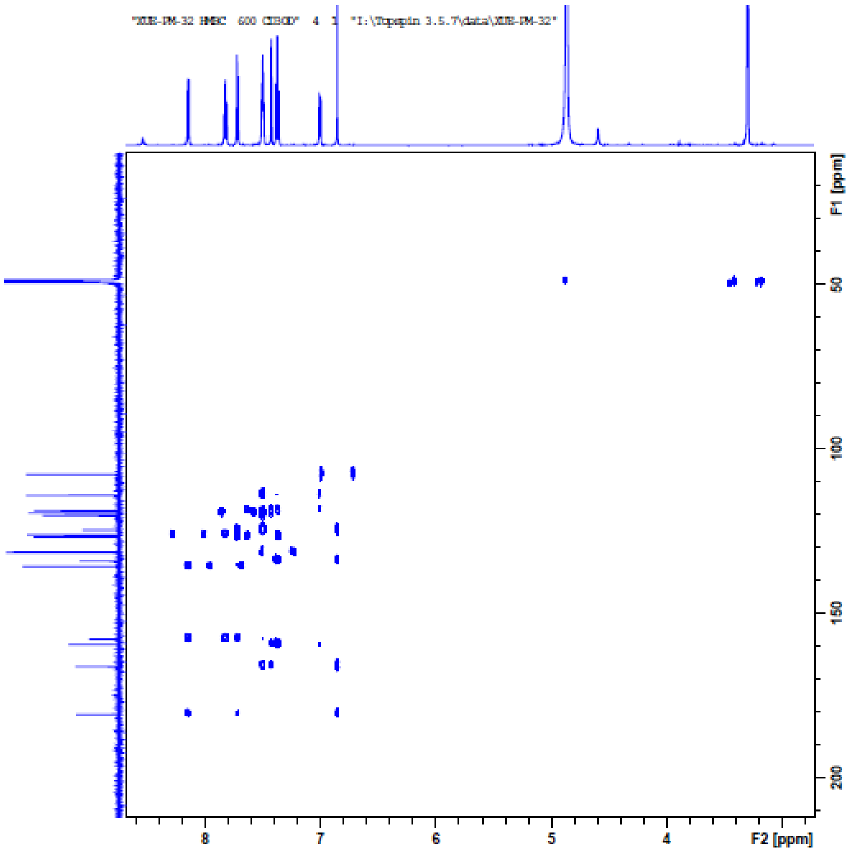
"XUB-PM-32 HMBC 600 CD3OD" 4 1 "I:\Topspin 3.5.7\data\XUB-PM-32"
F1 [ppm]
50
100
150
200
8
7
6
5
4
F2 [ppm]

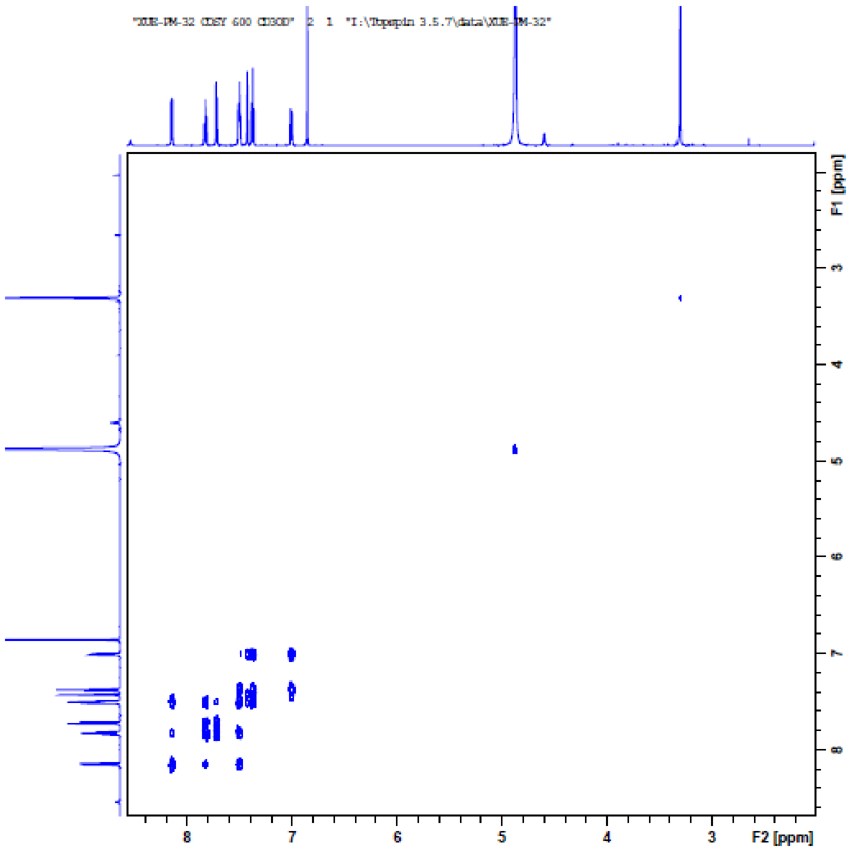
"XUB-PM-32 COSY 600 CD3OD" 2 1 "I:\Topspin 3.5.7\data\XUB-PM-32"
F1 [ppm]
3
4
5
6
7
8
8
7
6
5
4
3
F2 [ppm]

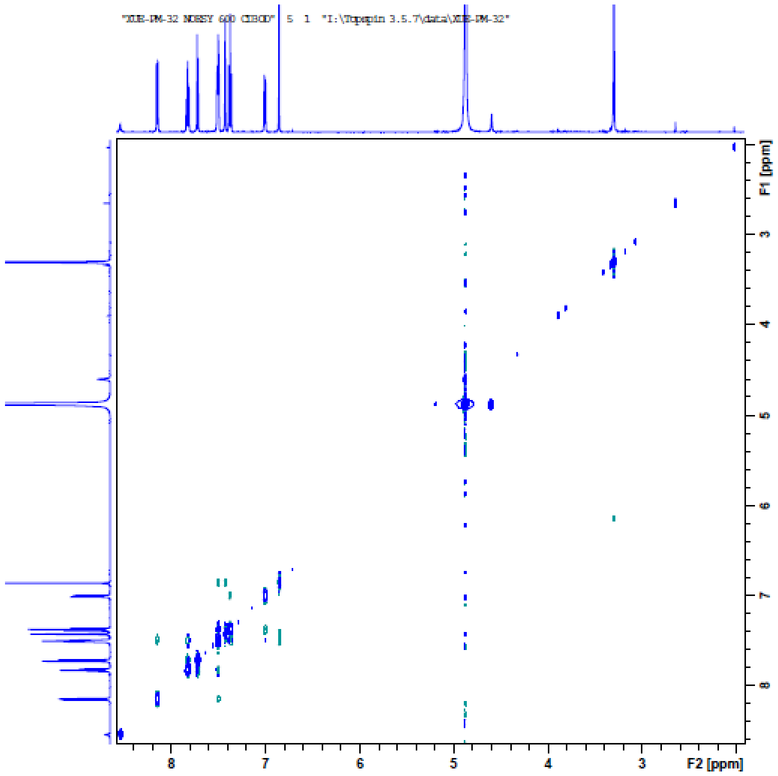

Figures S25: 1D and 2D NMR spectra of compound 10 (methanol-d_4, 600MHz)

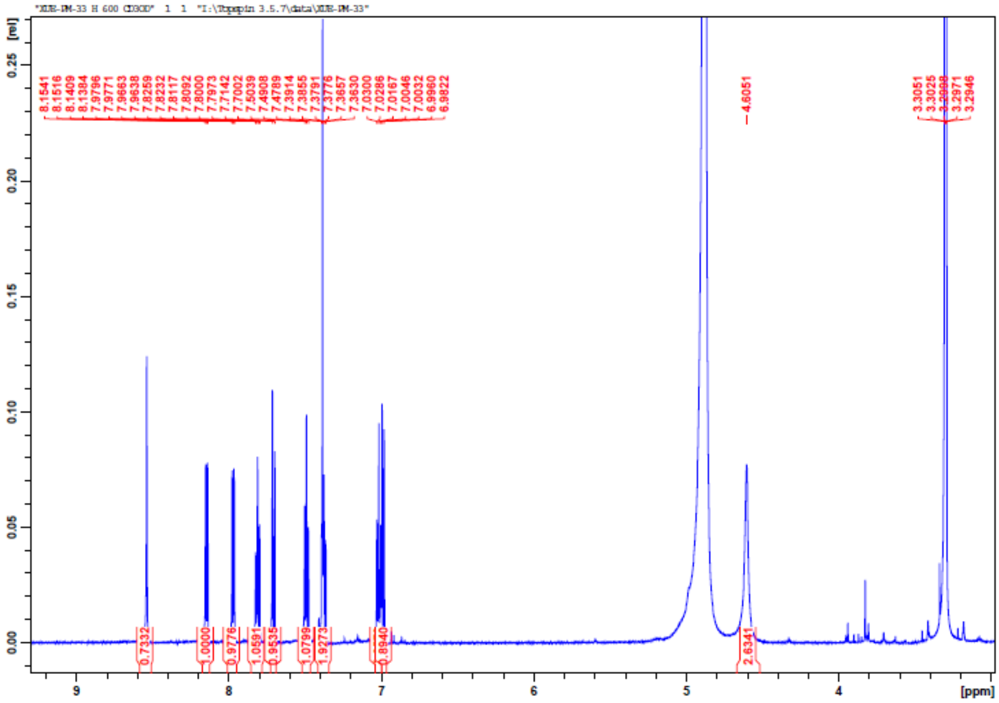

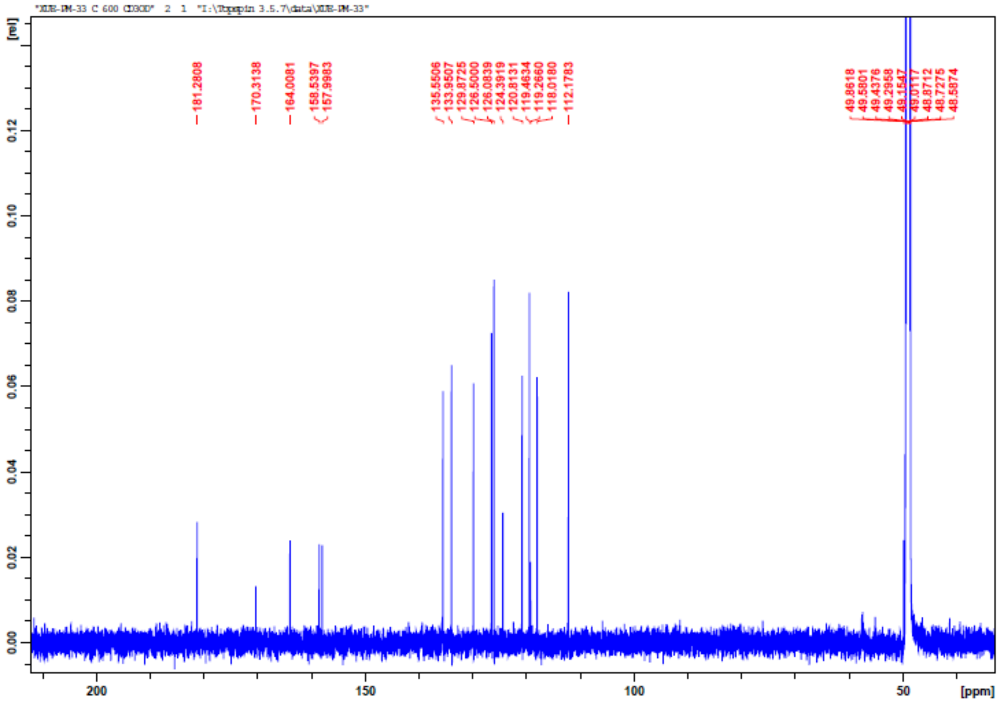

181.2808
170.3138
164.0081
158.5397
157.9983
135.5506
133.9507
129.8725
126.5000
126.0839
124.3919
120.8131
119.4634
119.2660
118.0180
112.1783
49.8618
49.5801
49.4376
49.2958
49.1547
49.0117
48.8712
48.7275
48.5874
[rel]
[ppm]

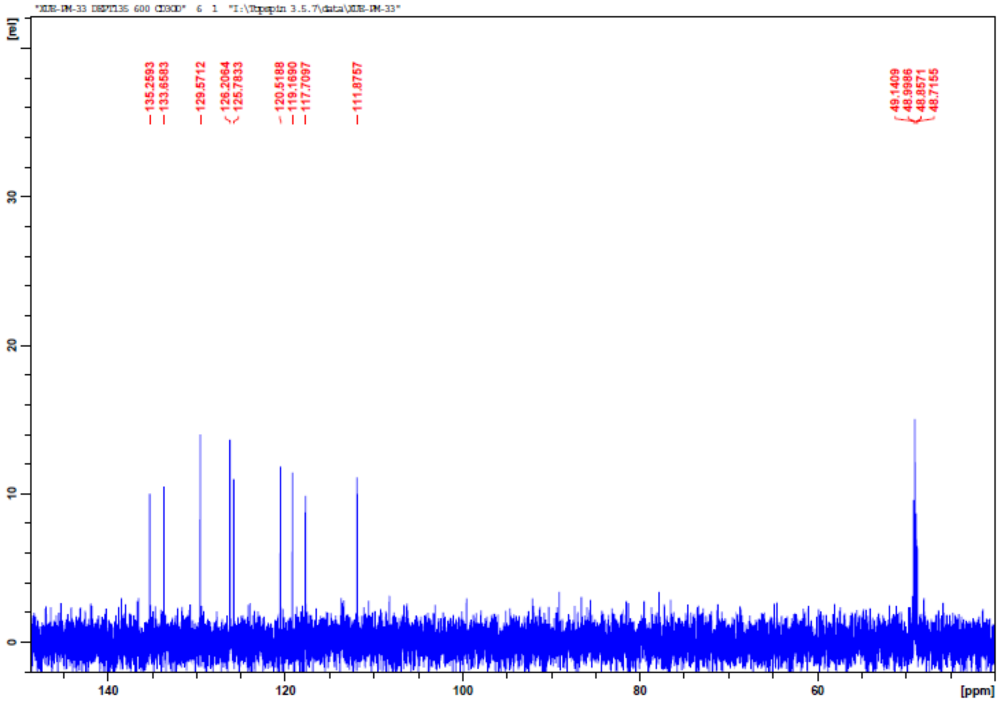

135.2593
133.6583
129.5712
126.2064
125.7833
120.5188
119.1690
117.7097
111.8757
49.1409
48.9986
48.8571
48.7155
[rel]
[ppm]

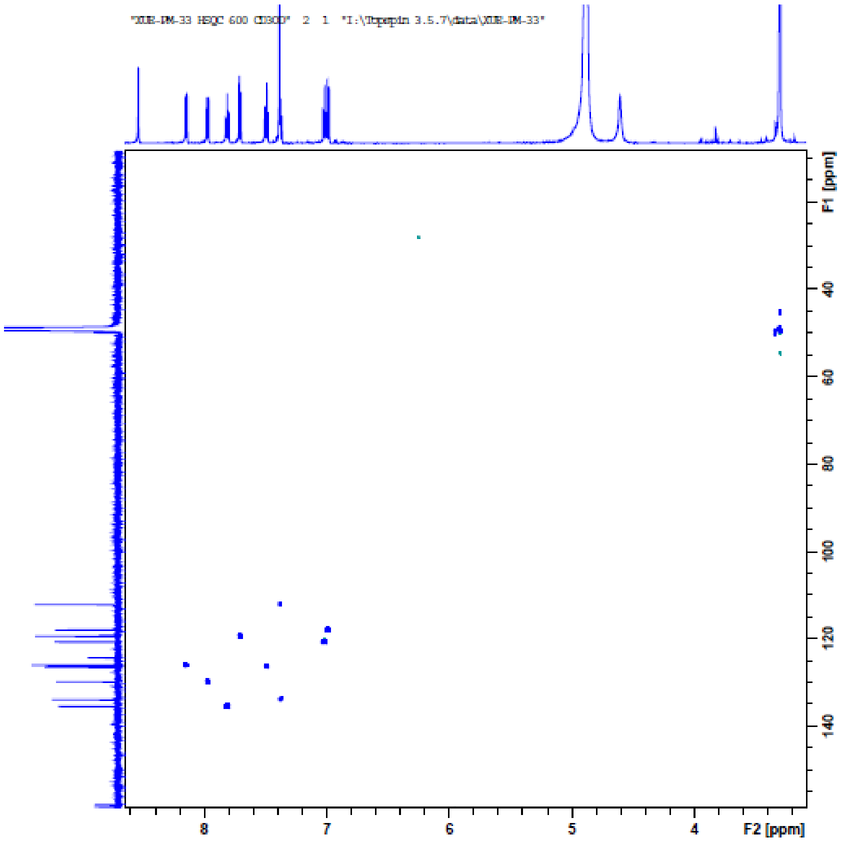
"XUB-PM-33 HSQC 600 CD3OD" 2 1 "I:\Topspin 3.5.7\data\XUB-PM-33"
F1 [ppm]
40
60
80
100
120
140
8
7
6
5
4
F2 [ppm]

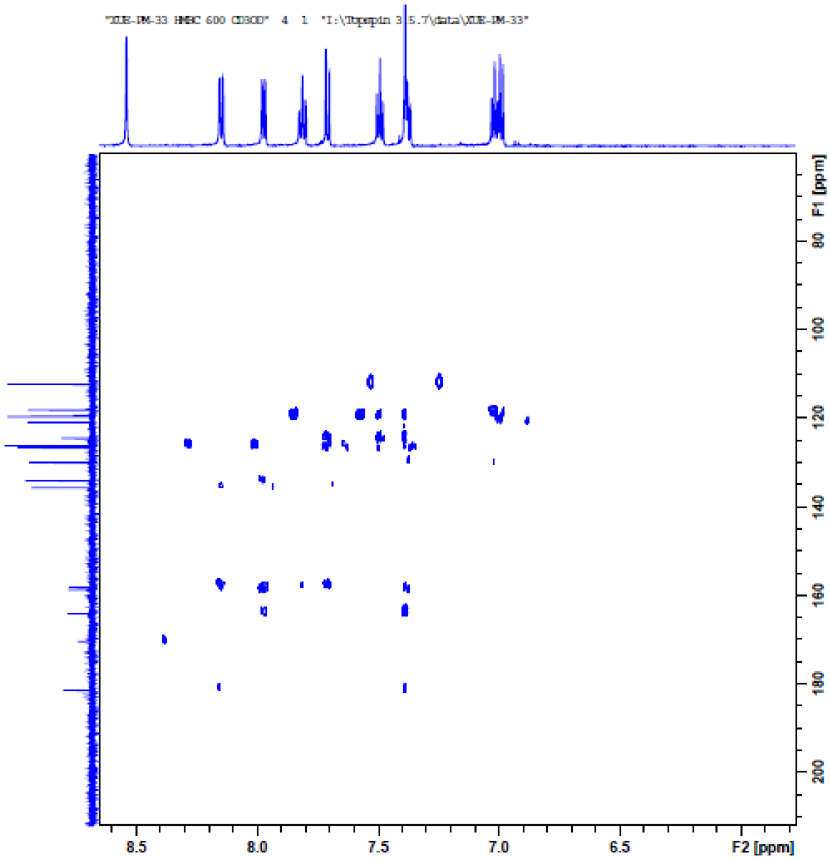
"XUB-PM-33 HMBC 600 CD3OD" 4 1 "I:\Topspin 3.5.7\data\XUB-PM-33"
F1 [ppm]
80
100
120
140
160
180
200
8.5
8.0
7.5
7.0
6.5
F2 [ppm]

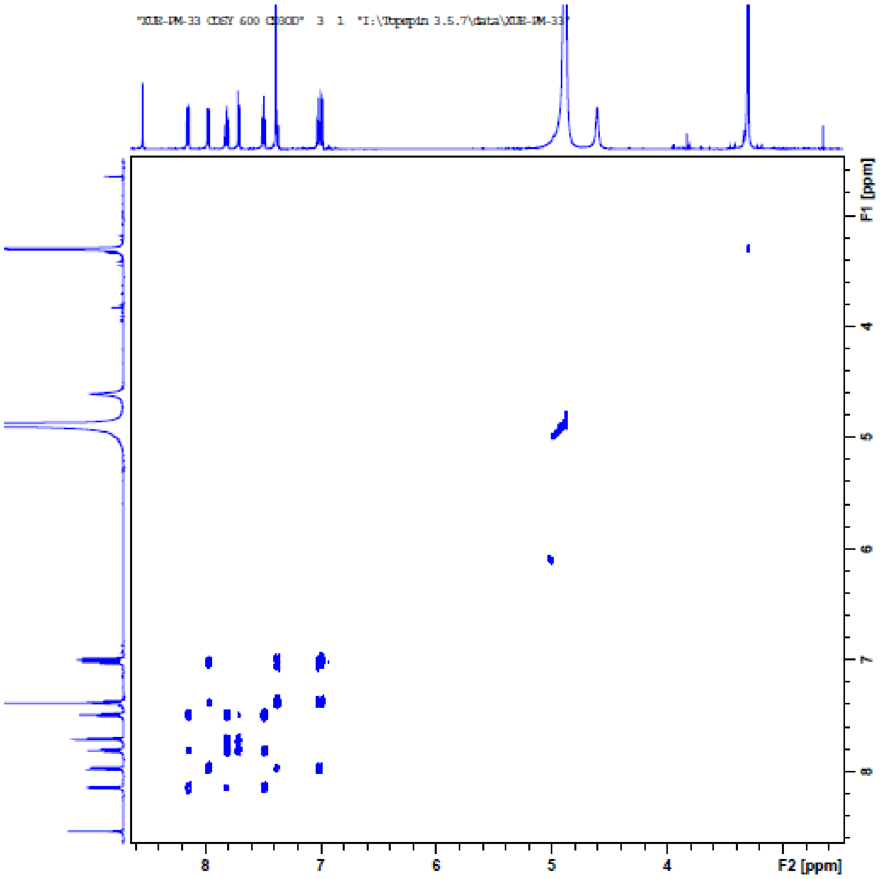
"XUE-PM-33 COSY 600 CD3OD" 3 1 "I:\Topspin 3.5.7\data\XUE-PM-33"
F1 [ppm]
F2 [ppm]

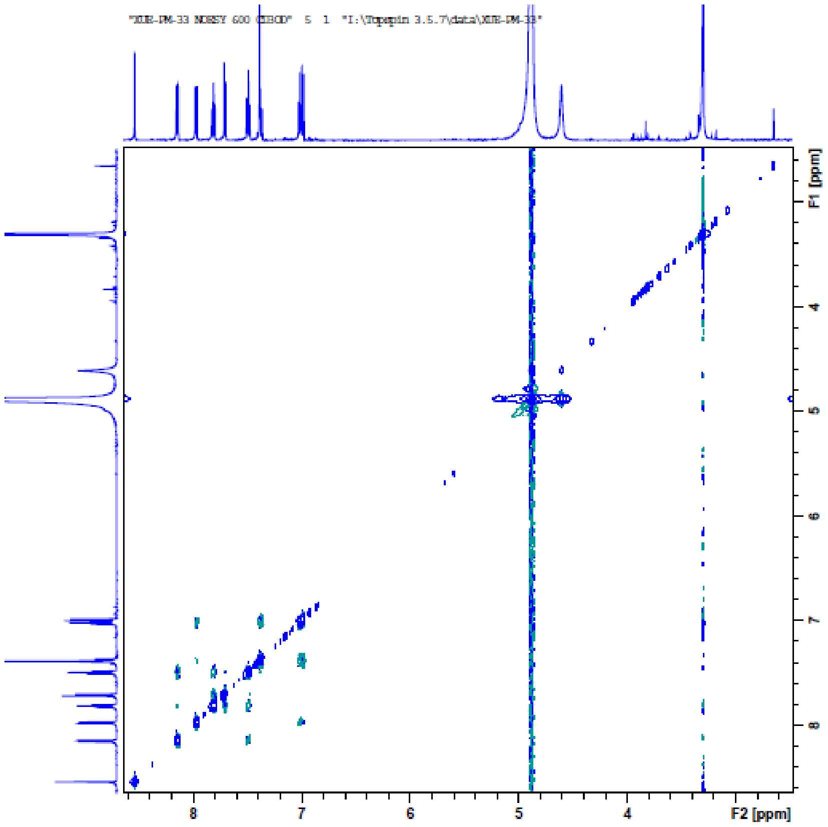
"XUE-PM-33 NOESY 600 CD3OD" 5 1 "I:\Topspin 3.5.7\data\XUE-PM-33"
F1 [ppm]
F2 [ppm]

Figures S26: 1D and 2D NMR spectra of compound 12 (methanol-d_4, 600MHz)

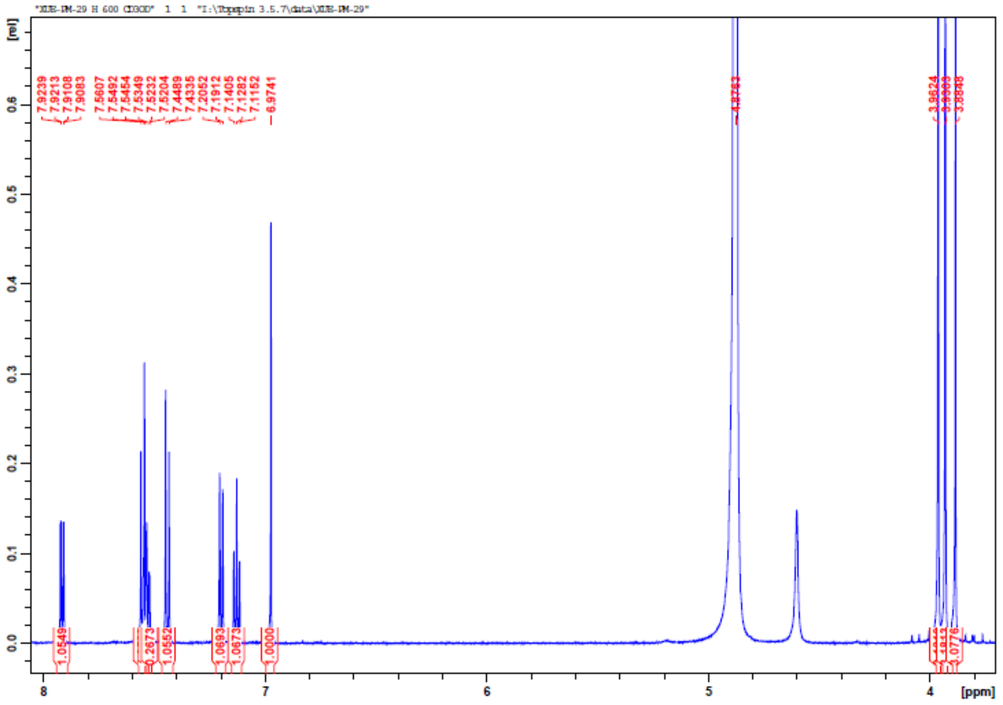

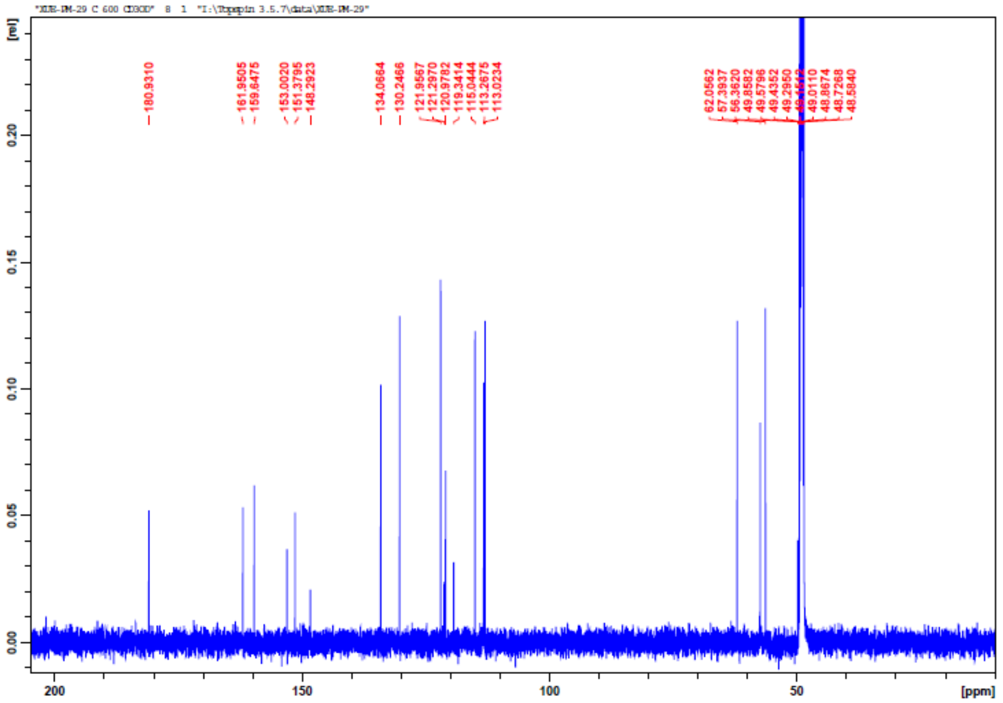

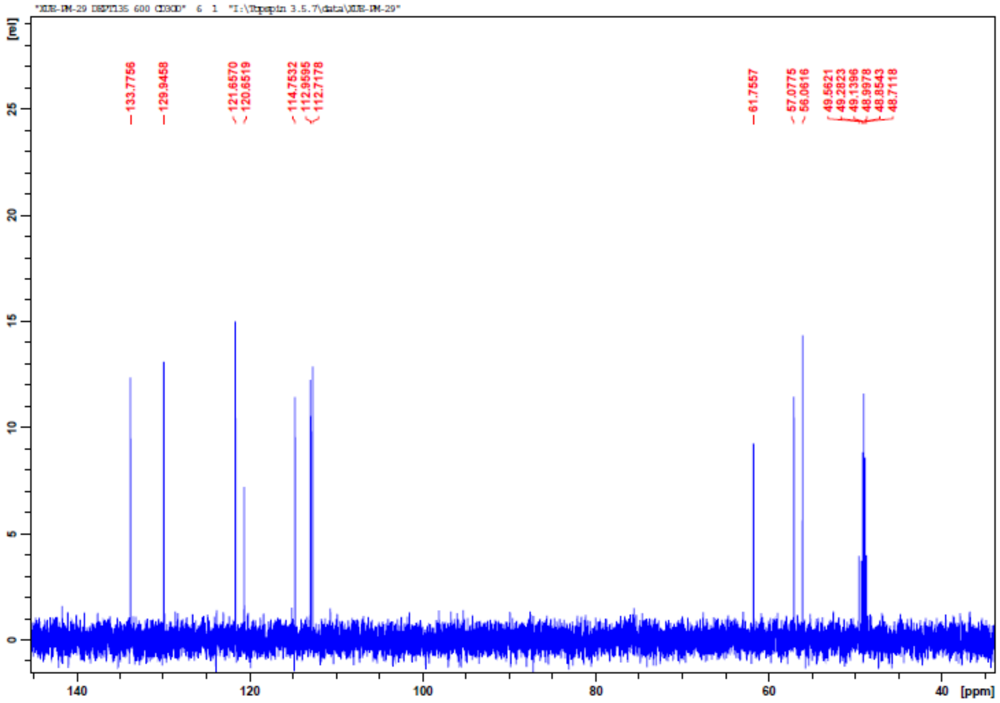
"XUE-PM-29 DEPT135 600 CD3OD" 6 1 "I:\Topspin 3.5.7\data\XUE-PM-29"
133.7756
129.9458
121.6570
120.6519
114.7532
112.9595
112.7178
61.7557
57.0775
56.0616
49.5621
49.2823
49.1396
48.9978
48.8543
48.7118
[rel]
[ppm]

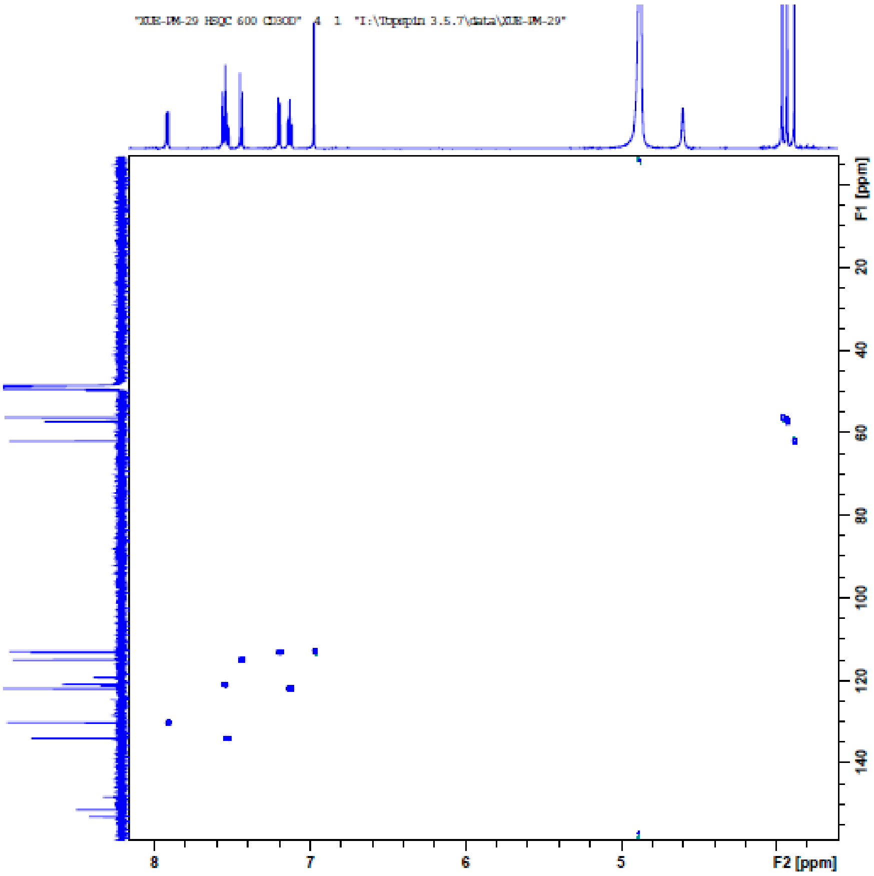
"XUE-PM-29 HSQC 600 CD3OD" 4 1 "I:\Topspin 3.5.7\data\XUE-PM-29"
F1 [ppm]
F2 [ppm]

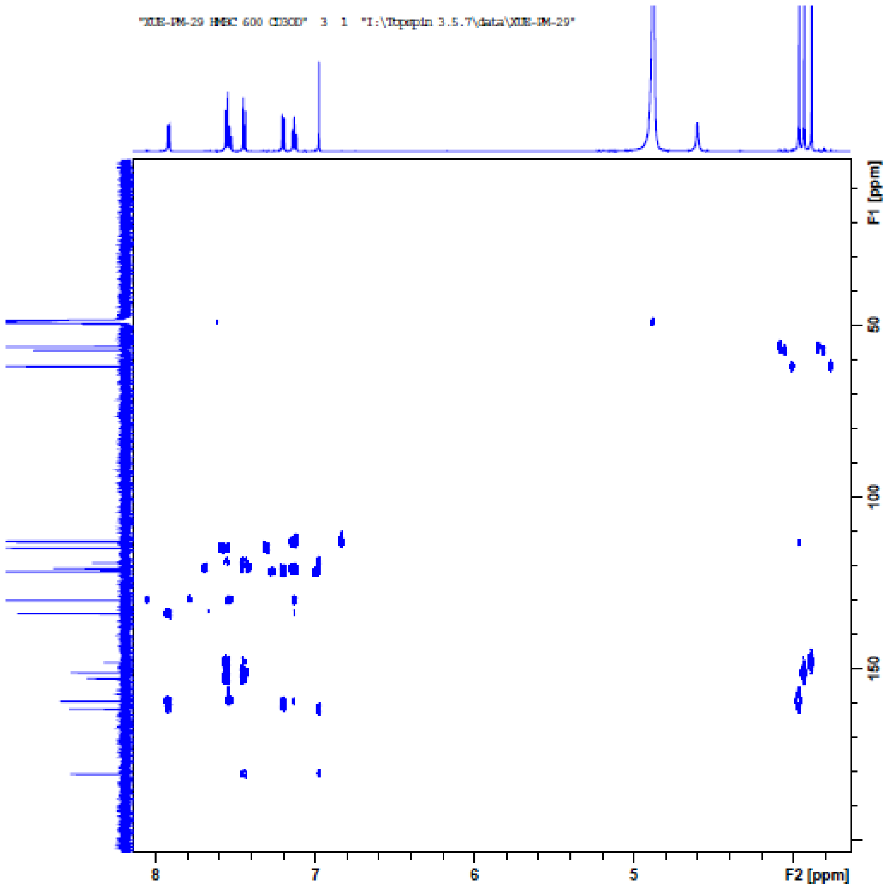
"XUE-PM-29 HMBC 600 CD3OD" 3 1 "I:\Topspin 3.5.7\data\XUE-PM-29"
F1 [ppm]
50
100
150
8
7
6
5
F2 [ppm]

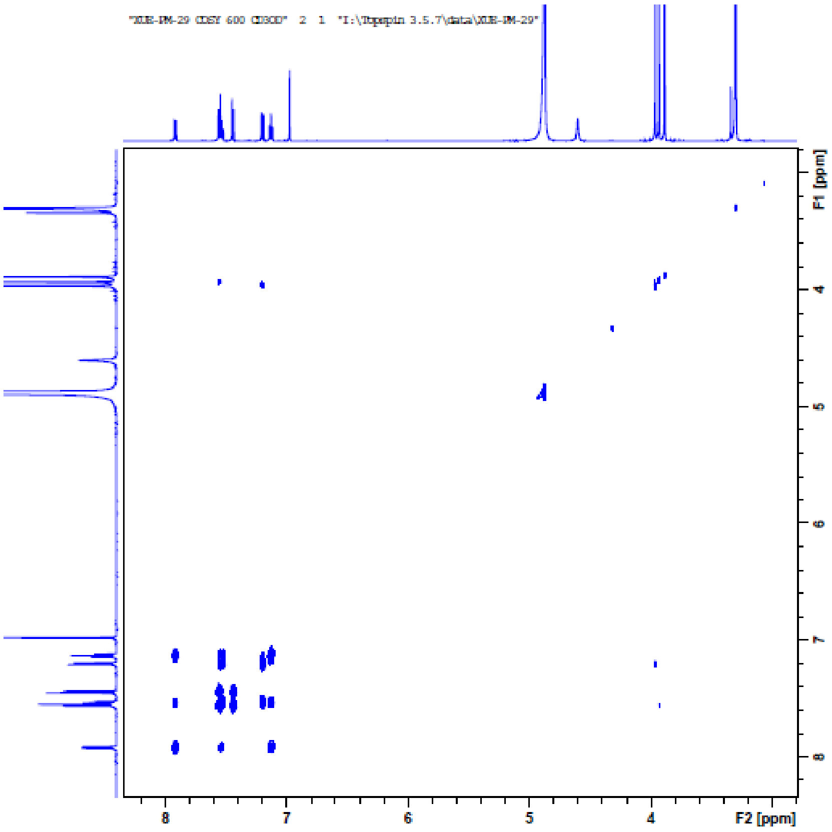
"XUE-PM-29 COSY 600 CD3OD" 2 1 "I:\Topspin 3.5.7\data\XUE-PM-29"
F1 [ppm]
4
5
6
7
8
8
7
6
5
4
F2 [ppm]

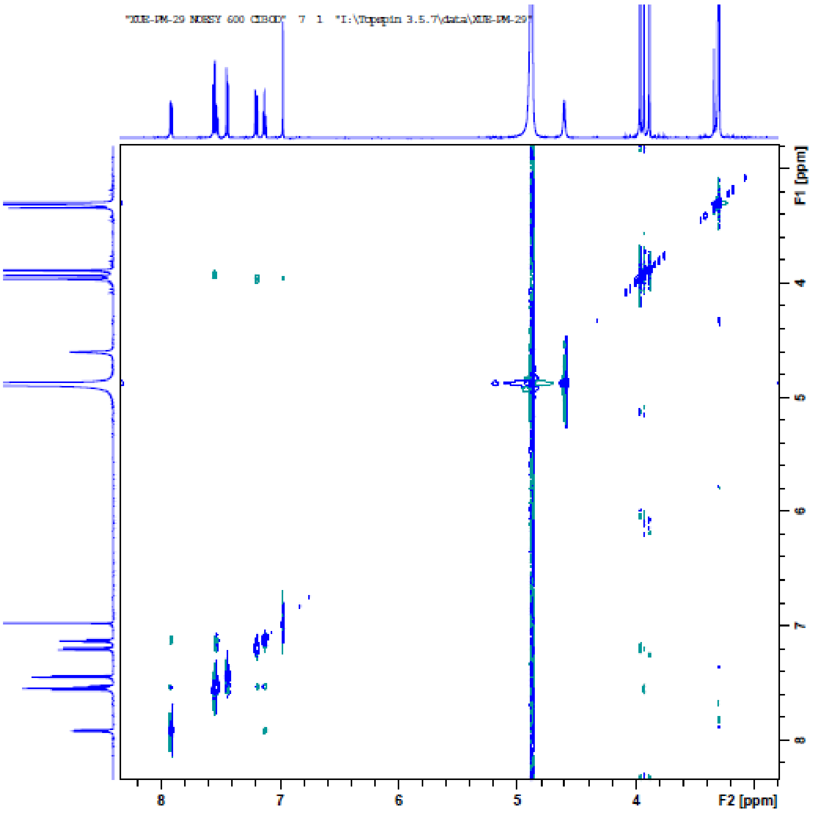
"XUE-PM-29 NOESY 600 CD3OD" 7 1 "I:\Topspin 3.5.7\data\XUE-PM-29"
F1 [ppm]
F2 [ppm]
8
7
6
5
4

Table S1 ^{13}C (150 MHz) and ^{1}H (600 MHz) NMR data of Compounds 1-4 (δ in ppm, J in Hz, $CDCl_3$)[a]

	**1**		**2**		**3**		**4**	
no.	δ_C, type	δ_H	δ_C, type	δ_H	δ_C, type	δ_H	δ_C, type	δ_H
2	163.3, C		163.6, C		161.0, C		160.6, C	
3	107.7, CH	6.83, s	107.5, CH	6.86, s	112.5, CH	7.16, s	112.7, CH	7.19, s
4	178.5, C		178.5, C		178.9, C		178.9, C	
5	125.7, CH	8.21, d (7.9)	125.7, CH	8.23, dd (7.9, 1.6)	125.6, CH	8.21, dd (7.9, 1.5)	125.6, CH	8.21, d (8.0)
6	125.3, CH	7.41	125.3, CH	7.42, td (7.9, 0.9)	124.9, CH	7.38, t (7.9)	125.0, CH	7.39, dd (8.0, 7.3)
7	133.8, CH	7.69, t-like (8.3)	133.9, CH	7.70, ddd (8.4, 7.9, 1.6)	133.6, CH	7.66, ddd (7.9, 8.4, 1.5)	133.6, CH	7.66, dd (7.3, 8.4)
8	118.1, CH	7.56, d (8.3)	118.1, CH	7.57, dd (8.4, 0.9)	118.0, CH	7.51, d (8.4)	118.0, CH	7.51, d (8.4)
9	156.2, C		156.3, C		156.5, C		156.4, C	
10	123.9, C		123.8, C		123.7, C		123.7, C	
1'	133.1, C		131.7, C		120.8, C		121.3, C	
2'	111.8, CH	7.43	126.3, CH	7.93, dd (7.8, 1.6)	158.0, C		152.4, C	
3'	160.0, C		129.1, CH	7.51	111.8, CH	7.02, d (7.8)	113.0, CH	6.96, d (9.0)

				7.53				
4'	117.2, CH	7.06, d (8.2)	131.7, CH		132.5, CH	7.46, td (7.8, 1.6)	117.5, CH	7.01, dd (9.0, 2.2)
5'	130.1, CH	7.42	129.1, CH	7.51	120.7, CH	7.09, t (7.8)	153.5, C	
6'	118.8, CH	7.50, d (7.5)	126.3, CH	7.93, dd (7.8, 1.6)	129.3, CH	7.88, dd (7.8, 1.6)	114.6, CH	7.44, d (2.2)
2'-OCH_3					55.7, CH_3	3.92, s	56.2, CH_3	3.88, s
3'-OCH_3	55.5, CH_3	3.88, s						
5'-OCH_3							55.9, CH_3	3.83, s

[a] The signal not marked with split condition was due to overlap. The assignment of all signals was based on comprehensive analysis of ^{1}H, ^{13}C, DEPT135, COSY, HSQC, HMBC, and NOESY NMR spectra.

Table S2 ^{13}C (150 MHz) and ^{1}H (600 MHz) NMR data of Compounds 6-9 (δ in ppm, J in Hz, methanol-d_4)

	**6**		**7**		**8**		**9**	
no.	δ_C, type	δ_H	δ_C, type	δ_H	δ_C, type	δ_H	δ_C, type	δ_H
2	165.8, C		162.2, C		161.7, C		166.0, C	
3	107.2, CH	6.80, s	115.4, CH	6.17, s	115.1, CH	6.21, s	107.7, CH	6.85, s
4	180.6, C		180.6, C		180.3, C		180.7, C	
5	126.1, CH	8.11, dd (7.9, 1.6)	148.3, C		148.4, C		126.2, CH	8.15, dd (8.0, 1.6)
6	126.7, CH	7.48, dd (7.9, 8.0)	151.3, C		151.4, C		126.8, CH	7.50[a]
7	135.6, CH	7.80, ddd (8.2, 8.0, 1.6)	120.8, CH	7.52, d (9.3)	120.9, CH	7.54, d (9.3)	135.8, CH	7.82, ddd (8.0, 8.0, 1.6)
8	119.5, CH	7.70, d (8.2)	115.2, CH	7.30, d (9.3)	115.1, CH	7.34, d (9.3)	119.5, CH	7.72, d (8.0)
9	157.7, C		153.7, C		153.4, C		157.8, C	
10	124.5, C		119.5, C		119.5, C		124.6, C	
1'	128.0, C		112.2, C		118.3, C		134.0, C	
2'	109.0, CH	7.16, d (2.1)	159.9, C		149.5, C		114.0, CH	7.42, dd (2.0, 2.0)
3'	152.3, C		105.1, CH	6.75, d (8.5)	148.4, C		159.5, C	

4'	141.3, C		133.7, CH	7.45, t (8.5)	117.1, CH	7.15, d (9.1)	120.2, CH	7.00, dd (8.0, 2.0)
5'	155.1, C		105.1, CH	6.75, d (8.5)	107.7, CH	6.84, d (9.1)	131.4, CH	7.37, dd (8.0, 8.0)
6'	103.3, CH	7.13, d (2.1)	159.9, C		153.0, C		118.7, CH	7.50[a]
5-OCH_3			62.0, CH3	3.88, s	62.1, CH3	3.89, s		
6-OCH_3			57.4, CH3	3.93, s	57.4, CH3	3.93, s		
2'-OCH_3			56.6, CH3	3.80, s	61.9, CH3	3.82, s		
3'-OCH_3					57.0, CH3	3.86, s		
4'-OCH_3	61.1, CH_3	3.87, s						
5'-OCH_3	56.7, CH_3	3.94, s						
6'-OCH_3			56.6, CH3	3.80, s	56.8, CH3	3.76, s		

[a] Overlapped. The assignment of all signals was based on comprehensive analysis of ^1H, ^{13}C, DEPT135, COSY, HSQC, HMBC, and NOESY NMR spectra.

Table S3 ^{13}C (150 MHz) and ^{1}H (600 MHz) NMR data of Compounds 10 and 12

(δ in ppm, J in Hz, methanol-d_4)

	10		12	
no.	δ_C, type	δ_H	δ_C, type	δ_H
2	164.0. C		162.0, C	
3	112.2, CH	7.39, s	113.0, CH	6.97, s
4	181.3, C		180.9, C	
5	126.1, CH	8.15, dd (7.9, 1.5)	148.3, C	
6	126.5, CH	7.49, t-like (7.9)	151.4, C	
7	135.6, CH	7.81, ddd (8.5, 7.9, 1.5)	121.0, CH	7.55, d (9.2)
8	119.5, CH	7.71, d (8.5)	115.0, CH	7.44, d (9.2)
9	158.0, C		153.0, C	
10	124.4, C		119.3, C	
1'	119.3, C		121.3, C	
2'	158.5, C		159.6, C	
3'	118.0, CH	6.99, d (8.3)	113.3, CH	7.20, d (7.9)
4'	134.0, CH	7.38, ddd (8.3, 8.0, 1.5)	134.1, CH	7.53, td (7.9, 1.6)
5'	120.8, CH	7.02, t-like (8.0)	122.0, CH	7.13, t (7.9)
6'	129.9, CH	7.97, dd (8.0, 1.5)	130.2, CH	7.92, dd (7.9, 1.6)
5-OCH_3			62.1, CH_3	3.88, s
6-OCH_3			57.4, CH_3	3.93, s
2'-OCH_3			56.4,CH_3	3.96, s
